# Neonatal oral fluid as a transmission route for bifidobacteria to the infant gut immediately after birth

**DOI:** 10.1038/s41598-019-45198-9

**Published:** 2019-06-18

**Authors:** Kazuya Toda, Ken Hisata, Takumi Satoh, Noriko Katsumata, Toshitaka Odamaki, Eri Mitsuyama, Takane Katayama, Tetsuya Kuhara, Kohzo Aisaka, Toshiaki Shimizu, Jin-zhong Xiao

**Affiliations:** 1 Next Generation Science Institute R&D Division, Morinaga Milk Industry Co., Ltd., Kanagawa, 252-8583 Japan; 2 Department of Pediatrics and Adolescent Medicine, Juntendo University Graduate School of Medicine, Tokyo, 113-8421 Japan; 3 Department of Molecular Microbiology, Tokyo University of Agriculture, Tokyo, 183-0051 Japan; 4 Division of Integrated Life Science, Graduate School of Biostudies, Kyoto University, Sakyo-ku, Kyoto, 606-8502 Japan; 5 Department of Obstetrics and Gynecology, Hamada Hospital, Tokyo, 101-0062 Japan

**Keywords:** Clinical microbiology, Comparative genomics

## Abstract

Bifidobacteria are one of the most abundant bacterial groups in the infant gut microbiota and are closely associated with infant health and can potentially affect health in later life. However, the details regarding the source of bifidobacteria have yet to be completely elucidated. This study aimed to assess neonatal oral fluid (OF) as a transmission route for bifidobacteria to the infant gut during delivery. Neonatal OF and infant feces (IF) were collected immediately and one month after birth from 15 healthy vaginally delivered newborns. *Bifidobacterium* strains were isolated from OF and IF samples, and the similarity of strains between the OF-IF pairs was evaluated based on the average nucleotide identity (ANI) value. The 16S rRNA gene sequencing results revealed the presence of Bifidobacteriaceae at >1% relative abundance in all OF samples. *Bifidobacterium* strains were isolated from OF (9/15) and IF (11/15) samples, and those sharing high genomic homology (ANI values >99.5%) between the neonatal OF and IF samples were present in one-third of the OF-IF pairs. The results of this study indicate that viable bifidobacteria are present in neonatal OF and that OF at birth is a possible transmission route of bifidobacteria to the infant gut.

## Introduction

The infant gut microbiota are crucial for infant health and may affect health status in later life^1,2^. *Bifidobacterium* is one of the most abundant bacterial genera in the gut microbiota of infants^3^ and is believed to exert beneficial effects on the maturation of the immune, digestive and metabolic systems^4^, as well as in the protection against pathogens^5^ and the prevention of allergies^6^. Although several studies have been conducted on the transmission routes of bacteria, including bifidobacteria, and the development of infant gut microbiota, the details of this transmission remain unclear^7–10^.

The establishment of this microbial ecosystem occurs from birth and is influenced by various factors, such as type of infant feeding, birth mode, etc. The microbiota of breastfed infants become dominated by the obligate anaerobes *Bifidobacterium* and/or *Bacteroides* during the first week of life^11–13^. Furthermore, human milk has been reported to be a source of commensal bacteria that may colonize the infant gut^14–16^, and a previous study indicated the presence of bifidobacterial strains in maternal milk, suggesting the possibility of breast milk as a transmission route of bifidobacteria from the mother to the infant gut^8^. However, the researchers observed that none of these monophyletic strains were isolated earlier from the mother’s milk than from the infant’s feces and that no strain was isolated from the human mother’s milk collected before delivery or the colostrum. These results suggest that human milk is a reservoir of bifidobacteria but raises the question of whether human milk is the initial source of microbes for infants.

In recent years, the delivery mode of microbes has been demonstrated to potently impact early microbial colonization in the infant gut^17,18^. *Makino et al*. reported the transmission of *Bifidobacterium* strains from the mother and their colonization of the infant intestine shortly after birth among infants born vaginally^9^. In fact, cesarean section (C-section) was shown to be associated with a lower abundance of bifidobacteria in the infant gut than was vaginal birth^7,9^. These results suggest that the birth canal is an important source of maternal bacteria, including bifidobacteria, during the delivery process. Although, the uterus was previously believed to be sterile, the results of recent studies have suggested that there are bacteria in the uterine environment^19–22^. The fetus has been suggested to be exposed to microbes through amniotic fluid that is continuously swallowed from mid- to late gestation *in utero*^23^. In addition, when vaginally delivered infants pass through the birth canal, their oral and nasal cavities are infiltrated with vaginal secretions^24–26^. Therefore, we speculated that neonatal oral fluid (OF) at delivery is an important source of bacteria from both maternal and environmental ecosystems and may contribute to the early delivery of bacteria to the digestive tracts of neonates.

The goal of the present study was to assess the potential of OF as a route of transmission for *Bifidobacterium* to neonates immediately after birth. We first assessed the presence of *Bifidobacterium* in OF by using molecular approaches. Then, we tried to isolate *Bifidobacterium* strains from OF and infant fecal (IF) samples to identify the same strains in OF-IF pairs. In addition, since the number of bacteria was very low in OF, these samples were precultivated in breast milk (BM) and YCFA to increase the efficiency of bifidobacterial isolation, where cultivation using BM medium was expected to promote the selective growth of *Bifidobacterium*^3^, while YCFA medium was expected to support the growth of most gut bacterial species^27^. Using these unique strategies, we analyzed the microbiotic profile of OF and confirmed the presence of viable bifidobacteria. Furthermore, we succeeded in isolating *Bifidobacterium* strains from neonatal OF and IF samples and in identifying the strains with high genomic homology in OF-IF pairs.

## Results

### Collection and bacterial monitoring of neonatal OF

Fifteen healthy mother-vaginally delivered child pairs were enrolled in this study (Table 1). The average cell numbers of total bacteria and bifidobacteria in neonatal OF were log 5.71/mL and log 3.59/mL (positive for eight among 15 subjects, qPCR-positive: 53%), respectively, based on the qPCR analysis results (Supplemental Table 1).

**Table 1:** General information on the subjects.

Subject ID	Gestational age at birth (weeks)	Maternal defecation during delivery	Infant gender	Body weight at birth (g)	Breastfeeding vs Formula feeding for 1 month after birth
1	40	−	female	3450	3: 1
2	38	+	female	2660	1: 1
3	39	−	female	3510	7: 3
4	38	−	male	3475	1: 0
5	39	−	female	3325	4: 1
6	41	+	female	2900	7: 3
7	40	−	male	4120	1: 0
8	39	−	male	3110	1: 0
9	39	−	female	2760	1: 0
10	39	−	female	3280	1: 0
11	38	−	female	2780	1: 0
12	39	+	female	3135	1: 0
13	41	+	male	3595	1: 2
14	40	−	female	2925	9: 1
15	38	−	male	3480	1: 0

**Figure 1:**
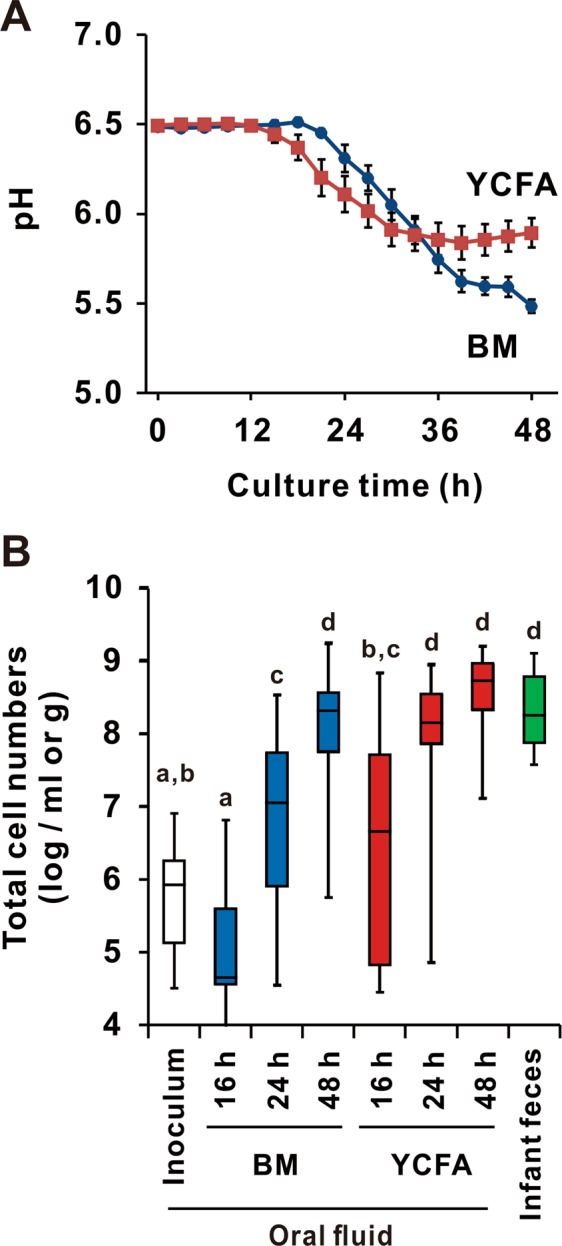
Changes in pH values and cell numbers in OF during cultivation. Neonatal OF was cultured with breast milk (BM, blue line) or YCFA (red line) media for 48 h in pH-stat fermenters. (**A**) The pH value was continuously monitored, and each point represents the mean value with the S.E. (n = 15). (**B**) qPCR was performed to evaluate total cell numbers. The limit of detection was 10^4^ cells/mL or g. Groups without the same letter were statistically different using the Tukey-Kramer test (p < 0.05).

**Figure 2:**
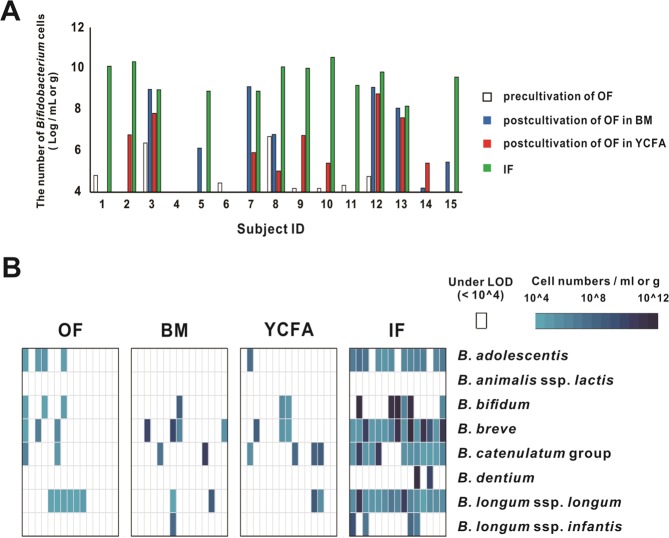
Abundance and prevalence of *Bifidobacterium* species in OF and IF samples. (**A**) The abundance of *Bifidobacterium* was evaluated using qPCR. The limit of detection was 10^4^ cells/mL or g. (**B**) A heat map showing the relative abundances of *Bifidobacterium* species in light to deep blue for low to high abundance, respectively. Each vertical lane in each category indicates each ID in consecutive order from left to right. Samples under the limit of detection (LOD, 10^4^ cells/mL or g) are indicated in white. Abbreviations: precultivated neonatal oral fluid (OF), postcultivated OF in BM (BM) or YCFA (YCFA) for 48 h and infant fecal (IF) samples.

To increase the efficiency of *Bifidobacterium* strain isolation, OF samples at a concentration of 0.1 to 0.2% inoculum were cultured in BM or YCFA under anaerobic conditions. Changes in pH values and total bacterial cell numbers are shown in Fig. 1. The pH values rapidly decreased and cell numbers increased in YCFA compared to those observed in BM. However, lower pH values were observed in BM than in YCFA at 48 h (Fig. 1A). A marked increase in total cell numbers was obtained in both media at 48 h (Fig. 1B), whereas bifidobacteria were detected in 8 (53%) and 9 (60%) samples by qPCR after cultivation in BM and YCFA, respectively (Fig. 2A). Increased cell numbers of bifidobacteria were observed in 11 (73%) OF samples after cultivation in either BM or YCFA (Supplemental Table 1), indicating that there were viable bifidobacterial cells in these OF samples. The precultivated OF samples and those cultivated for 48 h were used to analyze the microbiotic profiles of the samples and to isolate *Bifidobacterium* strains.

### Microbiotic profiles of neonatal OF by 16S rRNA gene metagenomic analysis

**Figure 3:**
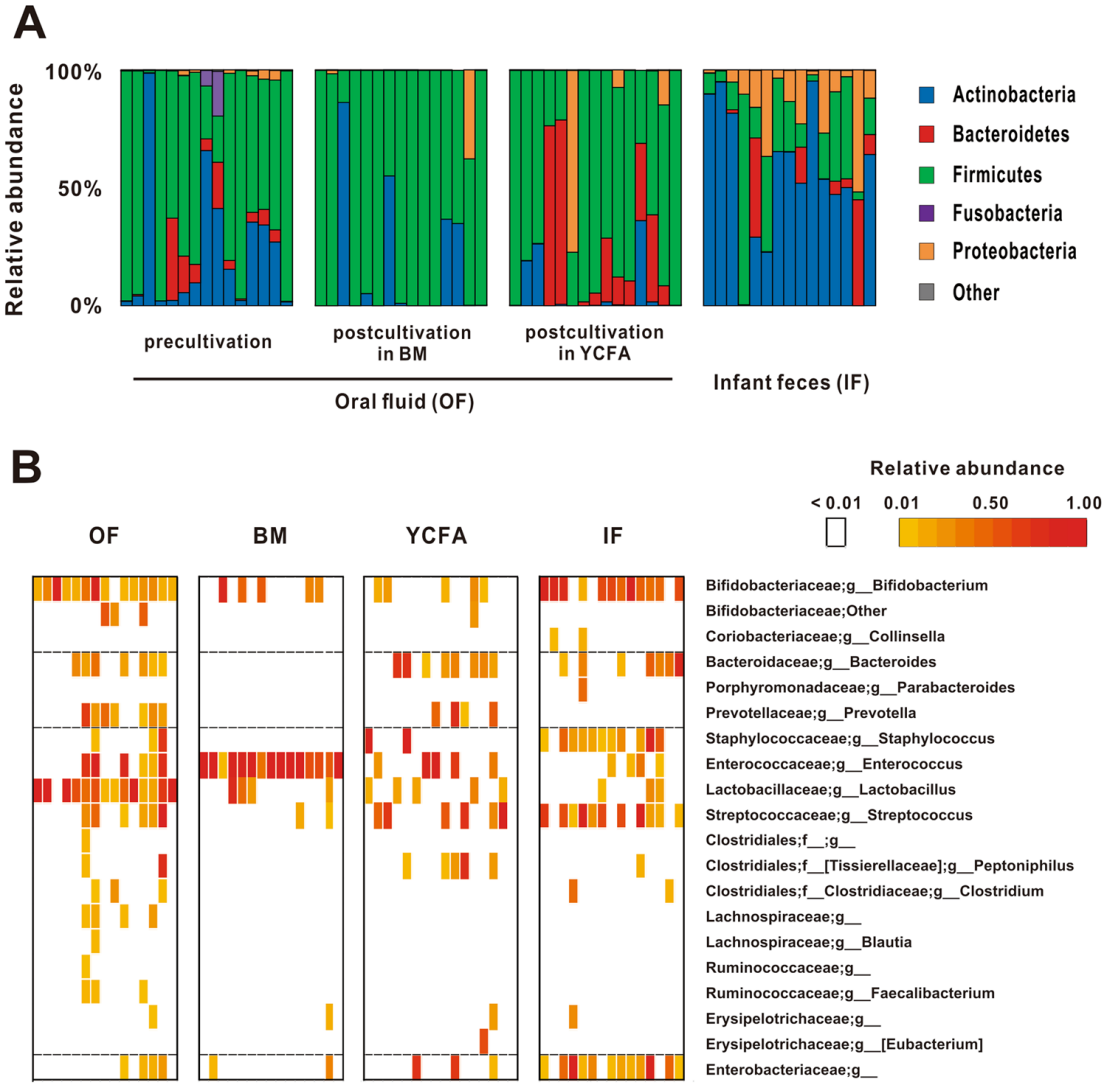
Microbiotic composition of OF and IF samples. Microbiotic compositions at the phylum (**A**) and genus levels (**B**) were assessed by V3-V4 region 16S rRNA gene sequencing. Each bar in each category indicates each ID in consecutive order from left to right. (**A**) The microbiotic profiles at the phylum level are depicted in the bar graph. (**B**) In the heatmap, the relative abundances of the top 20 genera are displayed in yellow to red for low to high abundance, respectively. Abbreviations: precultivated neonatal oral fluid (OF), postcultivated OF in BM (BM) or YCFA (YCFA) for 48 h and infant fecal (IF) samples.

Precultivated OF primarily harbored Firmicutes and Actinobacteria at the phylum level and Lactobacillaceae and Bifidobacteriaceae at the family level (Fig. 3A,B). Lactobacillaceae accounted for >50% of the families in 9 of the 15 OF samples (60%), whereas Bifidobacteriaceae accounted for >1% of the families in all OF samples (100%) (Fig. 3B), with sample ID03 containing the highest relative abundance of Bifidobacteriaceae (98.6%).

Although the prevalence of *Enterococcus* increased in all of the samples after cultivation in BM for 48 h, *Bifidobacterium* was the dominant taxon in 4 of the 15 samples (Fig. 3B). However, the dominant bacterial taxa detected after cultivation in YCFA were sample dependent: *Streptococcus* (6/15, 40%), *Enterococcus* (3/15, 20%), *Bacteroides* (2/15, 13%), *Eubacterium*, *Bifidobacterium*, Enterobacteriaceae and *Staphylococcus* (1/19, 7%) (Fig. 3B).

### Species-level quantification in OF and IF samples by qPCR

In OF samples, with or without cultivation for 48 h, *Bifidobacterium longum* subsp. *longum* (*B. longum*) was the most frequently detected species (detected in 53% of samples), followed by *B. catenulatum* group (47%), *B. breve* (40%), *B. adolescentis* (33%), *B. bifidum* (27%) and *B. longum* ssp. *infantis* (*B. infantis*, 7%). *B. dentium* and *B. animalis* ssp. *lactis* (*B. lactis*) were not detected (Fig. 2). In addition, *Bifidobacterium* was one of the predominant taxa in the IF samples (Fig. 3). The most prevalent *Bifidobacterium* species in the IF samples were *B. longum* (100%) and *B. breve* (100%), followed by *B. catenulatum* group (80%), *B. adolescentis* (80%) and others based on the results of the qPCR analysis (Fig. 2).

### Isolation of *Bifidobacterium* strains from OF, IF and MF samples

The OF (pre- and postcultivated for 48 h), IF and maternal fecal (MF) samples from 15 families were plated onto TOS propionate agar for colony formation under anaerobic conditions to isolate *Bifidobacterium* strains. We isolated a total of 888 colonies, and all colonies were subjected to random-amplified polymorphic DNA (RAPD) analysis to differentiate the same strains from the same samples. Furthermore, the strains selected as representatives were confirmed to be bifidobacteria using specific primers for *Bifidobacterium* (Supplemental Table 2). Consequently, a total of 82 strains were obtained and subjected to genome sequencing. Among them, similar strains from the same subject were excluded using the Markov Cluster Algorithm (MCL) based on the ORF content. Finally, 48 *Bifidobacterium* strains were obtained from the OF (18 strains from 10 subjects), IF (21 strains from 12 subjects) and MF (9 strains from 3 subjects) samples. *B. breve* (16) was the dominant strain, followed by *B. longum* (11), *B. pseudocatenulatum* (11), *B. bifidum* (4), *B. dentium* (3), *B. adolescentis* (2) and *B. infantis* (1) (Supplemental Table 3). The frequency at which *Bifidobacterium* was isolated from the OF samples increased in the postcultivated samples (67%) compared to that observed in the precultivated samples (27%).

### Identification of shared *Bifidobacterium* strains between OF, IF and MF samples

**Figure 4:**
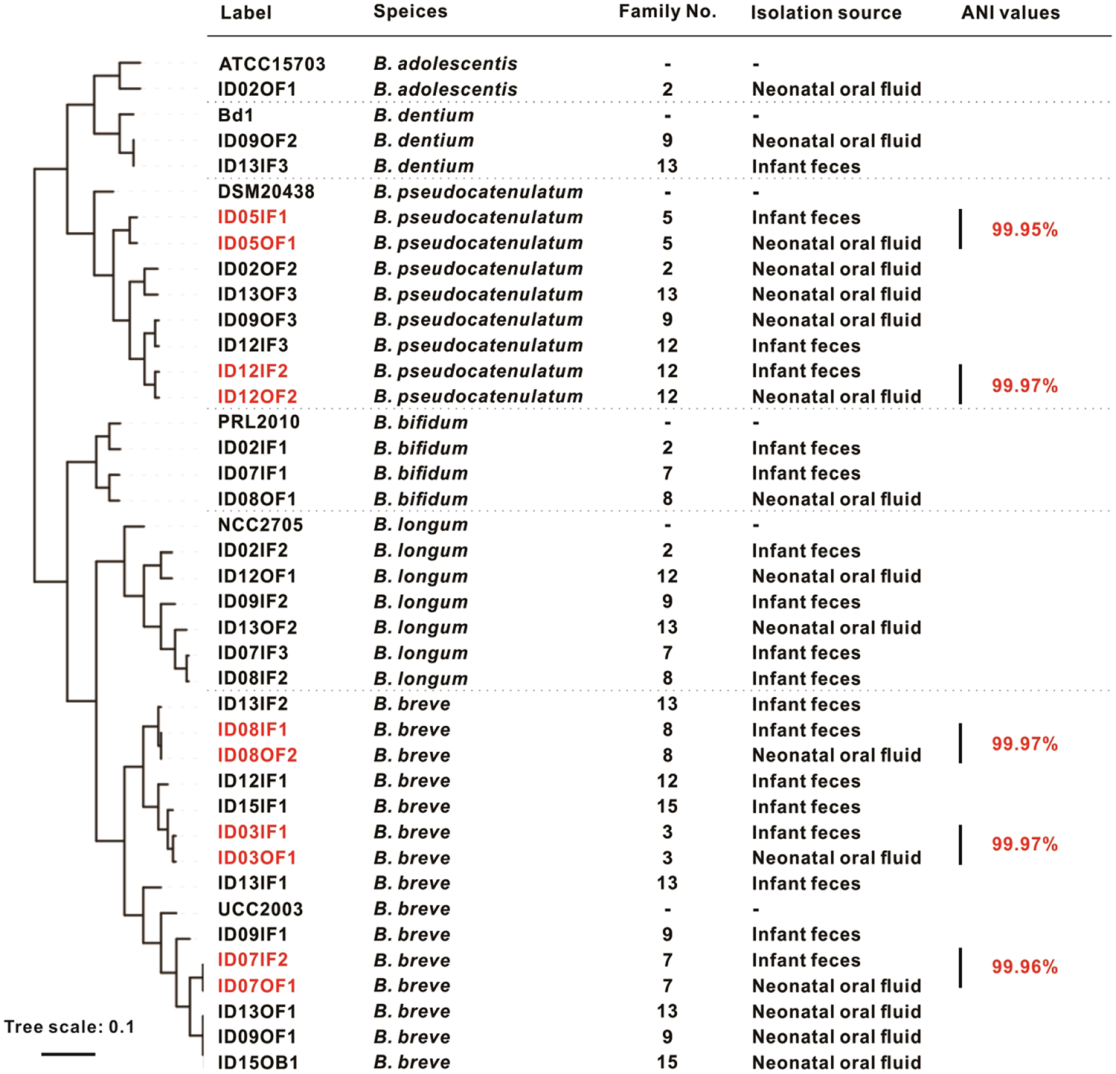
MCL clustering and genomic similarity analysis of the isolated strains from OF and IF samples. The left dendrogram shows the similarity of isolated strains from the OF and IF samples using MCL clustering based on the ORF content. The analyzed strains included those isolated from OF and IF samples and publicly available strains (*Bifidobacterium* species: *B. breve* UCC2003, *B. longum* NCC2705, *B. bifidum* PRL2010, *B. pseudocatenulatum* DSM20438, *B. dentium* Bd1 and *B. adolescentis* ATCC15703). Red labels indicate ANI values above 99.5% for paired strains.

*Bifidobacterium* strains were isolated from paired OF and IF samples for 9 of the 15 subjects. To find shared strains between OF and IF, the genomes of the 34 strains isolated from these 9 subjects were sequenced and clustered using MCL based on the ORF content. The results identified 8 strains that were shared between OF and IF pairs, including ID03, 05, 07, 08, 09, 12, 13 and 15. These strains were further assessed for genomic similarity. Five of the 8 paired strains were observed to possess an ANI of >99.5%, including *B. breve* in ID03, 07 and 08 and *B. pseudocatenulatum* in ID05 and 12 (Fig. 4).

Moreover, we also identified four strains that were shared between OF and MF pairs in two families. In particular, *B. pseudocatenulatum* from ID12 was observed to be shared by the MF-OF-IF samples (Supplemental Table 4).

## Discussion

Although *Bifidobacterium* is an early colonizer of the gastrointestinal tract of newborns and is one of the dominant bacterial genera in the gut of breastfeeding infants^3,4^, the sources of colonized bifidobacteria in the infant gut have yet to be completely elucidated. The goal of the present study was to assess the possibility that OF is one of the transmission routes for bifidobacteria to the infant gut immediately after birth. We confirmed the presence of viable bifidobacterial cells in OF samples, successfully isolated *Bifidobacterium* strains from OF and IF samples, and identified a number of strains with high DNA homology between the OF and IF samples based on homology analysis using genomic DNA sequences.

Bifidobacteria were previously detected in the vagina during pregnancy using culture-dependent^28,29^ and qPCR methods, suggesting that vaginal microbiota are one of the transmission sources of bifidobacteria. Interestingly, in this study, all of the neonatal OF samples were observed to contain Bifidobacteriaceae at relative abundance levels of 1% or greater by 16S rRNA gene sequence analysis (Fig. 3B). Similar to our findings, *Chu et al*. reported that *Bifidobacterium* was frequently detected in the neonatal oral cavity at birth^24^. These results suggest that bifidobacteria are present in neonatal OF at a high frequency.

Viable bacteria are required to act as a transmission source. However, our previous attempts to isolate *Bifidobacterium* strains from neonatal OF samples by direct plating on TOS propionate agar media proved challenging because of the low numbers of *Bifidobacterium* cells in samples and the small number of samples collected. To overcome these obstacles, we used a unique strategy that included cultivating OF in BM and YCFA media. BM has been suggested to allow for the selective growth of infant-type *Bifidobacterium* strains^3,30^, while YCFA is a medium that can support the growth of most gut bacterial species^27^, which could support the growth of both infant- and adult-type *Bifidobacterium* strains. The results indicated that viable bifidobacteria were present in OF samples, and this strategy enabled us to increase the number of bifidobacteria cells by 74% after cultivation in either BM or YCFA as well as to isolate *Bifidobacterium* strains from 10 postcultivated samples.

Based on an ANI of >99.5%, we identified paired *Bifidobacterium* strains in five OF-IF pairs. The ANI value, an index of the similarity of genomic coding regions, has been recently adapted for use in defining species of bacteria^28,29,31^. Furthermore, the use of ANI values is recognized as a successful tool to calculate the homology between bacterial strains. In this study, we used the presence of a species in the same MCL cluster based on the ORF content and an ANI value of >99.5% by OrthoANI analysis as criteria to define strains as being identical. Our results suggest that shared bifidobacterial strains were present in paired OF and IF samples. Although shared strains were detected in only one-third of the subjects, this result does not exclude the possible presence of the same strain in other subjects because all of the strains present in these samples may not have been isolated. Furthermore, we also identified shared strains in two MF-OF pairs. In particular, *B. pseudocatenulatum* from ID12 was observed to be shared by the MF-OF-IF samples (Supplemental Table 4), suggesting a direct transmission of bifidobacteria from the maternal gut to neonatal OF and the infant gut.

There are some limitations associated with this study, such as a limited sample size, sampling time and test period. Furthermore, we did not obtain amniotic fluid and vaginal fluid and collected only fecal samples from a portion of mothers during delivery. Despite these results, in general, we believe that the microbiota present in neonatal OF may be representative of all sources of maternal bacteria. However, analyses of amniotic and vaginal fluid and fecal samples may provide a more comprehensive understanding of the direct transmission of maternal bacteria to infants.

## Conclusion

The results of the present study provide evidence of the presence of viable bifidobacteria in neonatal OF at birth and the existence of the same strains in OF-IF pairs. To the best of our knowledge, our results indicate for the first time the possibility that OF is a mother-to-child transmission route for bifidobacteria. Further studies involving multisite samples from mothers will contribute to a more comprehensive understanding of direct maternal bacterial transmission.

## Methods

### Subjects and sample collection

This study was approved by the Ethics Committee of the School of Health and Sports Science, Juntendo University, and informed written consent was obtained from all of the mothers. All experiments were performed in accordance with relevant regulations. Fifteen healthy Japanese mother-infant pairs were enrolled in this study. All mothers were over 20 years old, and all newborns were full term and vaginally delivered. The exclusion criteria at recruitment included (1) pregnant women with metabolic disorders, such as gestational hypertension and gestational diabetes; (2) multiple pregnancies; (3) a cesarean section in the past; (4) the use of oral antimicrobials within 1 month of sampling; (5) a history of taking medication or having a serious diseases requiring medication; (6) infections, such as hepatitis B, HIV, and syphilis; and (7) a determination of unsuitability for this study by the investigator for other reasons. The detailed information is shown in Supplemental Table 5. Midwives collected OF samples by aspiration from the oral cavities of neonates immediately after birth in the delivery room. MF samples were collected from mothers who defecated during delivery (ID02, 06, 12 and 13). The OF and MF samples were stored at 4 °C under anaerobic conditions until cultivation, which occurred within 6 days of collection and was generally performed the day after collection. IF samples were also collected approximately one month after childbirth and were stored at −20 °C. All of the samples were transferred from the hospital to the laboratory at Morinaga Milk Industry and were stored at −80 °C (after the cultivation of OF) for further analysis.

### Breast milk

The BM used to culture the bacteria present in neonatal OF was obtained from BM stocks in the Neonatal Intensive Care Unit of Juntendo University. BM was centrifuged at 1,200 × g for 10 min at 4 °C to remove fat, and the supernatant was further centrifuged at 6,000 × g for 1 h at 4 °C to remove cells and other physiological debris. The supernatants from five BM samples were pooled and stored at −80 °C until their use for culturing.

### Cultivation

Neonatal OF samples were anaerobically cultivated in pooled BM or YCFA for 48 h using a pH-controlled multichannel fermenter (Bio Jr. 8; ABLE, Tokyo, Japan), as described by *Satoh et al*., with slight modifications^32^. Before inoculation, the BM or YCFA in the vessel was maintained at 37 °C with constant stirring at 87 rpm under anaerobic conditions, with the pH adjusted to 6.5 with NaOH. Next, neonatal OF was inoculated into BM or YCFA to a final concentration of 0.1 or 0.2% (v/v), as shown in Supplemental Table 1. After starting the culture, the pH was continuously monitored and adjusted to pH 5.5 with NaOH when the pH of the medium fell below 5.5. The culture broth was collected at 16, 24 and 48 h and was centrifuged at 10,000 × g for 10 min at 4 °C. The supernatant and bacterial pellet were collected and stored at −80 °C until further processing. No change in pH and total bacterial cell number was observed in BM or YCFA incubated without inoculation.

### Microbiotic profiling

The microbiotic profiles were assessed by sequencing the V3-V4 region of the 16S rRNA gene, as described previously^33^. Briefly, the bead-beating method was used to extract DNA from OF samples (20 μL), bacterial pellets from postcultivated broth (1.5 mL) and feces (20 mg). Subsequently, the DNA was PCR-amplified using the primers Tru357F (5′-CGCTCTTCCGATCTCTGTACGGRAGGCAGCAG-3′) and Tru806R (5′-CGCTCTTCCGATCTGACGGACTACHVGGGTWTCTAAT-3′). Next, the sequencing adapters for Illumina MiSeq were subsequently ligated to the amplicons, after which the samples were purified using a QIAquick 96 PCR Purification Kit (Qiagen, Valencia, CA, USA) and were then pooled. The libraries were sequenced on an Illumina MiSeq platform using a MiSeq v3 Reagent Kit 3 (Illumina Inc., San Diego, CA, USA). After removing sequences consistent with data from the Genome Reference Consortium human build 37 (GRCh37) and phiX reads from the raw Illumina paired-end reads, the sequences were analyzed using QIIME version 1.8.0. as previously described^33^. Potential chimeric sequences less than 150 bp in length with an average quality score of less than 25 or those lacking paired reads were also removed. Taxonomical classification was performed using the Greengenes reference database with a 97% threshold of OTU full-length sequences. The data at the phylum and genus levels are shown in Supplemental Tables 6 and 7, respectively. UniFrac distances were calculated using QIIME software.

### Real-time PCR for the quantitative determination of cell number

The quantitative real-time PCR (qPCR) conditions and the standard strains used for quantification were described previously^34^, and detailed information on the primer set used in this study is shown in Supplemental Table 8. The DNA samples were diluted 1- to 1,000-fold for qPCR. The total bacterial number was determined using universal primers for the 16S rRNA gene (Tru357F and Tru806R), and *Bifidobacterium longum* ssp. *longum* (*B. longum*) JCM1217^T^ was as the standard strain. In addition, the abundance levels of each *Bifidobacterium* species were determined using *B. adolescentis* JCM1275^T^, *B. animalis* ssp. *lactis* JCM1190^T^, *B. bifidum* JCM1255^T^, *B. breve* JCM1192^T^, *B. dentium* JCM1195^T^, *B. longum* JCM1217^T^ and *B. pseudocatenulatum* JCM1200^T^ as the standard strains for species-specific quantification. *B. longum* JCM1217^T^ was also used to estimate total cell numbers of bifidobacteria. The limit of detection (LOD) was defined as less than 10^4^ cells/mL or g, and data were calculated using LOD/2 as a complement value^35^.

### Isolation of bifidobacterial strains

For the isolation of bifidobacterial strains, pre- and postcultivated OF and IF samples were anaerobically plated on TOS propionate agar (Eiken Chemical, Tokyo, Japan) or modified agar, in which the carbon source was changed from galactooligosaccharides to 2′-fucosyllactose, D-(+)-cellobiose or L-(+)-arabinose supplemented with 50 mg/l mupirocin (Merck KGaA, Darmstadt, Germany) at 37 °C for 48 h, as described previously^36^. A maximum of 20 colonies per sample were randomly chosen from each sample and were cultivated in Difco Lactobacilli MRS (Becton Dickinson, NJ, USA) supplemented with 0.05% *L*-cysteine HCl (Kanto Chemical, Tokyo, Japan) at 37 °C for 16 h under anaerobic conditions. After pelleting of bacteria with centrifugation, the bacteria were incubated at 37 °C for overnight with enzymatic lysis buffer [20 mM Tris-HCl (pH8.0), 2 mM EDTA, 0.012%(v/v) Triton X-100, 20 mg/mL lysozyme and 500 U/mL mutanolysin]. Then, the cell suspension was treated with protease K and RNase, and the DNA from the bacteria was extracted using a DNeasy Blood & Tissue Kit as described previously^37^. The species were confirmed to be bifidobacteria by qPCR using a bifidobacteria-specific primer set. Subsequently, each bifidobacterial isolate was subjected to randomly amplified polymorphic DNA (RAPD)-PCR analysis for strain identification using the primers OPF-09 (5′-CCAAGCTTCC-3′) or OPF-11 (5′-TTGGTACCCC-3′). Isolates from the same sample with the same RAPD-PCR pattern were considered the same strain and were subjected to further genomic DNA sequencing, and detailed information on the isolates is shown in Supplemental Tables 2 and 3.

### Genome sequencing and sequence assembly

Genomic DNA was extracted from the subcultured strains in MRS as described above, and the libraries were prepared using a Nextera XT DNA Library Prep Kit (Illumina Inc.) according to the manufacturer’s instructions. Paired-end sequencing and quality trimming and *de novo* assembly of the raw reads were conducted using an Illumina MiSeq platform with a MiSeq v3 Reagent Kit and the CLC Genomics Workbench (v 8.0) software package (Qiagen, Valencia, CA, USA) with the default settings, except that the minimum contig length was set at 500 bp. Contigs composed of less than 100 reads were removed. Open reading frame (ORF) prediction and annotation were performed using the DDBJ Fast Annotation and Submission Tool (DFAST) with the default settings^38^.

### Genome analysis

Six bifidobacterial genomes (*B. adolescentis* ATCC15703, *B. bifidum* PRL2010, *B. breve* UCC2003, *B. dentium* Bd1, *B. longum* NCC2705 and *B. pseudocatenulatum* DSM20438) were retrieved from the NCBI public database for comparative purposes. Amino acid sequence comparisons were performed using an all-against-all, bidirectional BLAST alignment (cut-off: E-value 0.0001, with at least 50% identity across at least 50% of either protein sequence), followed by clustering into protein families using the MCL implemented in the mclblastline pipeline, v12-0678^36^. The isolates from the same sample in the same MCL cluster were removed from the further analysis.

For the candidate strains expected to have high similarity in the OF and IF pairs according to the results of the clustering analysis, their genome similarity was assessed by OrthoANI analysis with the ChunLab’s online ANI calculator (https://www.ezbiocloud.net/tools/ani)^39^. An ANI value of greater than 99.5% was the criterion used to identify identical strains^36^.

### Statistical analysis

The total cell numbers obtained during cultivation were analyzed by a one-way analysis of variance (ANOVA) followed by the Tukey-Kramer test and Welch’s *t*-test, respectively, using SPSS Statistics version 22.0 (IBM Corp., Armonk, NY, USA), with the significance level defined at p < 0.05.

## Supplementary information

Supplementary Dataset

## Data Availability

The data sets that support the findings of this study are available from the corresponding author, K.T., upon reasonable request. Furthermore, DNA sequences corresponding to 16S rRNA gene data were deposited in DDBJ under accession numbers DRA008239 (Supplemental Tables 6 and 7). Genome sequences of bifidobacteria were submitted to DDBJ (accession numbers are listed in Supplemental Table 3).

## References

[1] Gensollen T, Iyer SS, Kasper DL, Blumberg RS (2016). How colonization by microbiota in early life shapes the immune system. Science.

[2] Borre YE (2014). Microbiota and neurodevelopmental windows: implications for brain disorders. Trends Mol. Med..

[3] Wong CB, Sugahara H, Odamaki T, Xiao JZ (2018). Different physiological properties of human-residential and non-human-residential bifidobacteria in human health. Benef. Microbes.

[4] Milani C (2017). The First Microbial Colonizers of the Human Gut: Composition, Activities, and Health Implications of the Infant Gut Microbiota. Microbiol. Mol. Biol. Rev..

[5] Fukuda S (2011). Bifidobacteria can protect from enteropathogenic infection through production of acetate. Nature.

[6] Ruiz L, Delgado S, Ruas-Madiedo P, Sánchez B, Margolles A (2017). Bifidobacteria and Their Molecular Communication with the Immune System. Front. Microbiol..

[7] Nagpal R (2017). Evolution of gut Bifidobacterium population in healthy Japanese infants over the first three years of life: a quantitative assessment. Sci. Rep..

[8] Makino H (2015). Multilocus sequence typing of bifidobacterial strains from infant’s faeces and human milk: are bifidobacteria being sustainably shared during breastfeeding? *Benef*. Microbes.

[9] Makino H (2013). Mother-to-infant transmission of intestinal bifidobacterial strains has an impact on the early development of vaginally delivered infant’s microbiota. PLoS One.

[10] Milani C (2015). Exploring Vertical Transmission of Bifidobacteria from Mother to Child. Appl. Environ. Microbiol..

[11] Harmsen HJ (2000). Analysis of intestinal flora development in breast-fed and formula-fed infants by using molecular identification and detection methods. J. Pediatr. Gastroenterol. Nutr..

[12] Fanaro S, Chierici R, Guerrini P, Vigi V (2003). Intestinal microflora in early infancy: composition and development. Acta Paediatr. Suppl..

[13] Jost T, Lacroix C, Braegger CP, Chassard C (2012). New insights in gut microbiota establishment in healthy breast fed neonates. PLoS One.

[14] Lee ML (2006). The design and analysis of studies in premature infants using human donor milk or preterm formula as primary nutrition: a critique of Schanler et al. Breastfeed. Med..

[15] Martín V (2012). Sharing of bacterial strains between breast milk and infant feces. J. Hum. Lact..

[16] Gueimonde M, Laitinen K, Salminen S, Isolauri E (2007). Breast milk: a source of bifidobacteria for infant gut development and maturation?. Neonatology.

[17] Bernardi JR (2015). Cesarean delivery and metabolic risk factors in young adults: a Brazilian birth cohort study. Am. J. Clin. Nutr..

[18] Lee S-Y (2014). Additive effect between IL-13 polymorphism and cesarean section delivery/prenatal antibiotics use on atopic dermatitis: a birth cohort study (COCOA). PLoS One.

[19] Son G-H (2016). New insight into the analysis of amniotic fluid microflora using 16S rRNA gene sequencing. JMM Case Reports.

[20] Pelzer E, Gomez-Arango LF, Barrett HL, Nitert MD (2017). Review: Maternal health and the placental microbiome. Placenta.

[21] Satokari R, Grönroos T, Laitinen K, Salminen S, Isolauri E (2009). Bifidobacterium and Lactobacillus DNA in the human placenta. Lett. Appl. Microbiol..

[22] Perez-Muñoz ME, Arrieta M-C, Ramer-Tait AE, Walter J (2017). A critical assessment of the ‘sterile womb’ and ‘in utero colonization’ hypotheses: implications for research on the pioneer infant microbiome. Microbiome.

[23] Ross MG, Nijland MJ (1997). Fetal swallowing: relation to amniotic fluid regulation. Clin. Obstet. Gynecol..

[24] Chu DM (2017). Maturation of the infant microbiome community structure and function across multiple body sites and in relation to mode of delivery. Nat. Med..

[25] Li H (2018). The impacts of delivery mode on infant’s oral microflora. Sci. Rep..

[26] Torres-Alipi BI, Fragoso-Ramírez JA, Martínez-Limón AJ, Baptista-González HA (1990). [Bacterial colonization of the oral cavity in the newborn]. Bol. Med. Hosp. Infant. Mex..

[27] Browne HP (2016). Culturing of ‘unculturable’ human microbiota reveals novel taxa and extensive sporulation. Nature.

[28] Konstantinidis KT, Ramette A, Tiedje JM (2006). The bacterial species definition in the genomic era. Philos. Trans. R. Soc. Lond. B. Biol. Sci..

[29] Konstantinidis KT, Tiedje JM (2005). Genomic insights that advance the species definition for prokaryotes. Proc. Natl. Acad. Sci. USA.

[30] Minami J, Odamaki T, Hashikura N, Abe F, Xiao JZ (2016). Lysozyme in breast milk is a selection factor for bifidobacterial colonisation in the infant intestine. Benef. Microbes.

[31] Goris J (2007). DNA-DNA hybridization values and their relationship to whole-genome sequence similarities. Int. J. Syst. Evol. Microbiol..

[32] Satoh T (2013). *In vitro* comparative evaluation of the impact of lacto-N-biose I, a major building block of human milk oligosaccharides, on the fecal microbiota of infants. Anaerobe.

[33] Odamaki T (2016). Age-related changes in gut microbiota composition from newborn to centenarian: a cross-sectional study. BMC Microbiol..

[34] Kato K (2017). Age-Related Changes in the Composition of Gut Bifidobacterium Species. Curr. Microbiol..

[35] Chen H, Quandt SA, Grzywacz JG, Arcury TA (2013). A Bayesian Multiple Imputation Method for Handling Longitudinal Pesticide Data with Values below the Limit of Detection. Environmetrics.

[36] Odamaki T (2018). Genomic diversity and distribution of Bifidobacterium longum subsp. longum across the human lifespan. Sci. Rep..

[37] Odamaki T (2019). Impact of a bathing tradition on shared gut microbe among Japanese families. Sci. Rep..

[38] Tanizawa Y, Fujisawa T, Kaminuma E, Nakamura Y, Arita M (2016). DFAST and DAGA: web-based integrated genome annotation tools and resources. Biosci. microbiota, food Heal..

[39] Yoon SH, Ha Smin, Lim J, Kwon S, Chun J (2017). A large-scale evaluation of algorithms to calculate average nucleotide identity. Antonie van Leeuwenhoek, Int. J. Gen. Mol. Microbiol..

