# Supplementary material for: Neonatal oral fluid as a transmission route for bifidobacteria to the infant gut immediately after birth
Source: Sci Rep. 2019 Jun 18;9:8692. doi: 10.1038/s41598-019-45198-9 (PMC6582144; doi:10.1038/s41598-019-45198-9)
Supplement: Supplementary file 1 — Supplementary Dataset [file 41598_2019_45198_MOESM1_ESM.pdf]

Supplementary information

Title

Neonatal oral fluid as a transmission route for bifidobacteria to the infant gut immediately after birth

Author names

Kazuya Toda 1*, Ken Hisata 2, Takumi Satoh 3, Noriko Katsumata 1, Toshitaka Odamaki 1, Eri Mitsuyama 1, Takane Katayama 4, Tetsuya Kuhara 1, Kohzo Aisaka 5, Toshiaki Shimizu 2, Jin-zhong Xiao 1.

Affiliation

1: Next Generation Science Institute R&D Division, Morinaga Milk Industry Co., Ltd., Kanagawa 252-8583, Japan

2: Department of Pediatrics and Adolescent Medicine, Juntendo University Graduate School of Medicine, Tokyo 113-8421, Japan

3: Department of Molecular Microbiology, Tokyo University of Agriculture, Tokyo 183-0051, Japan

4: Division of Integrated Life Science, Graduate School of Biostudies, Kyoto University, Sakyo-ku, Kyoto 606-8502, Japan

5: Department of Obstetrics and Gynecology, Hamada Hospital, Tokyo 101-0062, Japan

Corresponding author

*Corresponding author: Tel.: +81-462-52-3067, Fax: +81-462-52-3077

E-mail addresses

Corresponding author: kazuya-toda983@morinagamilk.co.jp

The list of supplemental data

Supplemental Table 1. Cell numbers of total bacteria and bifidobacteria in OF samples.

Supplemental Table 2. Cluster analysis of RAPD pattern profiles.

Supplemental Table 3. Detailed information of isolated strains from OF, IF and MF samples.

Supplemental Table 4. ANI values for candidate strains.

Supplemental Table 5. Detailed information on the subjects.

Supplemental Table 6. Detailed results of 16S rRNA gene sequence analysis at the

phylum level.

Supplemental Table 7. Detailed results of 16S rRNA gene sequence analysis at the genus level.

Supplemental Table 8. List of primer sets.

Supplemental Table 1. Cell numbers of total bacteria and bifidobacteria in OF samples.

ID	OF									IF	
	Precultivation					Postcultivation					
	Inoculum		Inoculation ratio	Calculated value		BM		YCFA			
Target	T	B		T	B	T	B	T	B	T	B
1	6	4.8	0.20%	3.3	2.1	7.5		7.2		8.5	10.1
2	4.8		0.20%	2.1		7.3		7.1	6.8	9	10.3
3	5.9	6.4	0.10%	2.9	3.4	8.5	9	8.6	7.8	8	9
4	5.4		0.20%	2.7		8		9		7.6	
5	5.4		0.20%	2.7		7.4	6.1	8.9		8	8.9
6	5.6	4.5	0.10%	2.6	1.5	8.5		8.6		7.7	
7	6		0.10%	3		9.1	9.1	8.9	5.9	7.8	8.9
8	6.9	6.7	0.20%	4.2	4	8.6	6.8	8.1	5	8.8	10.1
9	6.7	4.2	0.20%	4	1.5	8.6		8.8	6.7	8.8	10
10	6.2	4.2	0.20%	3.5	1.5	8.3		8.5	5.4	9.1	10.5
11	6.4	4.3	0.10%	3.4	1.3	5.8		8.7		8.3	9.2
12	6.3	4.8	0.20%	3.6	2.1	9.2	9.1	9.2	8.8	8.8	9.8
13	4.9		0.20%	2.2		8.4	8.1	9.2	7.6	7.6	8.2
14	4.8		0.20%	2.1		8.2	4.2	9	5.4	8.1	
15	4.5		0.10%	1.5		8.2	5.5	8.2		8.5	9.6

The values indicate the cell numbers (Log/mL or g).

Target shown as: T, total cell numbers; or B, *Bifidobacterium* .

Supplemental Table 2. Cluster analysis of RAPD pattern profiles

Family ID	Isolation source	A carbon source in isolation medium	Total number	Strain number																				Numbers of band-types based on RAPD-PCR (conduction of genomic sequencing)	Labels
				1	2	3	4	5	6	7	8	9	10	11	12	13	14	15	16	17	18	19	20		
1	precultivated OF	Galactooligosaccharide	0																						
	postcultivated OF in BM	Galactooligosaccharide	0																						
	postcultivated OF in YCFA	Galactooligosaccharide	0																						
	Infant feces	Galactooligosaccharide	20	a†	a	a	a	a	a	a	a	a	a	a	a	a	a	a	a	a	a	a	a	1	a: ID01IF1(INF)
2	precultivated OF	Galactooligosaccharide	0																						
	postcultivated OF in BM	Galactooligosaccharide	0																						a,e: ID02OF1(ADO), b: ID02OF2(CAT), c,d: Enterococcus faecalis
	postcultivated OF in YCFA	Galactooligosaccharide	12	a†	b†	c$	b	a	b	b	a	b	b	a	b									2	
		L-(+)-arabinose	8	d$	e†	e	d	e	e	e	e													1	
	Infant feces	Galactooligosaccharide	20	a†	b†	a	a	c$	a	a	a	a	a	a	a	a	a	a	a	a	d†	a	a	3	b: ID02IF1(BIF), a,d: ID02IF2(LON), c: Enterococcus faecalis
	precultivated MF	Galactooligosaccharide	15	a†	a	a	a	a	a	a	a	b†	a	a	b	a	a	a						2	a,f: ID02MF1(ADO), b,e: ID02MF2(BIF), c: ID02MF3(LON), d: Enterococcus faecalis
	postcultivated MF in BM	Galactooligosaccharide	12	c†	c	c	d$	c	c	c	c	c	c	e†	c									2	
	postcultivated MF in YCFA	Galactooligosaccharide	12	f†	f	c†	f	f	f	c	f	c	c	f	f									2	
3	precultivated OF	Galactooligosaccharide	15	a†	a	a	a	a	a	a	a	a	a	a	a	a	a	a						1	
	postcultivated OF in BM	Galactooligosaccharide	12	b†	b	b	b	b	b	c$	b	b	b	c	b									1	a,b,d: ID03OF1(BRE), c: Enterococcus faecalis
	postcultivated OF in YCFA	Galactooligosaccharide	12	d†	d	d	d	d	d	d	d	d	d	d	d									1	
	Infant feces	Galactooligosaccharide	20	a†	a	a	a	a	a	a	a	a	a	a	a	a	a	a	a	a	a	a	a	1	a: ID03IF1(BRE)
4	precultivated OF	Galactooligosaccharide	0																						
	postcultivated OF in BM	Galactooligosaccharide	0																						
	postcultivated OF in YCFA	Galactooligosaccharide	0																						
	Infant feces	Galactooligosaccharide	0																						
5	precultivated OF	Galactooligosaccharide	0																						
	postcultivated OF in BM	Galactooligosaccharide	15	a†	a	a	a	a	a	a	a	a	a	a	a	a	a	a						1	b: ID05OF1(CAT)
	postcultivated OF in YCFA	Galactooligosaccharide	0																						
	Infant feces	Galactooligosaccharide	20	a†	a	a	a	a	a	a	a	a	a	a	a	a	a	a	a	a	a	a	a	1	a: ID05IF1(CAT)
6	precultivated OF	Galactooligosaccharide	0																						
	postcultivated OF in BM	Galactooligosaccharide	0																						
	postcultivated OF in YCFA	Galactooligosaccharide	0																						
	Infant feces	Galactooligosaccharide	0																						
7	precultivated OF	Galactooligosaccharide	0																						
	postcultivated OF in BM	Galactooligosaccharide	16	a†	a	a	a	a	a	a	a	a	a	a	a	a	a	a	a					1	
		L-(+)-arabinose	8	b†	c†	b	b	c	c	c	b													2	a,b,c,e: ID07OF1(BRE), d,f: Enterococcus faecalis
		2'-Fucosyllactose	15	b	c	c	c	c	c	d$	d	b	c	c	c	d	c	b							
	postcultivated OF in YCFA	Galactooligosaccharide	16	a	a	a	a	a	a	a	e	e	e	f$	e	g	g	e	a						
	Infant feces	Galactooligosaccharide	20	a†	a	a	a	a	a	a	a	a	a	a	a	a	a	a	a	a	a	a	b†	2	
		L-(+)-arabinose	8	c†	d†	e†	f†	d	d	d	e													4	c: ID07IF1(BIF), a,b: ID07IF2(BRE), d,e: ID07IF3(LON)
		2'-Fucosyllactose	8	c	d	d	d	d	d	c	d														
8	precultivated OF	Galactooligosaccharide	15	a†	a	a	b$	b	b	b	b	b	a	b	b	a	b	a						1	
	postcultivated OF in BM	Galactooligosaccharide	15	c†	c	c	c	c	c	c	c	c	c	c	c	c	c	c						1	a: ID08OF1(BIF), c,f: ID08OF2(BRE), b,d,e: Enterococcus faecalis
		L-(+)-arabinose	8	d$	d	d	d	e$	d	d	d														
	postcultivated OF in YCFA	Galactooligosaccharide	15	f†	f	f	f	f	f	f	f	f	f	f	f	f	f	f						1	
	Infant feces	Galactooligosaccharide	20	a†	a	a	a	a	a	a	b†	b	b	a	a	a	a	a	a	a	a	a	a	2	a,c: ID08IF1(BRE), b: ID08IF2(LON)
		L-(+)-arabinose	8	a	a	c†	c	a	a	a	b													1	
9	precultivated OF	Galactooligosaccharide	0																						
	postcultivated OF in BM	Galactooligosaccharide	15	a†	b$	a	a	a	a	a	b	a	a	a	a	b	a	a						1	a: ID09OF1(BRE), g: ID09OF2(DEN), c,e: ID09OF3(CAT), b,d,f: Enterococcus faecalis
		D-(+)-Cellobiose	8	c†	d$	c	c	c	c	c	c													1	
	postcultivated OF in YCFA	Galactooligosaccharide	15	e†	e	e	e	e	e	e	f$	e	e	e	f	e	e	e						1	
		L-(+)-arabinose	8	g†	g	g	g	g	g	g	g													1	
	Infant feces	Galactooligosaccharide	20	a†	a	a	b†	a	a	a	a	a	a	a	a	a	a	a	a	a	a	a	a	2	
		D-(+)-Cellobiose	8	c†	c	c	c	c	c	c	c													1	a: ID09IF1(BRE), b,c,d: ID09IF2(LON)
		L-(+)-arabinose	8	d†	d	d	d	d	d	d	d													1	
10	precultivated OF	Galactooligosaccharide	0																						
	postcultivated OF in BM	Galactooligosaccharide	0																						
	postcultivated OF in YCFA	Galactooligosaccharide	0																						
	Infant feces	Galactooligosaccharide	20	a†	a	a	a	a	a	a	a	a	a	a	a	a	a	a	a	a	b$	a	a	1	a: ID10IF1(BRE), b: Enterococcus faecalis
11	precultivated OF	Galactooligosaccharide	0																						
	postcultivated OF in BM	Galactooligosaccharide	0																						
	postcultivated OF in YCFA	Galactooligosaccharide	0																						
	Infant feces	Galactooligosaccharide	20	a†	a	a	a	a	a	a	a	a	a	a	a	a	a	a	a	a	a	a	a	1	a: ID11IF1(DEN)
12	precultivated OF	Galactooligosaccharide	1	a†																				1	
	postcultivated OF in BM	Galactooligosaccharide	16	b†	b	b	c$	b	c	b	b	b	b	b	b	b	c	b	b					1	a,d,f: ID12OF1(LON), b: ID12OF2(CAT), c,e: Enterococcus faecalis
		2'-Fucosyllactose	8	d†	d	d	e$	e	d	d	e													1	
	postcultivated OF in YCFA	Galactooligosaccharide	16	f†	f	f	f	f	f	f	f	f	f	f	f	f	f	f	f					1	
	Infant feces	Galactooligosaccharide	20	a†	a	a	a	a	a	a	a	a	a	a	a	a	a	a	a	a	a	a	a	1	
		D-(+)-Cellobiose	8	b†	b	b	c†	c	c	b	b													2	a: ID12IF1(BRE), b: ID12IF2(CAT), c: ID12IF3(CAT)
		L-(+)-arabinose	8	b	b	b	b	b	b	b	b														
	precultivated MF	Galactooligosaccharide	16	a†	b†	c$	c	a	c	a	a	a	c	c	c	a	a	c	a					2	
	postcultivated MF in BM	Galactooligosaccharide	16	d†	a	a	a	a	a	a	a	a	a	a	a	a	a	a	a					1	a,d,g: ID12MF1(LON), b,e: ID12MF2(CAT), f: ID12MF3(CAT), c :Enterococcus faecalis
		2'-Fucosyllactose	8	e†	e	f†	f	e	e	e	e													2	
	postcultivated MF in YCFA	Galactooligosaccharide	16	g†	g	g	g	g	g	g	g	g	g	g	g	g	g	g	g					1	
13	precultivated OF	Galactooligosaccharide	0																						
	postcultivated OF in BM	Galactooligosaccharide	16	a†	a	a	a	a	a	a	a	a	a	a	a	a	a	a	a					1	b: ID13OF1(BRE), a,d: ID13OF2(LON), e: ID13OF3(CAT), c: Enterococcus faecalis
		2'-Fucosyllactose	8	b†	c$	b	c	b	b	b	c													1	
	postcultivated OF in YCFA	Galactooligosaccharide	16	d†	e†	e	e	e	e	e	d	d	e	e	e	e	e	d	e					2	
	Infant feces	Galactooligosaccharide	20	a†	a	a	a	a	a	a	a	a	b$	a	a	a	a	a	a	a	a	a	a	1	
		2'-Fucosyllactose	8	c†	d$	c	d	c	c	d	c													1	c: ID13IF1(BRE), a,e: ID13IF2(BRE), f,g: ID13IF3(DEN), b,d: Enterococcus faecalis
		L-(+)-arabinose	8	e†	f†	f	f	e	e	g†	f													3	
		D-(+)-Cellobiose	16	e	e	e	e	e	e	e	e	e	e	e	e	e	e	e	e						
	precultivated MF	Galactooligosaccharide	16	a†	a	a	b†	a	b	b	b	b	b	a	a	b	b	b	b					2	
	postcultivated MF in BM	Galactooligosaccharide	16	c†	c	c	c	c	c	c	c	c	c	c	c	c	c	c	c					1	d: ID13MF1(BRE), a,c,e: ID13MF2(LON), b,f: ID13MF3(CAT)
		2'-Fucosyllactose	8	d†	d	d	d	d	d	d	d													1	
	postcultivated MF in YCFA	Galactooligosaccharide	16	e†	f†	e	f	f	f	e	f	f	f	e	f	f	f	e	f					2	
14	precultivated OF	Galactooligosaccharide	16	a†	a	a	a	a	a	a	a	a	b†	a	a	a	a	a	a					2	a,c: ID14OF1(LON), b: ID14OF2(LON), d: Enterococcus facalis
	postcultivated OF in BM	Galactooligosaccharide	16	c†	c	d$	d	d	d	d	d	d	d	d	d	d	d	d	d					1	
	postcultivated OF in YCFA	Galactooligosaccharide	16	a†	a	a	a	a	a	a	a	a	a	a	a	a	a	a	a						
	Infant feces	Galactooligosaccharide	20	a$	a	a	a	a	a	a	a	a	a	a	a	a	a	a	a	a	a	a	a		a: Enterococcus facalis
15	precultivated OF	Galactooligosaccharide	0																						
	postcultivated OF in BM	Galactooligosaccharide	16	a†	a	b†	a	a	a	a	a	a	a	a	a	a	a	a	a					2	a,b,c,d: ID15OF1(BRE)
		2'-Fucosyllactose	8	c†	c	c	c	d†	d	c	c													2	
	postcultivated OF in YCFA	Galactooligosaccharide	0																						
	Infant feces	Galactooligosaccharide	20	a	a†	a	a	a	a	a	a	a	a	a	a	a	a	a	a	a	a	a	a	1	a,b,c: ID15IF1(BRE)
		2'-Fucosyllactose	8	b†	b	b	b	b	b	b	c†													2	
Total	OF		381																					32	
	IF		356																					32	
	MF		151																					18	
	Total		888																					82	

*1 A dagger symbol (†) represented the genome-sequenced strain.

*2 A dollar sign ($) was the strain identified as *Enterococcus facalis* using PCR analysis.

Supplemental Table 3. Detailed information of isolated strains from OF, IF and MF samples.

Label	Family No.	Isolation Source	Species	Total Sequence Length (Mbp):	Number of Sequences:	Longest Sequences (bp):	N50 (bp):	Gap Ratio (%):	GC content (%):	Number of CDSs:	Average Protein Length:	Coding Ratio (%):	Number of rRNAs:	Number of tRNAs:	Number of CRISPRs:	Cultivation Medium	Carbon source in TOS Agar Plate	DDBJ accession number	Morinaga Culture Collection (MCC) number
ID01IF1	1	Infant feces	*B. infantis*	2.67	76	164760	94009	0.0075	59.8	2334	323.4	84.7	2	58	0	-	Galactooligosaccharide	BJIA01000001-BJIA01000076	MCC-01971
ID02IF1	2	Infant feces	*B. bifidum*	2.22	45	242612	118940	0.005	62.8	1868	337.9	85.2	1	55	1	-	Galactooligosaccharide	BJIB01000001-BJIB01000045	MCC-01946
ID02IF2	2	Infant feces	*B. longum*	2.47	58	233153	124210	0.0042	59.7	2127	333.9	86.2	2	76	2	-	Galactooligosaccharide	BJIC01000001-BJIC01000058	MCC-01972
ID02MF1	2	Maternal feces	*B. adolescentis*	2.29	23	743896	462236	0.0011	59.3	1943	342.3	87.3	2	59	1	-	Galactooligosaccharide	BJID01000001-BJID01000035	MCC-01943
ID02MF2	2	Maternal feces	*B. bifidum*	2.22	47	232728	118304	0.002	62.8	1868	336.4	84.8	2	55	1	-	Galactooligosaccharide	BJIE01000001-BJIE01000023	MCC-01947
ID02MF3	2	Maternal feces	*B. longum*	2.45	66	263956	115024	0.0027	59.9	2080	336.8	86	2	77	2	YCFA	Galactooligosaccharide	BJIF01000001-BJIF01000066	MCC-01975
ID02OF1	2	Neonatal oral fluid	*B. adolescentis*	2.28	20	1430044	1430044	0.0028	59.3	1952	340.6	87.5	2	59	1	YCFA	Galactooligosaccharide	BJIG01000001-BJIG01000020	MCC-01941
ID02OF2	2	Neonatal oral fluid	*B. pseudocatenulatum*	2.38	20	757568	505692	0.0011	56.5	1986	344.4	86.3	3	57	1	YCFA	Galactooligosaccharide	BJIH01000001-BJIH01000020	MCC-01992
ID03IF1	3	Infant feces	*B. breve*	2.26	101	255869	212069	0.0006	58.7	1923	333.8	85.3	1	55	0	-	Galactooligosaccharide	BJII01000001-BJII01000527	MCC-01950
ID03OF1	3	Neonatal oral fluid	*B. breve*	2.23	28	335081	219798	0.0014	58.7	1928	333.1	86.3	1	55	0	-	Galactooligosaccharide	BJIJ01000001-BJIJ01000028	MCC-01951
ID05IF1	5	Infant feces	*B. pseudocatenulatum*	2.25	257	81150	15424	0.0001	56.2	1712	338.2	77.2	1	48	5	-	Galactooligosaccharide	BJIK01000001-BJIK01000257	MCC-01994
ID05OF1	5	Neonatal oral fluid	*B. pseudocatenulatum*	2.25	96	124875	42605	0.0002	56.2	1800	352.4	84.4	2	51	4	BM	Galactooligosaccharide	BJIL01000001-BJIL01000096	MCC-01993
ID07IF1	7	Infant feces	*B. bifidum*	2.25	81	304615	178434	0.0015	62.8	1846	344.3	84.9	2	56	0	-	2'-Fucosyllactose	BJIM01000001-BJIM01000081	MCC-02030
ID07IF2	7	Infant feces	*B. breve*	2.34	43	316196	127027	0.0035	58.8	2021	333.4	86.3	2	55	1	-	Galactooligosaccharide	BJIN01000001-BJIN01000043	MCC-01954
ID07IF3	7	Infant feces	*B. longum*	2.33	37	410666	146688	0.002	59.9	1922	351.9	87	2	54	1	-	Galactooligosaccharide	BJIO01000001-BJIO01000037	MCC-01976
ID07OF1	7	Neonatal oral fluid	*B. breve*	2.34	43	316196	127027	0.0035	58.8	2021	333.4	86.3	2	55	1	BM	Galactooligosaccharide	BJIP01000001-BJIP01000028	MCC-01953
ID08IF1	8	Infant feces	*B. breve*	2.43	24	565698	311771	0.0116	58.5	2143	328.7	86.8	2	55	1	-	Galactooligosaccharide	BJIQ01000001-BJIQ01000024	MCC-01957
ID08IF2	8	Infant feces	*B. longum*	2.33	39	410576	146434	0.0075	59.9	1918	352.7	86.9	2	53	1	-	Galactooligosaccharide	BJIR01000001-BJIR01000039	MCC-01977
ID08OF1	8	Neonatal oral fluid	*B. bifidum*	2.21	57	215216	96233	0.0016	62.8	1835	342.2	85.2	2	54	0	-	Galactooligosaccharide	BJIS01000001-BJIS01000057	MCC-01958
ID08OF2	8	Neonatal oral fluid	*B. breve*	2.45	39	594361	283907	0.004	58.4	2148	328.2	86.3	2	55	1	YCFA	Galactooligosaccharide	BJIT01000001-BJIT01000039	MCC-01959
ID09IF1	9	Infant feces	*B. breve*	2.33	39	285123	108385	0.0018	58.8	1951	342.5	86.2	2	54	1	-	Galactooligosaccharide	BJIU01000001-BJIU01000039	MCC-01963
ID09IF2	9	Infant feces	*B. longum*	2.35	170	171627	28382	0.006	60.1	1884	342.4	82.3	2	53	4	-	Galactooligosaccharide	BJIV01000001-BJIV01000170	MCC-01978
ID09OF1	9	Neonatal oral fluid	*B. breve*	2.26	22	405061	162736	0.0042	58.9	1969	331.9	86.7	1	54	0	BM	Galactooligosaccharide	BJIW01000001-BJIW01000022	MCC-01961
ID09OF2	9	Neonatal oral fluid	*B. dentium*	2.6	67	444170	176860	0.0085	58.6	2183	347.7	87.5	3	55	1	YCFA	L-(+)-arabinose	BJIX01000001-BJIX01000067	MCC-02031
ID09OF3	9	Neonatal oral fluid	*B. pseudocatenulatum*	2.32	22	890993	287645	0.0008	56.9	1890	354.4	86.8	3	54	2	YCFA	Galactooligosaccharide	BJIY01000001-BJIY01000022	MCC-01995
ID10IF1	10	Infant feces	*B. breve*	2.22	33	605039	264331	0.0033	58.7	1903	334.4	85.9	2	54	0	-	Galactooligosaccharide	BJIZ01000001-BJIZ01000033	MCC-01964
ID11IF1	10	Infant feces	*B. dentium*	2.6	26	382911	305666	0.0068	58.5	2157	352.3	87.6	3	55	4	-	Galactooligosaccharide	BJJA01000001-BJJA01000026	MCC-01970
ID12IF1	12	Infant feces	*B. breve*	2.44	43	562774	134058	0.0016	59.2	2174	322.6	86.1	2	75	1	-	Galactooligosaccharide	BJJB01000001-BJJB01000043	MCC-01966
ID12IF2	12	Infant feces	*B. pseudocatenulatum*	2.27	46	424289	353901	0.0008	56.7	1844	355.4	86.6	1	54	0	-	D-(+)-Cellobiose	BJJC01000001-BJJC01000046	MCC-02032
ID12IF3	12	Infant feces	*B. pseudocatenulatum*	2.33	73	335434	115514	0.0052	56.9	1880	354.4	85.8	3	54	2	-	D-(+)-Cellobiose	BJJD01000001-BJJD01000073	MCC-02033
ID12MF1	12	Maternal feces	*B. longum*	2.53	76	167558	67927	0.0014	59.9	2134	341.5	86.3	2	79	3	YCFA	Galactooligosaccharide	BJJE01000001-BJJE01000076	MCC-01983
ID12MF2	12	Maternal feces	*B. pseudocatenulatum*	2.29	83	395652	99812	0.0007	56.7	1844	356	86.1	2	54	0	BM	2'-Fucosyllactose	BJJF01000001-BJJF01000083	MCC-02034
ID12MF3	12	Maternal feces	*B. pseudocatenulatum*	2.28	77	353658	175534	0.0014	56.7	1844	355.7	86.3	2	54	0	BM	2'-Fucosyllactose	BJJG01000001-BJJG01000077	MCC-02035
ID12OF1	12	Neonatal oral fluid	*B. longum*	2.54	73	185555	68250	0.0028	59.9	2149	339.1	86.2	2	79	3	-	Galactooligosaccharide	BJJH01000001-BJJH01000073	MCC-01979
ID12OF2	12	Neonatal oral fluid	*B. pseudocatenulatum*	2.27	36	293948	134272	0.0011	56.7	1844	357.7	87.2	2	54	0	BM	Galactooligosaccharide	BJJI01000001-BJJI01000036	MCC-01996
ID13IF1	13	Infant feces	*B. breve*	2.47	28	323794	177651	0.0071	58.6	2171	326.2	86.1	2	77	2	-	Galactooligosaccharide	BJJJ01000001-BJJJ01000028	MCC-01967
ID13IF2	13	Infant feces	*B. breve*	2.44	274	131186	27827	0.0099	58.5	2022	321.4	79.8	2	54	2	-	L-(+)-arabinose	BJJK01000001-BJJK01000274	MCC-02036
ID13IF3	13	Infant feces	*B. dentium*	2.6	67	322579	165925	0.0081	58.6	2182	348.1	87.6	3	55	1	-	L-(+)-arabinose	BJJL01000001-BJJL01000022	MCC-02037
ID13MF1	13	Maternal feces	*B. breve*	2.36	219	415273	382788	0.0058	58.7	1979	334.8	84.4	2	54	0	BM	2'-Fucosyllactose	BJJM01000001-BJJM01000219	MCC-02038
ID13MF2	13	Maternal feces	*B. longum*	2.38	36	266064	124325	0.0031	59.8	1995	346.1	86.9	2	60	0	BM	Galactooligosaccharide	BJJN01000001-BJJN01000036	MCC-01986
ID13MF3	13	Maternal feces	*B. pseudocatenulatum*	2.23	22	752541	413420	0.0045	56.2	1823	355.7	87.2	2	55	1	YCFA	Galactooligosaccharide	BJJO01000001-BJJO01000022	MCC-01988
ID13OF1	13	Neonatal oral fluid	*B. breve*	2.28	74	338849	218389	0.0045	58.9	1980	331.3	86.1	1	54	0	BM	2'-Fucosyllactose	BJJP01000001-BJJP01000074	MCC-02039
ID13OF2	13	Neonatal oral fluid	*B. longum*	2.38	43	248213	109588	0.0029	59.8	1992	345	86.5	2	58	0	BM	Galactooligosaccharide	BJJQ01000001-BJJQ01000043	MCC-01984
ID13OF3	13	Neonatal oral fluid	*B. pseudocatenulatum*	2.23	29	379214	143234	0.0004	56.2	1812	356.6	86.9	2	55	1	YCFA	Galactooligosaccharide	BJJR01000001-BJJR01000029	MCC-01998
ID14OF1	14	Neonatal oral fluid	*B. longum*	2.46	46	312578	130152	0.0011	59.8	2153	328.9	86.3	2	56	3	-	Galactooligosaccharide	BJJS01000001-BJJS01000046	MCC-01989
ID14OF2	14	Neonatal oral fluid	*B. longum*	2.38	31	270543	164562	0.0061	59.8	2003	344.5	86.8	2	58	0	-	Galactooligosaccharide	BJJT01000001-BJJT01000031	MCC-01990
ID15IF1	15	Infant feces	*B. breve*	2.2	26	465231	134114	0.0019	58.6	1900	333.5	86.2	1	55	0	-	Galactooligosaccharide	BJJU01000001-BJJU01000026	MCC-01969
ID15OF1	15	Neonatal oral fluid	*B. breve*	2.26	20	525267	389540	0.0016	58.9	1965	332.8	86.8	2	54	0	BM	Galactooligosaccharide	BJJV01000001-BJJV01000020	MCC-01968

Supplemental Table 4. ANI values for candidate strains.

Candidate No.	Label	Family No.	Isolation Source	Species	ANI Values
1	ID03IF1<br>ID03OF1	3	Infant feces<br>Neonatal oral fluid	*B. breve*	99.97%
2	ID05IF1<br>ID05OF1	5	Infant feces<br>Neonatal oral fluid	*B. pseudocatenulatum*	99.95%
3	ID07IF2<br>ID07OF1	7	Infant feces<br>Neonatal oral fluid	*B. breve*	99.96%
4	ID08IF1<br>ID08OF2	8	Infant feces<br>Neonatal oral fluid	*B. breve*	99.97%
5	ID09IF1<br>ID09OF1	9	Infant feces<br>Neonatal oral fluid	*B. breve*	98.19%
6	ID12IF2<br>ID12OF2	12	Infant feces<br>Neonatal oral fluid	*B. pseudocatenulatum*	99.97%
7	ID13IF1<br>ID13OF1	13	Infant feces<br>Neonatal oral fluid	*B. breve*	98.39%
8	ID15IF1<br>ID15OB1	15	Infant feces<br>Neonatal oral fluid	*B. breve*	98.78%
9	ID02MF1<br>ID02OF1	2	Maternal feces<br>Neonatal oral fluid	*B. adolescentis*	98.75%
10	ID12MF1<br>ID12OF1	12	Maternal feces<br>Neonatal oral fluid	*B. longum*	99.95%
11	ID12MF2<br>ID12OF2	12	Maternal feces<br>Neonatal oral fluid	*B. pseudocatenulatum*	99.96%
12	ID13MF3<br>ID13OF3	13	Maternal feces<br>Neonatal oral fluid	*B. pseudocatenulatum*	99.97%
13	ID13MF2<br>ID13OF2	13	Maternal feces<br>Neonatal oral fluid	*B. longum*	99.99%

Supplemental Table 5. Detailed information on the subjects.

Subject ID	Day		Information about infants						Information about mothers								
	Birthday	Hospital visiting day for 1 month check up	Infant gender	Body weight (g)		Nutritional status		Medication from birth to first 3-4 weeks of life	Gestational age at birth (weeks)	Blood group	Maternal age at birth (year-old)	Maternal defecation during delivery	Habitually intake of milk products	Habitually intake of probiotics	Use of antimicrobials at birth	Drug use during pregnancy	Notes
				At birth	At first 3-4 weeks of life	Breastfeeding (%)	Formula feeding (%)										
1	10-Jun-2017	18-Jul-2017	female	3450	3750	75	25	-	40	O	36	-	+	+	-	Gastrointestinal agents, Laxatives, Antihistamine, Antiallergic agents, Dermatologic preparation	Meconium staining
2	12-Jun-2017	18-Jul-2017	female	2660	3492	50	50	-	38	A	38	+	-	+	-	Tocolytic agent, Thyroid hormone preparation, Laxatives	Female infertility, multifetal pregnancy reduction
3	19-Jun-2017	18-Jul-2017	female	3510	4858	70	30	-	39	unknown	31	-	+	+	-	-	
4	19-Jun-2017	20-Jul-2017	male	3475	4396	100	0	-	38	A	35	-	+	-	+	Antihistamine, Antipyretic-analgesics	BMI16.3, GBS(2+)
5	15-Jul-2017	17-Aug-2017	female	3325	4282	80	20	-	39	A	34	-	+	+	-	Tocolytic agent	Pyelonephritis
6	8-Aug-2017	12-Sep-2017	female	2900	3844	70	30	-	41	B	32	+	+	+	-	Tocolytic agent, Antianemic agent	
7	9-Aug-2017	13-Aug-2017	male	4120	5195	100	0	-	40	O	36	-	-	-	-	-	
8	14-Aug-2017	12-Sep-2017	male	3110	4816	100	0	-	39	A	28	-	+	+	-	Laxatives	
9	15-Aug-2017	22-Sep-2017	female	2760	3484	100	0	-	39	A	28	-	+	+	-	Antianemic agent, Laxatives	
10	22-Aug-2017	25-Aug-2017	female	3280	4142	100	0	-	39	B	38	-	+	+	-	-	
11	24-Aug-2017	23-Sep-2017	female	2780	4052	100	0	-	38	B	31	-	-	-	-	Tocolytic agent, Antianemic agent, Gastric mucosal protection agent	
12	30-Aug-2017	27-Sep-2017	female	3135	4398	100	0	-	39	A	37	+	-	-	+	Laxatives, Antipyretic-analgesics, Prophylactic for oral bacterial infection	
13	5-Sep-2017	8-Sep-2017	male	3595	4254	33	67	-	41	A	28	+	+	+	+	Antianemic agent,Antianemic agent, Gastric mucosal protection agent, Laxatives	
14	6-Sep-2017	3-Oct-2017	female	2925	3970	90	10	-	40	O	32	-	+	-	-	-	
15	11-Sep-2017	10-Oct-2017	male	3480	4664	100	0	-	38	O	31	-	-	+	-	Tocolytic agent, Laxatives, Antianemic agent, Gastric mucosal protection agent, Antitussive drugs	

Supplemental Table 6. Detailed results of 16S rRNA gene sequence analysis at the phylum levels.

Type	precultivated OF														
Subject ID	ID01	ID02	ID03	ID04	ID05	ID06	ID07	ID08	ID09	ID10	ID11	ID12	ID13	ID14	ID15
k Bacteria;p Acidobacteria	0.000	0.000	0.000	0.000	0.000	0.000	0.000	0.000	0.000	0.000	0.000	0.000	0.000	0.000	0.000
k Bacteria;p Actinobacteria	0.019	0.041	0.986	0.020	0.024	0.056	0.098	0.659	0.413	0.155	0.024	0.354	0.344	0.270	0.018
k Bacteria;p Bacteroidetes	0.002	0.007	0.002	0.001	0.348	0.154	0.077	0.050	0.197	0.037	0.005	0.041	0.066	0.053	0.002
k Bacteria;p Chloroflexi	0.000	0.000	0.000	0.000	0.000	0.000	0.000	0.000	0.000	0.000	0.000	0.000	0.000	0.000	0.000
k Bacteria;p Cyanobacteria	0.000	0.000	0.000	0.000	0.000	0.000	0.000	0.000	0.000	0.000	0.000	0.000	0.000	0.000	0.000
k Bacteria;p Deferribacteres	0.000	0.000	0.000	0.000	0.000	0.000	0.000	0.000	0.000	0.000	0.000	0.000	0.000	0.000	0.000
k Bacteria;p Firmicutes	0.976	0.949	0.011	0.978	0.626	0.767	0.816	0.224	0.194	0.795	0.970	0.581	0.552	0.634	0.978
k Bacteria;p Fusobacteria	0.000	0.000	0.000	0.000	0.000	0.002	0.000	0.066	0.190	0.000	0.000	0.000	0.000	0.000	0.000
k Bacteria;p Proteobacteria	0.001	0.003	0.001	0.001	0.000	0.021	0.008	0.001	0.000	0.013	0.001	0.020	0.038	0.042	0.002
k Bacteria;p Synergistetes	0.000	0.000	0.000	0.000	0.000	0.000	0.000	0.000	0.000	0.000	0.000	0.000	0.000	0.000	0.000
k Bacteria;p Tenericutes	0.003	0.000	0.000	0.000	0.002	0.000	0.000	0.000	0.006	0.000	0.000	0.003	0.000	0.000	0.000
k Bacteria;p Verrucomicrobia	0.000	0.000	0.000	0.000	0.000	0.000	0.000	0.000	0.000	0.000	0.000	0.000	0.000	0.000	0.000
Unassigned;Other	0.000	0.000	0.000	0.000	0.000	0.001	0.000	0.000	0.000	0.000	0.000	0.000	0.000	0.000	0.000

Type	postcultivated OF in BM for 48h														
Subject ID	ID01	ID02	ID03	ID04	ID05	ID06	ID07	ID08	ID09	ID10	ID11	ID12	ID13	ID14	ID15
k Bacteria;p Acidobacteria	0.000	0.000	0.000	0.000	0.000	0.000	0.000	0.000	0.000	0.000	0.000	0.000	0.000	0.000	0.000
k Bacteria;p Actinobacteria	0.000	0.001	0.863	0.001	0.052	0.000	0.550	0.010	0.000	0.000	0.000	0.367	0.349	0.000	0.002
k Bacteria;p Bacteroidetes	0.000	0.000	0.000	0.000	0.000	0.000	0.000	0.000	0.000	0.000	0.000	0.000	0.000	0.003	0.000
k Bacteria;p Chloroflexi	0.000	0.000	0.000	0.000	0.000	0.000	0.000	0.000	0.000	0.000	0.000	0.000	0.000	0.000	0.000
k Bacteria;p Cyanobacteria	0.000	0.000	0.000	0.000	0.000	0.000	0.000	0.000	0.000	0.000	0.000	0.000	0.000	0.000	0.000
k Bacteria;p Deferribacteres	0.000	0.000	0.000	0.000	0.000	0.000	0.000	0.000	0.000	0.000	0.000	0.000	0.000	0.000	0.000
k Bacteria;p Firmicutes	0.999	0.984	0.137	0.999	0.946	0.998	0.449	0.990	0.999	1.000	0.998	0.632	0.651	0.620	0.997
k Bacteria;p Fusobacteria	0.000	0.000	0.000	0.000	0.000	0.000	0.000	0.000	0.000	0.000	0.000	0.000	0.000	0.000	0.000
k Bacteria;p Proteobacteria	0.000	0.015	0.000	0.000	0.002	0.002	0.000	0.000	0.000	0.000	0.002	0.000	0.000	0.377	0.000
k Bacteria;p Synergistetes	0.000	0.000	0.000	0.000	0.000	0.000	0.000	0.000	0.000	0.000	0.000	0.000	0.000	0.000	0.000
k Bacteria;p Tenericutes	0.000	0.000	0.000	0.000	0.000	0.000	0.000	0.000	0.000	0.000	0.000	0.000	0.000	0.000	0.000
k Bacteria;p Verrucomicrobia	0.000	0.000	0.000	0.000	0.000	0.000	0.000	0.000	0.000	0.000	0.000	0.000	0.000	0.000	0.000
Unassigned;Other	0.000	0.000	0.000	0.000	0.000	0.000	0.000	0.000	0.000	0.000	0.000	0.000	0.000	0.000	0.000

Type	postcultivated OF in YCFA for 48h														
Subject ID	ID01	ID02	ID03	ID04	ID05	ID06	ID07	ID08	ID09	ID10	ID11	ID12	ID13	ID14	ID15
k Bacteria;p Acidobacteria	0.000	0.000	0.000	0.000	0.000	0.000	0.000	0.000	0.000	0.000	0.000	0.000	0.000	0.000	0.000
k Bacteria;p Actinobacteria	0.001	0.190	0.262	0.001	0.007	0.001	0.001	0.002	0.017	0.004	0.003	0.362	0.017	0.002	0.002
k Bacteria;p Bacteroidetes	0.002	0.002	0.002	0.763	0.782	0.000	0.016	0.051	0.270	0.119	0.102	0.328	0.369	0.084	0.001
k Bacteria;p Chloroflexi	0.000	0.000	0.000	0.000	0.000	0.000	0.000	0.000	0.000	0.000	0.000	0.000	0.000	0.000	0.000
k Bacteria;p Cyanobacteria	0.000	0.000	0.000	0.000	0.000	0.000	0.000	0.000	0.000	0.000	0.000	0.000	0.000	0.000	0.000
k Bacteria;p Deferribacteres	0.000	0.000	0.000	0.000	0.000	0.000	0.000	0.000	0.000	0.000	0.000	0.000	0.000	0.000	0.000
k Bacteria;p Firmicutes	0.997	0.808	0.735	0.236	0.210	0.225	0.982	0.946	0.712	0.804	0.895	0.310	0.611	0.767	0.997
k Bacteria;p Fusobacteria	0.000	0.000	0.000	0.000	0.000	0.000	0.000	0.000	0.000	0.000	0.000	0.000	0.000	0.000	0.000
k Bacteria;p Proteobacteria	0.000	0.000	0.000	0.000	0.000	0.773	0.000	0.000	0.000	0.073	0.000	0.000	0.003	0.147	0.000
k Bacteria;p Synergistetes	0.000	0.000	0.000	0.000	0.000	0.000	0.000	0.000	0.000	0.000	0.000	0.000	0.000	0.000	0.000
k Bacteria;p Tenericutes	0.000	0.000	0.000	0.000	0.000	0.000	0.000	0.000	0.000	0.000	0.000	0.000	0.000	0.000	0.000
k Bacteria;p Verrucomicrobia	0.000	0.000	0.000	0.000	0.000	0.000	0.000	0.000	0.000	0.000	0.000	0.000	0.000	0.000	0.000
Unassigned;Other	0.000	0.000	0.000	0.000	0.000	0.000	0.001	0.000	0.000	0.000	0.000	0.000	0.000	0.000	0.000

Type	IF														
Subject ID	ID01	ID02	ID03	ID04	ID05	ID06	ID07	ID08	ID09	ID10	ID11	ID12	ID13	ID14	ID15
k Bacteria;p Acidobacteria	0.000	0.000	0.000	0.000	0.000	0.000	0.000	0.000	0.000	0.000	0.000	0.000	0.000	0.000	0.000
k Bacteria;p Actinobacteria	0.898	0.949	0.816	0.003	0.291	0.227	0.654	0.653	0.520	0.953	0.537	0.474	0.501	0.001	0.641
k Bacteria;p Bacteroidetes	0.001	0.003	0.015	0.001	0.422	0.002	0.000	0.001	0.153	0.001	0.001	0.054	0.039	0.449	0.085
k Bacteria;p Chloroflexi	0.000	0.000	0.000	0.000	0.000	0.000	0.000	0.000	0.000	0.000	0.000	0.000	0.000	0.000	0.000
k Bacteria;p Cyanobacteria	0.000	0.000	0.000	0.000	0.000	0.000	0.000	0.000	0.000	0.000	0.000	0.000	0.000	0.000	0.000
k Bacteria;p Deferribacteres	0.000	0.000	0.000	0.000	0.000	0.000	0.000	0.000	0.000	0.000	0.000	0.000	0.000	0.000	0.000
k Bacteria;p Firmicutes	0.089	0.045	0.118	0.893	0.129	0.404	0.313	0.214	0.100	0.026	0.194	0.380	0.432	0.033	0.155
k Bacteria;p Fusobacteria	0.000	0.000	0.000	0.000	0.000	0.000	0.000	0.000	0.000	0.000	0.000	0.000	0.000	0.000	0.000
k Bacteria;p Proteobacteria	0.012	0.004	0.050	0.102	0.159	0.367	0.033	0.131	0.227	0.018	0.268	0.092	0.028	0.516	0.117
k Bacteria;p Synergistetes	0.000	0.000	0.000	0.000	0.000	0.000	0.000	0.000	0.000	0.000	0.000	0.000	0.000	0.000	0.000
k Bacteria;p Tenericutes	0.000	0.000	0.000	0.000	0.000	0.000	0.000	0.000	0.000	0.000	0.000	0.000	0.000	0.000	0.000
k Bacteria;p Verrucomicrobia	0.000	0.000	0.000	0.000	0.000	0.000	0.000	0.000	0.000	0.000	0.000	0.000	0.000	0.000	0.000
Unassigned;Other	0.000	0.000	0.000	0.000	0.000	0.000	0.000	0.001	0.000	0.002	0.000	0.001	0.000	0.000	0.001

Supplemental Table 7. Detailed results of 16S rRNA gene sequence analysis at the genus levels.

Type	preultivated OF															postcultivated OF in BM for 48h															postcultivated OF in YCFA for 48h															IF														
Subject ID	ID01	ID02	ID03	ID04	ID05	ID06	ID07	ID08	ID09	ID10	ID11	ID12	ID13	ID14	ID15	ID01	ID02	ID03	ID04	ID05	ID06	ID07	ID08	ID09	ID10	ID11	ID12	ID13	ID14	ID15	ID01	ID02	ID03	ID04	ID05	ID06	ID07	ID08	ID09	ID10	ID11	ID12	ID13	ID14	ID15	ID01	ID02	ID03	ID04	ID05	ID06	ID07	ID08	ID09	ID10	ID11	ID12	ID13	ID14	ID15
k__Bacteria;p__Actinobacteria;c__Actinobacteria;o__Actinomycetales;f__Actinomycetaceae;g__Actinobaculum	0.000	0.000	0.000	0.000	0.000	0.000	0.000	0.000	0.000	0.000	0.000	0.000	0.000	0.000	0.000	0.000	0.000	0.000	0.000	0.000	0.000	0.000	0.000	0.000	0.000	0.000	0.000	0.000	0.000	0.000	0.000	0.000	0.000	0.000	0.000	0.000	0.000	0.000	0.000	0.000	0.000	0.000	0.000	0.000	0.000	0.000	0.000	0.000	0.000	0.000	0.000	0.000	0.000	0.000	0.000	0.000	0.000	0.000	0.000	0.000
k__Bacteria;p__Actinobacteria;c__Actinobacteria;o__Actinomycetales;f__Actinomycetaceae;g__Actinomyces	0.000	0.000	0.000	0.000	0.000	0.003	0.000	0.000	0.000	0.000	0.000	0.000	0.000	0.007	0.000	0.000	0.000	0.000	0.000	0.000	0.000	0.000	0.000	0.000	0.000	0.000	0.000	0.000	0.000	0.000	0.000	0.000	0.000	0.000	0.000	0.000	0.000	0.000	0.000	0.001	0.000	0.000	0.000	0.000	0.000	0.000	0.000	0.000	0.000	0.000	0.225	0.000	0.000	0.004	0.000	0.001	0.001	0.000	0.000	0.000
k__Bacteria;p__Actinobacteria;c__Actinobacteria;o__Actinomycetales;f__Actinomycetaceae;g__Arcanobacterium	0.000	0.000	0.000	0.000	0.000	0.002	0.000	0.000	0.000	0.000	0.000	0.000	0.000	0.000	0.000	0.000	0.000	0.000	0.000	0.000	0.000	0.000	0.000	0.000	0.000	0.000	0.000	0.000	0.000	0.000	0.000	0.000	0.000	0.000	0.000	0.000	0.000	0.000	0.000	0.000	0.000	0.000	0.000	0.000	0.000	0.000	0.000	0.000	0.000	0.000	0.000	0.000	0.000	0.000	0.000	0.000	0.000	0.000	0.000	0.000
k__Bacteria;p__Actinobacteria;c__Actinobacteria;o__Actinomycetales;f__Actinomycetaceae;g__Mobiluncus	0.000	0.001	0.000	0.000	0.000	0.001	0.000	0.005	0.000	0.000	0.001	0.000	0.000	0.000	0.000	0.000	0.000	0.000	0.000	0.000	0.000	0.000	0.000	0.000	0.000	0.000	0.000	0.000	0.000	0.000	0.000	0.000	0.000	0.000	0.000	0.000	0.000	0.000	0.000	0.000	0.000	0.000	0.000	0.000	0.000	0.000	0.000	0.000	0.000	0.000	0.000	0.000	0.000	0.000	0.000	0.000	0.000	0.000	0.000	0.000
k__Bacteria;p__Actinobacteria;c__Actinobacteria;o__Actinomycetales;f__Actinomycetaceae;g__Varibaculum	0.000	0.000	0.000	0.000	0.000	0.002	0.000	0.000	0.000	0.000	0.000	0.000	0.000	0.002	0.000	0.000	0.000	0.000	0.000	0.000	0.000	0.000	0.000	0.000	0.000	0.000	0.000	0.000	0.000	0.000	0.000	0.000	0.000	0.000	0.000	0.000	0.000	0.000	0.000	0.000	0.000	0.000	0.000	0.000	0.000	0.000	0.002	0.000	0.000	0.000	0.000	0.000	0.000	0.000	0.000	0.001	0.000	0.000	0.000	0.000
k__Bacteria;p__Actinobacteria;c__Actinobacteria;o__Actinomycetales;f__Corynebacteriaceae;g__Corynebacterium	0.000	0.002	0.000	0.001	0.005	0.002	0.000	0.000	0.002	0.000	0.005	0.000	0.002	0.082	0.000	0.000	0.000	0.000	0.000	0.000	0.000	0.000	0.000	0.000	0.000	0.000	0.000	0.000	0.000	0.000	0.000	0.000	0.000	0.000	0.000	0.000	0.000	0.000	0.000	0.000	0.000	0.000	0.000	0.000	0.000	0.000	0.000	0.000	0.000	0.000	0.000	0.001	0.000	0.000	0.000	0.000	0.000	0.000	0.000	0.000
k__Bacteria;p__Actinobacteria;c__Actinobacteria;o__Actinomycetales;f__Micrococcaceae;g__Micrococcus	0.000	0.000	0.000	0.000	0.000	0.000	0.000	0.000	0.000	0.000	0.000	0.000	0.000	0.000	0.000	0.000	0.000	0.000	0.000	0.000	0.000	0.000	0.000	0.000	0.000	0.000	0.000	0.000	0.000	0.000	0.000	0.000	0.000	0.000	0.000	0.000	0.000	0.000	0.000	0.000	0.000	0.000	0.000	0.000	0.000	0.000	0.000	0.000	0.001	0.000	0.001	0.004	0.000	0.000	0.000	0.000	0.000	0.000	0.000	0.000
k__Bacteria;p__Actinobacteria;c__Actinobacteria;o__Actinomycetales;f__Micrococcaceae;g__Rothia	0.000	0.000	0.000	0.000	0.000	0.000	0.000	0.000	0.000	0.000	0.000	0.000	0.000	0.000	0.000	0.000	0.000	0.000	0.000	0.000	0.000	0.000	0.000	0.000	0.000	0.000	0.000	0.000	0.000	0.000	0.000	0.000	0.000	0.000	0.000	0.000	0.000	0.000	0.000	0.000	0.000	0.000	0.000	0.000	0.000	0.000	0.000	0.000	0.000	0.000	0.000	0.000	0.000	0.000	0.000	0.000	0.000	0.002	0.000	0.000
k__Bacteria;p__Actinobacteria;c__Actinobacteria;o__Actinomycetales;f__Nocardioidaceae;g__Propionicimonas	0.000	0.000	0.000	0.000	0.000	0.000	0.000	0.000	0.000	0.000	0.000	0.000	0.000	0.000	0.000	0.000	0.000	0.000	0.000	0.000	0.000	0.000	0.000	0.000	0.000	0.000	0.000	0.000	0.000	0.000	0.000	0.000	0.000	0.000	0.000	0.000	0.000	0.000	0.000	0.000	0.000	0.000	0.000	0.000	0.000	0.000	0.000	0.000	0.000	0.000	0.000	0.000	0.000	0.000	0.000	0.004	0.006	0.000	0.000	0.000
k__Bacteria;p__Actinobacteria;c__Actinobacteria;o__Actinomycetales;f__Propionibacteriaceae;g__Propionibacterium	0.000	0.000	0.000	0.000	0.000	0.000	0.000	0.000	0.000	0.000	0.000	0.001	0.000	0.000	0.000	0.000	0.000	0.000	0.000	0.000	0.000	0.000	0.000	0.000	0.000	0.000	0.000	0.000	0.000	0.000	0.000	0.000	0.000	0.000	0.000	0.000	0.000	0.000	0.000	0.000	0.000	0.000	0.000	0.000	0.000	0.000	0.000	0.000	0.000	0.000	0.000	0.000	0.000	0.000	0.000	0.000	0.000	0.000	0.000	0.000
k__Bacteria;p__Actinobacteria;c__Actinobacteria;o__Actinomycetales;f__Propionibacteriaceae;Other	0.000	0.000	0.000	0.000	0.000	0.000	0.000	0.000	0.000	0.000	0.000	0.000	0.000	0.000	0.000	0.000	0.000	0.000	0.000	0.000	0.000	0.000	0.000	0.000	0.000	0.000	0.000	0.000	0.000	0.000	0.000	0.000	0.000	0.000	0.000	0.000	0.000	0.000	0.000	0.000	0.000	0.000	0.000	0.000	0.000	0.000	0.000	0.000	0.000	0.000	0.000	0.000	0.000	0.000	0.000	0.000	0.000	0.000	0.000	0.000
k__Bacteria;p__Actinobacteria;c__Actinobacteria;o__Bifidobacteriales;f__Bifidobacteriaceae;g__Bifidobacterium	0.019	0.038	0.986	0.019	0.019	0.045	0.093	0.017	0.001	0.153	0.017	0.299	0.338	0.178	0.017	0.000	0.001	0.863	0.001	0.052	0.000	0.550	0.010	0.000	0.000	0.000	0.367	0.349	0.000	0.002	0.001	0.186	0.262	0.001	0.000	0.001	0.000	0.002	0.017	0.002	0.002	0.333	0.013	0.001	0.002	0.897	0.844	0.814	0.001	0.183	0.001	0.644	0.653	0.514	0.953	0.531	0.466	0.498	0.001	0.641
k__Bacteria;p__Actinobacteria;c__Actinobacteria;o__Bifidobacteriales;f__Bifidobacteriaceae;Other	0.000	0.000	0.000	0.000	0.000	0.000	0.000	0.588	0.324	0.000	0.001	0.054	0.000	0.000	0.000	0.000	0.000	0.000	0.000	0.000	0.000	0.000	0.000	0.000	0.000	0.000	0.000	0.000	0.000	0.000	0.000	0.000	0.000	0.000	0.000	0.000	0.000	0.000	0.001	0.000	0.000	0.029	0.000	0.000	0.000	0.000	0.000	0.000	0.000	0.000	0.000	0.000	0.000	0.000	0.000	0.000	0.000	0.000	0.000	0.000
k__Bacteria;p__Acidobacteria;c__[Chloracidobacteria];o__RB41;f__Ellin6075;g__	0.000	0.000	0.000	0.000	0.000	0.000	0.000	0.000	0.000	0.000	0.000	0.000	0.000	0.000	0.000	0.000	0.000	0.000	0.000	0.000	0.000	0.000	0.000	0.000	0.000	0.000	0.000	0.000	0.000	0.000	0.000	0.000	0.000	0.000	0.000	0.000	0.000	0.000	0.000	0.000	0.000	0.000	0.000	0.000	0.000	0.000	0.002	0.000	0.000	0.000	0.000	0.000	0.000	0.000	0.000	0.000	0.000	0.000	0.000	0.000
k__Bacteria;p__Actinobacteria;c__Coriobacteriia;o__Coriobacteriales;f__Coriobacteriaceae;g__	0.000	0.000	0.000	0.000	0.000	0.000	0.000	0.003	0.007	0.000	0.000	0.000	0.000	0.000	0.000	0.000	0.000	0.000	0.000	0.000	0.000	0.000	0.000	0.000	0.000	0.000	0.000	0.000	0.000	0.000	0.000	0.000	0.000	0.000	0.000	0.000	0.000	0.000	0.000	0.000	0.000	0.000	0.000	0.000	0.000	0.000	0.000	0.000	0.000	0.000	0.000	0.000	0.000	0.000	0.000	0.000	0.000	0.000	0.000	0.000
k__Bacteria;p__Actinobacteria;c__Coriobacteriia;o__Coriobacteriales;f__Coriobacteriaceae;g__Adlercreutzia	0.000	0.000	0.000	0.000	0.000	0.000	0.000	0.000	0.000	0.000	0.000	0.000	0.000	0.000	0.000	0.000	0.000	0.000	0.000	0.000	0.000	0.000	0.000	0.000	0.000	0.000	0.000	0.000	0.000	0.000	0.000	0.000	0.000	0.000	0.000	0.000	0.000	0.000	0.000	0.000	0.000	0.000	0.000	0.000	0.000	0.000	0.000	0.000	0.000	0.000	0.000	0.005	0.000	0.000	0.000	0.000	0.000	0.000	0.000	0.000
k__Bacteria;p__Actinobacteria;c__Coriobacteriia;o__Coriobacteriales;f__Coriobacteriaceae;g__Atopobium	0.000	0.000	0.000	0.000	0.000	0.001	0.000	0.045	0.079	0.000	0.000	0.000	0.000	0.000	0.000	0.000	0.000	0.000	0.000	0.000	0.000	0.000	0.000	0.000	0.000	0.000	0.000	0.000	0.000	0.000	0.000	0.004	0.000	0.000	0.007	0.000	0.000	0.000	0.000	0.000	0.000	0.000	0.002	0.001	0.000	0.000	0.100	0.000	0.000	0.108	0.000	0.000	0.000	0.000	0.000	0.000	0.000	0.000	0.000	0.000
k__Bacteria;p__Actinobacteria;c__Coriobacteriia;o__Coriobacteriales;f__Coriobacteriaceae;g__Collinsella	0.000	0.000	0.000	0.000	0.000	0.000	0.004	0.000	0.000	0.000	0.000	0.000	0.003	0.001	0.000	0.000	0.000	0.000	0.000	0.000	0.000	0.000	0.000	0.000	0.000	0.000	0.000	0.000	0.000	0.000	0.000	0.000	0.000	0.000	0.000	0.000	0.000	0.000	0.000	0.000	0.000	0.000	0.002	0.000	0.000	0.000	0.000	0.002	0.000	0.000	0.000	0.000	0.000	0.000	0.000	0.000	0.000	0.000	0.000	0.000
k__Bacteria;p__Actinobacteria;c__Coriobacteriia;o__Coriobacteriales;f__Coriobacteriaceae;g__Eggerthella	0.000	0.000	0.000	0.000	0.000	0.000	0.000	0.000	0.000	0.000	0.000	0.000	0.000	0.000	0.000	0.000	0.000	0.000	0.000	0.000	0.000	0.000	0.000	0.000	0.000	0.000	0.000	0.000	0.000	0.000	0.000	0.000	0.000	0.000	0.000	0.000	0.000	0.000	0.000	0.000	0.000	0.000	0.000	0.000	0.000	0.000	0.000	0.000	0.000	0.000	0.000	0.000	0.000	0.000	0.000	0.000	0.000	0.000	0.000	0.000
k__Bacteria;p__Actinobacteria;c__Coriobacteriia;o__Coriobacteriales;f__Coriobacteriaceae;g__Slackia	0.000	0.000	0.000	0.000	0.000	0.000	0.000	0.000	0.000	0.000	0.000	0.000	0.000	0.000	0.000	0.000	0.000	0.000	0.000	0.000	0.000	0.000	0.000	0.000	0.000	0.000	0.000	0.000	0.000	0.000	0.000	0.000	0.000	0.000	0.000	0.000	0.000	0.000	0.000	0.000	0.000	0.000	0.000	0.000	0.000	0.000	0.000	0.000	0.000	0.000	0.000	0.000	0.000	0.000	0.000	0.000	0.000	0.000	0.000	0.000
k__Bacteria;p__Bacteroidetes;c__[Saprospirae];o__[Saprospirales];f__Chitinophagaceae;g__	0.000	0.000	0.000	0.000	0.000	0.000	0.000	0.000	0.000	0.000	0.000	0.000	0.000	0.000	0.000	0.000	0.000	0.000	0.000	0.000	0.000	0.000	0.000	0.000	0.000	0.000	0.000	0.000	0.000	0.000	0.000	0.000	0.000	0.000	0.000	0.000	0.000	0.000	0.000	0.000	0.000	0.000	0.000	0.000	0.000	0.000	0.000	0.000	0.000	0.000	0.000	0.000	0.000	0.000	0.000	0.000	0.000	0.000	0.000	0.000
k__Bacteria;p__Bacteroidetes;c__Bacteroidia;o__Bacteroidales;f__;g__	0.000	0.000	0.000	0.000	0.000	0.000	0.000	0.000	0.000	0.000	0.000	0.000	0.000	0.000	0.000	0.000	0.000	0.000	0.000	0.000	0.000	0.000	0.000	0.000	0.000	0.000	0.000	0.000	0.000	0.000	0.000	0.000	0.000	0.000	0.000	0.000	0.000	0.000	0.000	0.000	0.000	0.000	0.000	0.000	0.000	0.000	0.000	0.000	0.000	0.000	0.000	0.000	0.000	0.000	0.000	0.000	0.000	0.000	0.000	0.000
k__Bacteria;p__Bacteroidetes;c__Bacteroidia;o__Bacteroidales;f__[Barnesiellaceae];g__	0.000	0.000	0.000	0.000	0.000	0.000	0.000	0.000	0.000	0.000	0.000	0.000	0.000	0.000	0.000	0.000	0.000	0.000	0.000	0.000	0.000	0.000	0.000	0.000	0.000	0.000	0.000	0.000	0.000	0.000	0.000	0.000	0.000	0.000	0.000	0.000	0.000	0.000	0.000	0.000	0.000	0.000	0.000	0.000	0.000	0.000	0.000	0.000	0.000	0.000	0.000	0.000	0.000	0.000	0.000	0.000	0.000	0.000	0.000	0.000
k__Bacteria;p__Bacteroidetes;c__Bacteroidia;o__Bacteroidales;f__[Odoribacteraceae];g__Butyricimonas	0.000	0.000	0.000	0.000	0.000	0.000	0.000	0.000	0.000	0.000	0.000	0.000	0.000	0.000	0.000	0.000	0.000	0.000	0.000	0.000	0.000	0.000	0.000	0.000	0.000	0.000	0.000	0.000	0.000	0.000	0.000	0.000	0.000	0.000	0.000	0.000	0.000	0.000	0.000	0.000	0.000	0.000	0.000	0.000	0.000	0.000	0.000	0.000	0.000	0.000	0.000	0.000	0.000	0.000	0.000	0.000	0.000	0.000	0.000	0.000
k__Bacteria;p__Bacteroidetes;c__Bacteroidia;o__Bacteroidales;f__[Odoribacteraceae];g__Odoribacter	0.000	0.000	0.000	0.000	0.000	0.000	0.000	0.000	0.000	0.000	0.000	0.000	0.000	0.000	0.000	0.000	0.000	0.000	0.000	0.000	0.000	0.000	0.000	0.000	0.000	0.000	0.000	0.000	0.000	0.000	0.000	0.000	0.000	0.000	0.000	0.000	0.000	0.000	0.000	0.000	0.000	0.000	0.000	0.000	0.000	0.000	0.000	0.000	0.000	0.000	0.000	0.000	0.000	0.000	0.000	0.000	0.000	0.000	0.000	0.000
k__Bacteria;p__Bacteroidetes;c__Bacteroidia;o__Bacteroidales;f__[Paraprevotellaceae];g__[Prevotella]	0.000	0.000	0.000	0.000	0.000	0.000	0.000	0.000	0.000	0.000	0.000	0.000	0.000	0.000	0.000	0.000	0.000	0.000	0.000	0.000	0.000	0.000	0.000	0.000	0.000	0.000	0.000	0.000	0.000	0.000	0.000	0.000	0.000	0.000	0.000	0.000	0.001	0.000	0.000	0.000	0.000	0.000	0.000	0.000	0.000	0.000	0.000	0.000	0.000	0.000	0.000	0.000	0.000	0.000	0.000	0.000	0.000	0.000	0.000	0.000
k__Bacteria;p__Bacteroidetes;c__Bacteroidia;o__Bacteroidales;f__[Paraprevotellaceae];g__CF231	0.000	0.000	0.000	0.000	0.000	0.000	0.000	0.000	0.000	0.000	0.000	0.002	0.000	0.001	0.000	0.000	0.000	0.000	0.000	0.000	0.000	0.000	0.000	0.000	0.000	0.000	0.000	0.000	0.000	0.000	0.000	0.000	0.000	0.000	0.000	0.000	0.000	0.000	0.000	0.000	0.000	0.000	0.000	0.000	0.000	0.000	0.000	0.000	0.000	0.000	0.000	0.000	0.000	0.000	0.000	0.000	0.000	0.000	0.000	0.000
k__Bacteria;p__Bacteroidetes;c__Bacteroidia;o__Bacteroidales;f__[Paraprevotellaceae];g__Paraprevotella	0.000	0.000	0.000	0.000	0.000	0.000	0.000	0.000	0.000	0.000	0.000	0.001	0.001	0.000	0.000	0.000	0.000	0.000	0.000	0.000	0.000	0.000	0.000	0.000	0.000	0.000	0.000	0.000	0.000	0.000	0.000	0.000	0.000	0.000	0.000	0.000	0.000	0.000	0.000	0.000	0.000	0.000	0.000	0.000	0.000	0.000	0.000	0.000	0.000	0.000	0.000	0.000	0.000	0.000	0.000	0.000	0.000	0.000	0.000	0.000
k__Bacteria;p__Bacteroidetes;c__Bacteroidia;o__Bacteroidales;f__Bacteroidaceae;g__Bacteroides	0.001	0.003	0.001	0.001	0.001	0.347	0.024	0.041	0.001	0.020	0.001	0.021	0.015	0.013	0.001	0.000	0.000	0.000	0.000	0.000	0.000	0.000	0.000	0.000	0.000	0.000	0.000	0.000	0.000	0.000	0.001	0.001	0.002	0.763	0.782	0.000	0.012	0.000	0.270	0.037	0.000	0.328	0.368	0.023	0.001	0.001	0.002	0.014	0.001	0.372	0.001	0.000	0.001	0.153	0.001	0.000	0.054	0.031	0.449	0.085
k__Bacteria;p__Bacteroidetes;c__Bacteroidia;o__Bacteroidales;f__Porphyromonadaceae;g__Parabacteroides	0.000	0.000	0.000	0.000	0.000	0.001	0.007	0.000	0.000	0.000	0.000	0.001	0.005	0.001	0.000	0.000	0.000	0.000	0.000	0.000	0.000	0.000	0.000	0.000	0.000	0.000	0.000	0.000	0.000	0.000	0.000	0.000	0.000	0.000	0.000	0.000	0.003	0.000	0.000	0.000	0.000	0.000	0.001	0.006	0.000	0.000	0.000	0.000	0.000	0.050	0.000	0.000	0.000	0.000	0.000	0.000	0.000	0.008	0.000	0.000
k__Bacteria;p__Bacteroidetes;c__Bacteroidia;o__Bacteroidales;f__Porphyromonadaceae;g__Porphyromonas	0.000	0.000	0.000	0.000	0.000	0.048	0.000	0.000	0.000	0.000	0.000	0.000	0.000	0.009	0.000	0.000	0.000	0.000	0.000	0.000	0.000	0.000	0.000	0.000	0.000	0.000	0.000	0.000	0.000	0.000	0.000	0.000	0.000	0.000	0.000	0.000	0.000	0.000	0.000	0.000	0.000	0.000	0.000	0.000	0.000	0.000	0.000	0.000	0.000	0.000	0.000	0.000	0.000	0.000	0.000	0.000	0.000	0.000	0.000	0.000
k__Bacteria;p__Bacteroidetes;c__Bacteroidia;o__Bacteroidales;f__Prevotellaceae;g__Prevotella	0.000	0.004	0.001	0.000	0.001	0.070	0.028	0.049	0.197	0.010	0.004	0.012	0.033	0.025	0.000	0.000	0.000	0.000	0.000	0.000	0.000	0.000	0.000	0.000	0.000	0.000	0.000	0.000	0.003	0.000	0.000	0.000	0.000	0.000	0.000	0.000	0.000	0.051	0.000	0.081	0.102	0.000	0.000	0.056	0.000	0.000	0.000	0.000	0.000	0.000	0.001	0.000	0.000	0.000	0.000	0.000	0.000	0.000	0.000	0.000
k__Bacteria;p__Bacteroidetes;c__Bacteroidia;o__Bacteroidales;f__Rikenellaceae;g__	0.000	0.000	0.000	0.000	0.000	0.001	0.000	0.000	0.000	0.001	0.000	0.000	0.000	0.000	0.000	0.000	0.000	0.000	0.000	0.000	0.000	0.000	0.000	0.000	0.000	0.000	0.000	0.000	0.000	0.000	0.000	0.000	0.000	0.000	0.000	0.000	0.000	0.000	0.000	0.000	0.000	0.000	0.000	0.000	0.000	0.000	0.000	0.000	0.000	0.000	0.000	0.000	0.000	0.000	0.000	0.000	0.000	0.000	0.000	0.000
k__Bacteria;p__Bacteroidetes;c__Bacteroidia;o__Bacteroidales;f__Rikenellaceae;Other	0.000	0.000	0.000	0.000	0.000	0.000	0.000	0.000	0.000	0.000	0.000	0.000	0.000	0.000	0.000	0.000	0.000	0.000	0.000	0.000	0.000	0.000	0.000	0.000	0.000	0.000	0.000	0.000	0.000	0.000	0.000	0.000	0.000	0.000	0.000	0.000	0.000	0.000	0.000	0.000	0.000	0.000	0.000	0.000	0.000	0.000	0.000	0.000	0.000	0.000	0.000	0.000	0.000	0.000	0.000	0.000	0.000	0.000	0.000	0.000
k__Bacteria;p__Bacteroidetes;c__Bacteroidia;o__Bacteroidales;f__S24-7;g__	0.000	0.000	0.000	0.000	0.000	0.010	0.002	0.000	0.000	0.005	0.000	0.004	0.009	0.004	0.000	0.000	0.000	0.000	0.000	0.000	0.000	0.000	0.000	0.000	0.000	0.000	0.000	0.000	0.000	0.000	0.000	0.000	0.000	0.000	0.000	0.000	0.000	0.000	0.000	0.000	0.000	0.000	0.000	0.000	0.000	0.000	0.000	0.000	0.000	0.000	0.000	0.000	0.000	0.000	0.000	0.000	0.000	0.000	0.000	0.000
k__Bacteria;p__Bacteroidetes;c__Flavobacteriia;o__Flavobacteriales;f__[Weeksellaceae];g__Cloacibacterium	0.000	0.000	0.000	0.000	0.000	0.000	0.000	0.000	0.000	0.000	0.000	0.000	0.003	0.001	0.000	0.000	0.000	0.000	0.000	0.000	0.000	0.000	0.000	0.000	0.000	0.000	0.000	0.000	0.000	0.000	0.000	0.000	0.000	0.000	0.000	0.000	0.000	0.000	0.000	0.000	0.000	0.000	0.000	0.000	0.000	0.000	0.000	0.000	0.000	0.000	0.000	0.000	0.000	0.000	0.000	0.000	0.000	0.000	0.000	0.000
k__Bacteria;p__Chloroflexi;c__TK10;o__AKYG885;f__Dolo_23;g__	0.000	0.000	0.000	0.000	0.000	0.000	0.000	0.000	0.000	0.000	0.000	0.000	0.000	0.000	0.000	0.000	0.000	0.000	0.000	0.000	0.000	0.000	0.000	0.000	0.000	0.000	0.000	0.000	0.000	0.000	0.000	0.000	0.000	0.000	0.000	0.000	0.000	0.000	0.000	0.000	0.000	0.000	0.000	0.000	0.000	0.000	0.000	0.000	0.000	0.000	0.000	0.000	0.000	0.000	0.000	0.000	0.000	0.000	0.000	0.000
k__Bacteria;p__Cyanobacteria;c__4C0d-2;o__MLE1-12;f__;g__	0.000	0.000	0.000	0.000	0.000	0.000	0.000	0.000	0.000	0.000	0.000	0.000	0.000	0.000	0.000	0.000	0.000	0.000	0.000	0.000	0.000	0.000	0.000	0.000	0.000	0.000	0.000	0.000	0.000	0.000	0.000	0.000	0.000	0.000	0.000	0.000	0.000	0.000	0.000	0.000	0.000	0.000	0.000	0.000	0.000	0.000	0.000	0.000	0.000	0.000	0.000	0.000	0.000	0.000	0.000	0.000	0.000	0.000	0.000	0.000
k__Bacteria;p__Cyanobacteria;c__Chloroplast;o__Streptophyta;f__;g__	0.000	0.000	0.000	0.000	0.000	0.000	0.000	0.000	0.000	0.000	0.000	0.000	0.000	0.000	0.000	0.000	0.000	0.000	0.000	0.000	0.000	0.000	0.000	0.000	0.000	0.000	0.000	0.000	0.000	0.000	0.000	0.000	0.000	0.000	0.000	0.000	0.000	0.000	0.000	0.000	0.000	0.000	0.000	0.000	0.000	0.000	0.000	0.000	0.000	0.000	0.000	0.000	0.000	0.000	0.000	0.000	0.000	0.000	0.000	0.000
k__Bacteria;p__Deferribacteres;c__Deferribacteres;o__Deferribacterales;f__Deferribacteraceae;g__Mucispirillum	0.000	0.000	0.000	0.000	0.000	0.000	0.000	0.000	0.000	0.000	0.000	0.000	0.000	0.000	0.000	0.000	0.000	0.000	0.000	0.000	0.000	0.000	0.000	0.000	0.000	0.000	0.000	0.000	0.000	0.000	0.000	0.000	0.000	0.000	0.000	0.000	0.000	0.000	0.000	0.000	0.000	0.000	0.000	0.000	0.000	0.000	0.000	0.000	0.000	0.000	0.000	0.000	0.000	0.000	0.000	0.000	0.000	0.000	0.000	0.000
k__Bacteria;p__Firmicutes;c__Bacilli;o__Bacillales;f__Bacillaceae;g__	0.000	0.000	0.000	0.000	0.000	0.000	0.000	0.000	0.000	0.000	0.000	0.000	0.000	0.000	0.000	0.000	0.000	0.000	0.000	0.000	0.000	0.000	0.000	0.000	0.000	0.000	0.000	0.000	0.000	0.000	0.000	0.000	0.000	0.000	0.000	0.000	0.000	0.000	0.000	0.000	0.000	0.000	0.000	0.000	0.000	0.000	0.000	0.000	0.000	0.000	0.000	0.000	0.000	0.000	0.000	0.000	0.000	0.000	0.000	0.000
k__Bacteria;p__Firmicutes;c__Bacilli;o__Bacillales;f__Bacillaceae;g__Bacillus	0.000	0.000	0.000	0.000	0.000	0.000	0.000	0.000	0.000	0.000	0.000	0.000	0.000	0.000	0.000	0.000	0.000	0.000	0.000	0.000	0.000	0.000	0.000	0.000	0.000	0.000	0.000	0.000	0.000	0.000	0.000	0.000	0.000	0.000	0.000	0.000	0.000	0.000	0.000	0.000	0.000	0.000	0.000	0.000	0.000	0.000	0.000	0.000	0.000	0.000	0.000	0.000	0.000	0.000	0.000	0.000	0.000	0.000	0.000	0.000
k__Bacteria;p__Firmicutes;c__Bacilli;o__Bacillales;f__Bacillaceae;Other	0.000	0.000	0.000	0.000	0.000	0.000	0.000	0.000	0.000	0.000	0.000	0.000	0.000	0.000	0.000	0.000	0.000	0.000	0.000	0.000	0.000	0.000	0.000	0.000	0.000	0.000	0.000	0.000	0.000	0.000	0.000	0.000	0.000	0.000	0.000	0.000	0.000	0.000	0.000	0.000	0.000	0.000	0.000	0.000	0.000	0.000	0.000	0.000	0.000	0.000	0.000	0.000	0.000	0.000	0.000	0.000	0.000	0.000	0.000	0.000
k__Bacteria;p__Firmicutes;c__Bacilli;o__Bacillales;f__Paenibacillaceae;g__Brevibacillus	0.000	0.000	0.000	0.000	0.000	0.000	0.000	0.000	0.000	0.000	0.000	0.000	0.000	0.000	0.000	0.000	0.000	0.000	0.000	0.000	0.000	0.000	0.000	0.000	0.000	0.000	0.000	0.000	0.000	0.000	0.000	0.000	0.000	0.000	0.000	0.000	0.000	0.000	0.000	0.000	0.000	0.000	0.000	0.000	0.000	0.000	0.000	0.000	0.000	0.000	0.000	0.000	0.000	0.000	0.000	0.000	0.000	0.000	0.000	0.000
k__Bacteria;p__Firmicutes;c__Bacilli;o__Bacillales;f__Planococcaceae;g__	0.000	0.000	0.000	0.000	0.000	0.000	0.000	0.000	0.000	0.000	0.000	0.001	0.000	0.002	0.000	0.000	0.000	0.000	0.000	0.000	0.000	0.000	0.000	0.000	0.000	0.000	0.000	0.000	0.000	0.000	0.011	0.000	0.000	0.000	0.000	0.001	0.000	0.000	0.000	0.000	0.000	0.000	0.000	0.000	0.000	0.000	0.000	0.001	0.000	0.000	0.000	0.002	0.000	0.001	0.000	0.001	0.001	0.000	0.000	0.000
k__Bacteria;p__Firmicutes;c__Bacilli;o__Bacillales;f__Staphylococcaceae;g__Staphylococcus	0.000	0.002	0.000	0.001	0.003	0.000	0.013	0.000	0.000	0.009	0.006	0.003	0.011	0.074	0.000	0.000	0.003	0.000	0.000	0.000	0.000	0.000	0.000	0.001	0.000	0.001	0.000	0.000	0.000	0.000	0.934	0.000	0.000	0.000	0.000	0.098	0.000	0.000	0.000	0.000	0.000	0.000	0.002	0.000	0.005	0.012	0.009	0.052	0.025	0.023	0.020	0.120	0.011	0.033	0.002	0.024	0.096	0.056	0.010	0.003
k__Bacteria;p__Firmicutes;c__Bacilli;o__Bacillales;Other;Other	0.000	0.000	0.000	0.000	0.000	0.000	0.000	0.000	0.000	0.000	0.000	0.000	0.000	0.000	0.000	0.000	0.000	0.000	0.000	0.000	0.000	0.000	0.000	0.000	0.000	0.000	0.000	0.000	0.000	0.000	0.000	0.000	0.000	0.000	0.000	0.000	0.000	0.000	0.000	0.000	0.000	0.000	0.000	0.000	0.000	0.002	0.000	0.000	0.001	0.000	0.000	0.000	0.000	0.002	0.000	0.000	0.000	0.000	0.000	0.000
k__Bacteria;p__Firmicutes;c__Bacilli;o__Gemellales;f__Gemellaceae;g__	0.000	0.000	0.000	0.000	0.000	0.000	0.000	0.000	0.000	0.000	0.000	0.000	0.000	0.000	0.000	0.000	0.000	0.000	0.000	0.000	0.000	0.000	0.000	0.000	0.000	0.000	0.000	0.000	0.000	0.000	0.000	0.000	0.000	0.000	0.000	0.000	0.000	0.000	0.000	0.000	0.000	0.000	0.000	0.000	0.000	0.000	0.000	0.000	0.000	0.000	0.000	0.000	0.000	0.000	0.000	0.000	0.000	0.000	0.000	0.000
k__Bacteria;p__Firmicutes;c__Bacilli;o__Gemellales;f__Gemellaceae;g__Gemella	0.000	0.000	0.000	0.000	0.000	0.000	0.000	0.000	0.000	0.000	0.000	0.000	0.000	0.000	0.000	0.000	0.000	0.000	0.000	0.000	0.000	0.000	0.000	0.000	0.000	0.000	0.000	0.000	0.000	0.000	0.000	0.000	0.000	0.000	0.000	0.000	0.000	0.000	0.000	0.000	0.000	0.000	0.000	0.000	0.000	0.000	0.000	0.000	0.000	0.000	0.000	0.000	0.000	0.000	0.000	0.000	0.000	0.000	0.000	0.000
k__Bacteria;p__Firmicutes;c__Bacilli;o__Lactobacillales;f__;g__	0.000	0.000	0.000	0.000	0.000	0.000	0.001	0.000	0.000	0.000	0.000	0.000	0.000	0.001	0.000	0.002	0.003	0.000	0.003	0.002	0.002	0.001	0.002	0.003	0.002	0.003	0.001	0.002	0.001	0.002	0.000	0.001	0.000	0.000	0.000	0.000	0.002	0.001	0.000	0.001	0.000	0.000	0.000	0.000	0.000	0.000	0.000	0.000	0.000	0.000	0.000	0.000	0.000	0.000	0.000	0.001	0.000	0.001	0.000	0.000
k__Bacteria;p__Firmicutes;c__Bacilli;o__Lactobacillales;f__Aerococcaceae;g__Aerococcus	0.000	0.000	0.000	0.000	0.000	0.000	0.000	0.032	0.003	0.000	0.000	0.000	0.000	0.000	0.000	0.000	0.000	0.000	0.000	0.000	0.000	0.000	0.000	0.000	0.000	0.000	0.000	0.000	0.000	0.000	0.000	0.000	0.000	0.000	0.000	0.000	0.000	0.000	0.000	0.000	0.000	0.000	0.000	0.000	0.000	0.000	0.000	0.000	0.000	0.000	0.000	0.000	0.000	0.000	0.000	0.000	0.000	0.000	0.000	0.000
k__Bacteria;p__Firmicutes;c__Bacilli;o__Lactobacillales;f__Aerococcaceae;g__Alloiococcus	0.000	0.000	0.000	0.000	0.000	0.000	0.000	0.000	0.000	0.000	0.000	0.000	0.000	0.000	0.000	0.000	0.000	0.000	0.000	0.000	0.000	0.000	0.000	0.000	0.000	0.000	0.000	0.000	0.000	0.000	0.000	0.000	0.000	0.000	0.000	0.000	0.000	0.000	0.000	0.000	0.000	0.000	0.000	0.000	0.000	0.000	0.000	0.000	0.000	0.000	0.000	0.000	0.000	0.000	0.000	0.000	0.000	0.000	0.000	0.000
k__Bacteria;p__Firmicutes;c__Bacilli;o__Lactobacillales;f__Aerococcaceae;g__Facklamia	0.000	0.000	0.000	0.000	0.000	0.000	0.000	0.000	0.000	0.000	0.000	0.000	0.001	0.015	0.000	0.000	0.000	0.000	0.000	0.000	0.000	0.000	0.000	0.000	0.000	0.000	0.000	0.000	0.000	0.000	0.000	0.000	0.000	0.000	0.000	0.001	0.000	0.000	0.000	0.000	0.000	0.000	0.000	0.000	0.000	0.000	0.000	0.000	0.000	0.000	0.000	0.000	0.000	0.000	0.000	0.000	0.000	0.000	0.000	0.000
k__Bacteria;p__Firmicutes;c__Bacilli;o__Lactobacillales;f__Carnobacteriaceae;g__	0.000	0.000	0.000	0.000	0.000	0.000	0.000	0.000	0.000	0.000	0.000	0.000	0.000	0.000	0.000	0.000	0.000	0.000	0.000	0.000	0.000	0.000	0.000	0.000	0.000	0.000	0.000	0.000	0.000	0.000	0.000	0.000	0.000	0.000	0.000	0.000	0.000	0.000	0.000	0.000	0.000	0.000	0.000	0.000	0.000	0.000	0.000	0.000	0.000	0.000	0.000	0.000	0.000	0.000	0.000	0.000	0.000	0.000	0.000	0.000
k__Bacteria;p__Firmicutes;c__Bacilli;o__Lactobacillales;f__Enterococcaceae;g__	0.000	0.000	0.000	0.000	0.000	0.000	0.000	0.000	0.000	0.000	0.000	0.000	0.000	0.000	0.000	0.005	0.004	0.001	0.007	0.005	0.005	0.003	0.004	0.004	0.007	0.006	0.003	0.003	0.003	0.006	0.000	0.001	0.000	0.000	0.000	0.000	0.005	0.005	0.000	0.004	0.000	0.000	0.000	0.000	0.000	0.000	0.000	0.000	0.000	0.000	0.000	0.000	0.000	0.000	0.000	0.000	0.000	0.000	0.000	0.000
k__Bacteria;p__Firmicutes;c__Bacilli;o__Lactobacillales;f__Enterococcaceae;g__Enterococcus	0.001	0.004	0.001	0.002	0.003	0.076	0.096	0.003	0.001	0.084	0.003	0.103	0.168	0.071	0.002	0.987	0.966	0.129	0.902	0.887	0.960	0.441	0.979	0.985	0.985	0.964	0.622	0.631	0.559	0.975	0.003	0.291	0.001	0.001	0.001	0.000	0.868	0.929	0.001	0.711	0.004	0.000	0.002	0.040	0.000	0.000	0.003	0.001	0.001	0.001	0.001	0.001	0.192	0.000	0.015	0.040	0.000	0.123	0.000	0.000
k__Bacteria;p__Firmicutes;c__Bacilli;o__Lactobacillales;f__Enterococcaceae;Other	0.000	0.000	0.000	0.000	0.000	0.001	0.001	0.000	0.000	0.001	0.000	0.000	0.000	0.000	0.000	0.004	0.004	0.001	0.004	0.003	0.004	0.002	0.004	0.004	0.004	0.004	0.002	0.002	0.003	0.005	0.000	0.002	0.000	0.000	0.000	0.000	0.005	0.004	0.000	0.005	0.000	0.000	0.000	0.000	0.000	0.000	0.000	0.000	0.000	0.000	0.000	0.000	0.001	0.000	0.000	0.000	0.000	0.003	0.000	0.000
k__Bacteria;p__Firmicutes;c__Bacilli;o__Lactobacillales;f__Lactobacillaceae;g__Lactobacillus	0.970	0.909	0.003	0.971	0.611	0.048	0.061	0.105	0.015	0.520	0.951	0.186	0.021	0.072	0.970	0.000	0.004	0.000	0.082	0.048	0.027	0.001	0.000	0.001	0.000	0.000	0.001	0.010	0.025	0.008	0.017	0.001	0.000	0.229	0.003	0.224	0.000	0.000	0.000	0.000	0.000	0.308	0.000	0.000	0.018	0.000	0.000	0.000	0.001	0.000	0.000	0.121	0.001	0.000	0.001	0.000	0.034	0.020	0.001	0.000
k__Bacteria;p__Firmicutes;c__Bacilli;o__Lactobacillales;f__Lactobacillaceae;g__Pediococcus	0.000	0.000	0.000	0.000	0.000	0.000	0.000	0.001	0.000	0.001	0.000	0.000	0.000	0.000	0.000	0.000	0.000	0.000	0.000	0.000	0.000	0.000	0.000	0.000	0.000	0.000	0.000	0.000	0.000	0.000	0.000	0.000	0.000	0.000	0.000	0.000	0.001	0.000	0.000	0.000	0.000	0.000	0.000	0.000	0.000	0.000	0.000	0.001	0.000	0.000	0.000	0.000	0.000	0.000	0.000	0.000	0.000	0.006	0.000	0.000
k__Bacteria;p__Firmicutes;c__Bacilli;o__Lactobacillales;f__Streptococcaceae;g__Lactococcus	0.000	0.000	0.000	0.000	0.000	0.000	0.000	0.000	0.000	0.000	0.000	0.000	0.000	0.000	0.000	0.000	0.000	0.000	0.000	0.000	0.000	0.000	0.000	0.000	0.000	0.000	0.000	0.000	0.000	0.000	0.000	0.000	0.000	0.000	0.000	0.000	0.000	0.000	0.000	0.000	0.000	0.000	0.000	0.000	0.000	0.000	0.000	0.000	0.000	0.000	0.000	0.000	0.000	0.000	0.000	0.000	0.000	0.000	0.000	0.000
k__Bacteria;p__Firmicutes;c__Bacilli;o__Lactobacillales;f__Streptococcaceae;g__Streptococcus	0.001	0.005	0.002	0.000	0.001	0.033	0.512	0.001	0.000	0.114	0.004	0.221	0.250	0.096	0.001	0.000	0.000	0.005	0.000	0.000	0.000	0.001	0.000	0.001	0.001	0.020	0.002	0.002	0.010	0.001	0.001	0.511	0.731	0.001	0.000	0.000	0.000	0.000	0.573	0.002	0.767	0.000	0.001	0.344	0.945	0.073	0.010	0.062	0.117	0.089	0.298	0.065	0.007	0.062	0.004	0.090	0.247	0.192	0.001	0.104
k__Bacteria;p__Firmicutes;c__Bacilli;o__Lactobacillales;Other;Other	0.000	0.000	0.000	0.000	0.000	0.000	0.000	0.000	0.000	0.000	0.000	0.000	0.000	0.000	0.000	0.000	0.000	0.000	0.000	0.000	0.000	0.000	0.000	0.000	0.000	0.000	0.000	0.000	0.000	0.000	0.000	0.000	0.000	0.000	0.000	0.000	0.000	0.000	0.000	0.000	0.000	0.000	0.000	0.000	0.000	0.000	0.000	0.000	0.000	0.000	0.000	0.000	0.000	0.000	0.000	0.000	0.000	0.000	0.000	0.000
k__Bacteria;p__Firmicutes;c__Bacilli;o__Turicibacterales;f__Turicibacteraceae;g__Turicibacter	0.000	0.000	0.000	0.000	0.000	0.002	0.001	0.000	0.000	0.000	0.000	0.000	0.000	0.001	0.000	0.000	0.000	0.000	0.000	0.000	0.000	0.000	0.000	0.000	0.000	0.000	0.000	0.000	0.000	0.000	0.000	0.000	0.000	0.000	0.000	0.000	0.000	0.000	0.000	0.000	0.000	0.000	0.000	0.000	0.000	0.000	0.000	0.000	0.000	0.000	0.000	0.000	0.000	0.000	0.000	0.000	0.000	0.000	0.000	0.000
k__Bacteria;p__Firmicutes;c__Clostridia;o__Clostridiales;f__;g__	0.000	0.002	0.000	0.000	0.000	0.011	0.004	0.000	0.000	0.002	0.000	0.001	0.007	0.005	0.001	0.000	0.000	0.000	0.000	0.000	0.000	0.000	0.000	0.000	0.000	0.000	0.000	0.000	0.000	0.000	0.000	0.000	0.000	0.000	0.000	0.000	0.000	0.000	0.000	0.000	0.000	0.000	0.000	0.000	0.000	0.000	0.000	0.000	0.000	0.000	0.000	0.000	0.000	0.000	0.000	0.000	0.000	0.000	0.000	0.000
k__Bacteria;p__Firmicutes;c__Clostridia;o__Clostridiales;f__[Mogibacteriaceae];g__	0.000	0.000	0.000	0.000	0.000	0.018	0.000	0.000	0.000	0.000	0.000	0.000	0.000	0.000	0.000	0.000	0.000	0.000	0.000	0.000	0.000	0.000	0.000	0.000	0.000	0.000	0.000	0.000	0.000	0.000	0.000	0.000	0.000	0.000	0.000	0.000	0.000	0.000	0.000	0.000	0.000	0.000	0.000	0.000	0.000	0.000	0.000	0.000	0.000	0.000	0.000	0.000	0.000	0.000	0.000	0.000	0.000	0.000	0.000	0.000
k__Bacteria;p__Firmicutes;c__Clostridia;o__Clostridiales;f__[Mogibacteriaceae];g__Mogibacterium	0.000	0.000	0.000	0.000	0.000	0.011	0.000	0.000	0.000	0.000	0.000	0.000	0.000	0.000	0.000	0.000	0.000	0.000	0.000	0.000	0.000	0.000	0.000	0.000	0.000	0.000	0.000	0.000	0.000	0.000	0.000	0.000	0.000	0.000	0.000	0.000	0.000	0.000	0.000	0.000	0.000	0.000	0.000	0.000	0.000	0.000	0.000	0.000	0.000	0.000	0.000	0.000	0.000	0.000	0.000	0.000	0.000	0.000	0.000	0.000
k__Bacteria;p__Firmicutes;c__Clostridia;o__Clostridiales;f__[Tissierellaceae];g__1-68	0.000	0.000	0.000	0.000	0.000	0.021	0.000	0.000	0.000	0.000	0.000	0.000	0.000	0.023	0.000	0.000	0.000	0.000	0.000	0.000	0.000	0.000	0.000	0.000	0.000	0.000	0.000	0.000	0.000	0.000	0.000	0.000	0.000	0.000	0.000	0.000	0.000	0.000	0.000	0.000	0.000	0.000	0.000	0.000	0.000	0.000	0.000	0.000	0.000	0.000	0.000	0.000	0.000	0.000	0.000	0.000	0.000	0.000	0.000	0.000
k__Bacteria;p__Firmicutes;c__Clostridia;o__Clostridiales;f__[Tissierellaceae];g__Anaerococcus	0.000	0.002	0.000	0.000	0.001	0.063	0.000	0.000	0.000	0.000	0.000	0.003	0.001	0.092	0.000	0.000	0.000	0.000	0.000	0.000	0.000	0.000	0.000	0.000	0.000	0.000	0.000	0.000	0.000	0.000	0.000	0.000	0.000	0.000	0.000	0.000	0.000	0.000	0.000	0.000	0.000	0.000	0.000	0.000	0.022	0.000	0.000	0.000	0.000	0.000	0.000	0.000	0.000	0.000	0.000	0.000	0.000	0.002	0.000	0.000
k__Bacteria;p__Firmicutes;c__Clostridia;o__Clostridiales;f__[Tissierellaceae];g__Finegoldia	0.000	0.005	0.000	0.000	0.000	0.044	0.009	0.000	0.000	0.005	0.001	0.001	0.004	0.028	0.000	0.000	0.000	0.000	0.000	0.000	0.000	0.000	0.000	0.000	0.000	0.000	0.000	0.000	0.000	0.000	0.028	0.000	0.000	0.000	0.000	0.000	0.000	0.000	0.000	0.000	0.000	0.001	0.001	0.001	0.001	0.000	0.000	0.000	0.000	0.001	0.000	0.000	0.000	0.000	0.000	0.027	0.000	0.022	0.000	0.000
k__Bacteria;p__Firmicutes;c__Clostridia;o__Clostridiales;f__[Tissierellaceae];g__Helcococcus	0.000	0.000	0.000	0.000	0.000	0.000	0.000	0.000	0.000	0.000	0.000	0.000	0.000	0.000	0.000	0.000	0.000	0.000	0.000	0.000	0.000	0.000	0.000	0.000	0.000	0.000	0.000	0.000	0.000	0.000	0.000	0.000	0.000	0.000	0.000	0.000	0.000	0.000	0.000	0.000	0.000	0.000	0.000	0.000	0.000	0.000	0.000	0.000	0.000	0.000	0.000	0.000	0.000	0.000	0.000	0.000	0.000	0.000	0.000	0.000
k__Bacteria;p__Firmicutes;c__Clostridia;o__Clostridiales;f__[Tissierellaceae];g__Parvimonas	0.000	0.000	0.000	0.000	0.000	0.010	0.000	0.000	0.002	0.000	0.000	0.000	0.000	0.000	0.000	0.000	0.000	0.000	0.000	0.000	0.000	0.000	0.000	0.000	0.000	0.000	0.000	0.000	0.000	0.000	0.000	0.000	0.000	0.000	0.000	0.000	0.000	0.000	0.000	0.000	0.000	0.000	0.000	0.000	0.000	0.000	0.000	0.000	0.000	0.000	0.000	0.000	0.000	0.000	0.000	0.000	0.000	0.000	0.000	0.000
k__Bacteria;p__Firmicutes;c__Clostridia;o__Clostridiales;f__[Tissierellaceae];g__Peptoniphilus	0.000	0.004	0.000	0.000	0.003	0.140	0.000	0.000	0.000	0.003	0.001	0.004	0.005	0.080	0.000	0.000	0.000	0.000	0.000	0.000	0.000	0.000	0.000	0.000	0.000	0.000	0.000	0.000	0.000	0.000	0.000	0.000	0.000	0.004	0.103	0.000	0.000	0.000	0.134	0.027	0.071	0.000	0.000	0.026	0.004	0.000	0.000	0.000	0.000	0.006	0.000	0.000	0.000	0.000	0.000	0.011	0.000	0.000	0.000	0.000
k__Bacteria;p__Firmicutes;c__Clostridia;o__Clostridiales;f__[Tissierellaceae];g__ph2	0.000	0.000	0.000	0.000	0.000	0.001	0.000	0.000	0.000	0.000	0.000	0.000	0.000	0.001	0.000	0.000	0.000	0.000	0.000	0.000	0.000	0.000	0.000	0.000	0.000	0.000	0.000	0.000	0.000	0.000	0.000	0.000	0.000	0.000	0.000	0.000	0.000	0.000	0.000	0.000	0.000	0.000	0.000	0.000	0.000	0.000	0.000	0.000	0.000	0.000	0.000	0.000	0.000	0.000	0.000	0.000	0.000	0.000	0.000	0.000
k__Bacteria;p__Firmicutes;c__Clostridia;o__Clostridiales;f__[Tissierellaceae];g__WAL_1855D	0.000	0.001	0.000	0.000	0.000	0.198	0.000	0.000	0.000	0.000	0.001	0.000	0.000	0.008	0.000	0.000	0.000	0.000	0.000	0.000	0.000	0.000	0.000	0.000	0.000	0.000	0.000	0.000	0.000	0.000	0.000	0.000	0.000	0.000	0.000	0.000	0.000	0.000	0.000	0.000	0.000	0.000	0.000	0.000	0.000	0.000	0.000	0.000	0.000	0.000	0.000	0.000	0.000	0.000	0.000	0.000	0.000	0.000	0.000	0.000
k__Bacteria;p__Firmicutes;c__Clostridia;o__Clostridiales;f__Christensenellaceae;g__	0.000	0.000	0.000	0.000	0.000	0.000	0.000	0.000	0.000	0.000	0.000	0.000	0.000	0.000	0.000	0.000	0.000	0.000	0.000	0.000	0.000	0.000	0.000	0.000	0.000	0.000	0.000	0.000	0.000	0.000	0.000	0.000	0.000	0.000	0.000	0.000	0.000	0.000	0.000	0.000	0.000	0.000	0.000	0.000	0.000	0.000	0.000	0.000	0.000	0.000	0.000	0.000	0.000	0.000	0.000	0.000	0.000	0.000	0.000	0.000
k__Bacteria;p__Firmicutes;c__Clostridia;o__Clostridiales;f__Christensenellaceae;g__Christensenella	0.000	0.000	0.000	0.000	0.000	0.000	0.000	0.000	0.000	0.000	0.000	0.000	0.000	0.000	0.000	0.000	0.000	0.000	0.000	0.000	0.000	0.000	0.000	0.000	0.000	0.000	0.000	0.000	0.000	0.000	0.000	0.000	0.000	0.000	0.000	0.000	0.000	0.000	0.000	0.000	0.000	0.000	0.000	0.000	0.000	0.000	0.000	0.000	0.000	0.000	0.000	0.000	0.000	0.000	0.000	0.000	0.000	0.000	0.000	0.000
k__Bacteria;p__Firmicutes;c__Clostridia;o__Clostridiales;f__Clostridiaceae;g__	0.000	0.000	0.000	0.000	0.000	0.000	0.002	0.000	0.000	0.001	0.000	0.004	0.001	0.000	0.000	0.000	0.000	0.000	0.000	0.000	0.000	0.000	0.000	0.000	0.000	0.000	0.000	0.000	0.000	0.000	0.000	0.000	0.000	0.000	0.000	0.000	0.000	0.000	0.000	0.000	0.000	0.000	0.000	0.000	0.000	0.000	0.000	0.000	0.001	0.000	0.000	0.000	0.000	0.000	0.000	0.000	0.000	0.000	0.000	0.000
k__Bacteria;p__Firmicutes;c__Clostridia;o__Clostridiales;f__Clostridiaceae;g__Clostridium	0.000	0.001	0.000	0.000	0.000	0.000	0.011	0.000	0.031	0.001	0.001	0.001	0.001	0.013	0.000	0.000	0.000	0.000	0.000	0.000	0.000	0.000	0.000	0.000	0.000	0.000	0.000	0.000	0.000	0.000	0.000	0.000	0.000	0.000	0.000	0.000	0.000	0.000	0.000	0.000	0.000	0.000	0.000	0.000	0.000	0.000	0.000	0.000	0.408	0.000	0.000	0.000	0.000	0.000	0.000	0.000	0.000	0.000	0.017	0.000
k__Bacteria;p__Firmicutes;c__Clostridia;o__Clostridiales;f__Clostridiaceae;g__SMB53	0.000	0.000	0.000	0.000	0.000	0.000	0.000	0.000	0.000	0.000	0.000	0.000	0.000	0.000	0.000	0.000	0.000	0.000	0.000	0.000	0.000	0.000	0.000	0.000	0.000	0.000	0.000	0.000	0.000	0.000	0.000	0.000	0.000	0.000	0.000	0.000	0.000	0.000	0.000	0.000	0.000	0.000	0.000	0.000	0.000	0.000	0.000	0.000	0.000	0.000	0.000	0.000	0.000	0.000	0.000	0.000	0.000	0.000	0.000	0.000
k__Bacteria;p__Firmicutes;c__Clostridia;o__Clostridiales;f__Clostridiaceae;Other	0.000	0.000	0.000	0.000	0.000	0.000	0.000	0.000	0.000	0.000	0.000	0.000	0.000	0.000	0.000	0.000	0.000	0.000	0.000	0.000	0.000	0.000	0.000	0.000	0.000	0.000	0.000	0.000	0.000	0.000	0.000	0.000	0.000	0.000	0.000	0.000	0.000	0.000	0.000	0.000	0.000	0.000	0.000	0.000	0.000	0.000	0.000	0.000	0.000	0.000	0.000	0.000	0.000	0.000	0.000	0.000	0.000	0.000	0.000	0.000
k__Bacteria;p__Firmicutes;c__Clostridia;o__Clostridiales;f__Dehalobacteriaceae;g__Dehalobacterium	0.000	0.000	0.000	0.000	0.000	0.000	0.000	0.000	0.000	0.000	0.000	0.000	0.000	0.000	0.000	0.000	0.000	0.000	0.000	0.000	0.000	0.000	0.000	0.000	0.000	0.000	0.000	0.000	0.000	0.000	0.000	0.000	0.000	0.000	0.000	0.000	0.000	0.000	0.000	0.000	0.000	0.000	0.000	0.000	0.000	0.000	0.000	0.000	0.000	0.000	0.000	0.000	0.000	0.000	0.000	0.000	0.000	0.000	0.000	0.000
k__Bacteria;p__Firmicutes;c__Clostridia;o__Clostridiales;f__Eubacteriaceae;g__Anaerofustis	0.000	0.000	0.000	0.000	0.000	0.000	0.000	0.000	0.000	0.000	0.000	0.000	0.000	0.000	0.000	0.000	0.000	0.000	0.000	0.000	0.000	0.000	0.000	0.000	0.000	0.000	0.000	0.000	0.000	0.000	0.000	0.000	0.000	0.000	0.000	0.000	0.000	0.000	0.000	0.000	0.000	0.000	0.000	0.000	0.000	0.000	0.000	0.000	0.000	0.000	0.000	0.000	0.000	0.000	0.000	0.000	0.000	0.000	0.000	0.000
k__Bacteria;p__Firmicutes;c__Clostridia;o__Clostridiales;f__Eubacteriaceae;g__Pseudoramibacter_Eubacterium	0.000	0.000	0.000	0.000	0.000	0.000	0.000	0.000	0.000	0.000	0.000	0.000	0.000	0.000	0.000	0.000	0.000	0.000	0.000	0.000	0.000	0.000	0.000	0.000	0.000	0.000	0.000	0.000	0.000	0.000	0.000	0.000	0.000	0.000	0.000	0.000	0.000	0.000	0.000	0.000	0.000	0.000	0.000	0.000	0.000	0.000	0.017	0.000	0.000	0.000	0.000	0.000	0.000	0.000	0.000	0.000	0.000	0.000	0.000	0.000
k__Bacteria;p__Firmicutes;c__Clostridia;o__Clostridiales;f__Lachnospiraceae;g__	0.000	0.002	0.001	0.001	0.000	0.015	0.019	0.000	0.000	0.014	0.000	0.007	0.027	0.009	0.001	0.000	0.000	0.000	0.000	0.000	0.000	0.000	0.000	0.000	0.000	0.000	0.000	0.000	0.000	0.000	0.000	0.000	0.000	0.000	0.000	0.000	0.000	0.000	0.000	0.000	0.000	0.000	0.001	0.001	0.000	0.000	0.000	0.000	0.000	0.001	0.000	0.000	0.000	0.000	0.000	0.000	0.000	0.000	0.000	0.000
k__Bacteria;p__Firmicutes;c__Clostridia;o__Clostridiales;f__Lachnospiraceae;g__[Ruminococcus]	0.000	0.001	0.000	0.000	0.000	0.003	0.001	0.000	0.000	0.001	0.000	0.002	0.001	0.000	0.000	0.000	0.000	0.000	0.000	0.000	0.000	0.000	0.000	0.000	0.000	0.000	0.000	0.000	0.000	0.000	0.000	0.000	0.000	0.000	0.000	0.000	0.000	0.000	0.000	0.000	0.000	0.000	0.000	0.000	0.000	0.000	0.000	0.000	0.000	0.000	0.000	0.000	0.000	0.000	0.000	0.000	0.000	0.000	0.000	0.045
k__Bacteria;p__Firmicutes;c__Clostridia;o__Clostridiales;f__Lachnospiraceae;g__Anaerostipes	0.000	0.000	0.000	0.000	0.000	0.000	0.000	0.000	0.000	0.000	0.000	0.000	0.000	0.001	0.000	0.000	0.000	0.000	0.000	0.000	0.000	0.000	0.000	0.000	0.000	0.000	0.000	0.000	0.000	0.000	0.000	0.000	0.000	0.000	0.000	0.000	0.000	0.000	0.000	0.000	0.000	0.000	0.000	0.000	0.000	0.000	0.000	0.000	0.001	0.000	0.000	0.000	0.000	0.000	0.000	0.000	0.000	0.000	0.000	0.000
k__Bacteria;p__Firmicutes;c__Clostridia;o__Clostridiales;f__Lachnospiraceae;g__Blautia	0.001	0.002	0.000	0.000	0.000	0.004	0.015	0.000	0.000	0.003	0.000	0.004	0.005	0.005	0.000	0.000	0.000	0.000	0.000	0.000	0.000	0.000	0.000	0.000	0.000	0.000	0.000	0.000	0.000	0.000	0.000	0.000	0.000	0.000	0.000	0.000	0.000	0.000	0.000	0.000	0.000	0.000	0.000	0.000	0.000	0.000	0.000	0.000	0.000	0.000	0.000	0.000	0.000	0.000	0.000	0.000	0.000	0.000	0.000	0.000
k__Bacteria;p__Firmicutes;c__Clostridia;o__Clostridiales;f__Lachnospiraceae;g__Coprococcus	0.000	0.000	0.000	0.000	0.000	0.003	0.003	0.000	0.000	0.001	0.000	0.001	0.002	0.001	0.000	0.000	0.000	0.000	0.000	0.000	0.000	0.000	0.000	0.000	0.000	0.000	0.000	0.000	0.000	0.000	0.000	0.000	0.000	0.000	0.000	0.000	0.000	0.000	0.000	0.000	0.000	0.000	0.000	0.000	0.000	0.000	0.000	0.000	0.000	0.000	0.000	0.000	0.000	0.000	0.000	0.000	0.000	0.000	0.000	0.000
k__Bacteria;p__Firmicutes;c__Clostridia;o__Clostridiales;f__Lachnospiraceae;g__Dorea	0.000	0.000	0.000	0.000	0.000	0.000	0.004	0.000	0.000	0.000	0.000	0.001	0.001	0.001	0.000	0.000	0.000	0.000	0.000	0.000	0.000	0.000	0.000	0.000	0.000	0.000	0.000	0.000	0.000	0.000	0.000	0.000	0.000	0.000	0.000	0.000	0.000	0.000	0.000	0.000	0.000	0.000	0.000	0.000	0.000	0.000	0.000	0.000	0.000	0.000	0.000	0.000	0.000	0.000	0.000	0.000	0.000	0.000	0.000	0.000
k__Bacteria;p__Firmicutes;c__Clostridia;o__Clostridiales;f__Lachnospiraceae;g__Lachnobacterium	0.000	0.000	0.000	0.000	0.000	0.000	0.000	0.000	0.000	0.000	0.000	0.000	0.000	0.000	0.000	0.000	0.000	0.000	0.000	0.000	0.000	0.000	0.000	0.000	0.000	0.000	0.000	0.000	0.000	0.000	0.000	0.000	0.000	0.000	0.000	0.000	0.000	0.000	0.000	0.000	0.000	0.000	0.000	0.000	0.000	0.000	0.000	0.000	0.000	0.000	0.000	0.000	0.000	0.000	0.000	0.000	0.000	0.000	0.000	0.000
k__Bacteria;p__Firmicutes;c__Clostridia;o__Clostridiales;f__Lachnospiraceae;g__Lachnospira	0.000	0.000	0.000	0.000	0.000	0.003	0.001	0.000	0.000	0.001	0.000	0.000	0.000	0.000	0.000	0.000	0.000	0.000	0.000	0.000	0.000	0.000	0.000	0.000	0.000	0.000	0.000	0.000	0.000	0.000	0.000	0.000	0.000	0.000	0.000	0.000	0.000	0.000	0.000	0.000	0.000	0.000	0.000	0.000	0.000	0.000	0.000	0.000	0.000	0.000	0.000	0.000	0.000	0.000	0.000	0.000	0.000	0.000	0.000	0.000
k__Bacteria;p__Firmicutes;c__Clostridia;o__Clostridiales;f__Lachnospiraceae;g__Roseburia	0.000	0.000	0.000	0.000	0.000	0.000	0.000	0.000	0.000	0.000	0.000	0.000	0.000	0.000	0.000	0.000	0.000	0.000	0.000	0.000	0.000	0.000	0.000	0.000	0.000	0.000	0.000	0.000	0.000	0.000	0.000	0.000	0.000	0.000	0.000	0.000	0.000	0.000	0.000	0.000	0.000	0.000	0.000	0.000	0.000	0.000	0.000	0.000	0.000	0.000	0.000	0.000	0.000	0.000	0.000	0.000	0.000	0.000	0.000	0.000
k__Bacteria;p__Firmicutes;c__Clostridia;o__Clostridiales;f__Lachnospiraceae;Other	0.000	0.000	0.000	0.000	0.000	0.001	0.001	0.000	0.000	0.000	0.000	0.000	0.001	0.000	0.000	0.000	0.000	0.000	0.000	0.000	0.000	0.000	0.000	0.000	0.000	0.000	0.000	0.000	0.000	0.000	0.000	0.000	0.000	0.000	0.000	0.000	0.000	0.000	0.000	0.000	0.000	0.000	0.000	0.000	0.000	0.000	0.000	0.000	0.000	0.000	0.000	0.000	0.000	0.000	0.000	0.000	0.000	0.000	0.000	0.000
k__Bacteria;p__Firmicutes;c__Clostridia;o__Clostridiales;f__Peptococcaceae;g__	0.000	0.000	0.000	0.000	0.000	0.000	0.000	0.000	0.000	0.000	0.000	0.000	0.000	0.000	0.000	0.000	0.000	0.000	0.000	0.000	0.000	0.000	0.000	0.000	0.000	0.000	0.000	0.000	0.000	0.000	0.000	0.000	0.000	0.000	0.000	0.000	0.000	0.000	0.000	0.000	0.000	0.000	0.000	0.000	0.000	0.000	0.000	0.000	0.000	0.000	0.000	0.000	0.000	0.000	0.000	0.000	0.000	0.000	0.000	0.000
k__Bacteria;p__Firmicutes;c__Clostridia;o__Clostridiales;f__Peptococcaceae;g__Peptococcus	0.000	0.000	0.000	0.000	0.000	0.000	0.000	0.000	0.000	0.000	0.000	0.000	0.000	0.000	0.000	0.000	0.000	0.000	0.000	0.000	0.000	0.000	0.000	0.000	0.000	0.000	0.000	0.000	0.000	0.000	0.000	0.000	0.000	0.000	0.000	0.000	0.000	0.000	0.000	0.000	0.000	0.000	0.000	0.000	0.000	0.000	0.000	0.000	0.000	0.000	0.000	0.000	0.000	0.000	0.000	0.000	0.000	0.000	0.000	0.000
k__Bacteria;p__Firmicutes;c__Clostridia;o__Clostridiales;f__Peptococcaceae;g__rc4-4	0.000	0.000	0.000	0.000	0.000	0.000	0.000	0.000	0.000	0.000	0.000	0.000	0.000	0.000	0.000	0.000	0.000	0.000	0.000	0.000	0.000	0.000	0.000	0.000	0.000	0.000	0.000	0.000	0.000	0.000	0.000	0.000	0.000	0.000	0.000	0.000	0.000	0.000	0.000	0.000	0.000	0.000	0.000	0.000	0.000	0.000	0.000	0.000	0.000	0.000	0.000	0.000	0.000	0.000	0.000	0.000	0.000	0.000	0.000	0.000
k__Bacteria;p__Firmicutes;c__Clostridia;o__Clostridiales;f__Peptostreptococcaceae;g__	0.000	0.000	0.000	0.000	0.000	0.000	0.000	0.000	0.000	0.000	0.000	0.000	0.000	0.000	0.000	0.000	0.000	0.000	0.000	0.000	0.000	0.000	0.000	0.000	0.000	0.000	0.000	0.000	0.000	0.000	0.000	0.000	0.000	0.000	0.000	0.000	0.000	0.000	0.000	0.000	0.000	0.000	0.000	0.000	0.000	0.000	0.000	0.000	0.000	0.000	0.000	0.000	0.000	0.000	0.000	0.000	0.000	0.000	0.000	0.000
k__Bacteria;p__Firmicutes;c__Clostridia;o__Clostridiales;f__Peptostreptococcaceae;g__Peptostreptococcus	0.000	0.000	0.000	0.000	0.000	0.003	0.000	0.000	0.000	0.000	0.000	0.003	0.000	0.000	0.000	0.000	0.000	0.000	0.000	0.000	0.000	0.000	0.000	0.000	0.000	0.000	0.000	0.000	0.000	0.000	0.000	0.000	0.000	0.000	0.001	0.000	0.000	0.000	0.000	0.000	0.052	0.000	0.002	0.007	0.000	0.000	0.001	0.000	0.000	0.000	0.000	0.000	0.000	0.000	0.000	0.000	0.000	0.000	0.000	0.000
k__Bacteria;p__Firmicutes;c__Clostridia;o__Clostridiales;f__Ruminococcaceae;g__	0.000	0.001	0.000	0.000	0.000	0.012	0.005	0.000	0.000	0.003	0.001	0.007	0.004	0.004	0.000	0.000	0.000	0.000	0.000	0.000	0.000	0.000	0.000	0.000	0.000	0.000	0.000	0.000	0.000	0.000	0.000	0.000	0.000	0.000	0.000	0.000	0.000	0.000	0.000	0.000	0.000	0.000	0.000	0.000	0.000	0.000	0.000	0.000	0.000	0.000	0.000	0.000	0.000	0.000	0.000	0.000	0.000	0.000	0.000	0.000
k__Bacteria;p__Firmicutes;c__Clostridia;o__Clostridiales;f__Ruminococcaceae;g__Anaerotruncus	0.000	0.000	0.000	0.000	0.000	0.000	0.000	0.000	0.000	0.000	0.000	0.000	0.000	0.000	0.000	0.000	0.000	0.000	0.000	0.000	0.000	0.000	0.000	0.000	0.000	0.000	0.000	0.000	0.000	0.000	0.000	0.000	0.000	0.000	0.000	0.000	0.000	0.000	0.000	0.000	0.000	0.000	0.000	0.000	0.000	0.000	0.000	0.000	0.000	0.000	0.001	0.001	0.000	0.000	0.000	0.000	0.000	0.000	0.000	0.000
k__Bacteria;p__Firmicutes;c__Clostridia;o__Clostridiales;f__Ruminococcaceae;g__Faecalibacterium	0.000	0.003	0.000	0.000	0.000	0.016	0.015	0.001	0.000	0.010	0.001	0.011	0.008	0.006	0.001	0.000	0.000	0.000	0.000	0.000	0.000	0.000	0.000	0.000	0.000	0.000	0.000	0.000	0.000	0.000	0.000	0.000	0.000	0.000	0.000	0.000	0.000	0.000	0.000	0.000	0.000	0.000	0.000	0.003	0.000	0.000	0.000	0.000	0.000	0.000	0.000	0.000	0.000	0.000	0.000	0.000	0.000	0.002	0.003	0.000
k__Bacteria;p__Firmicutes;c__Clostridia;o__Clostridiales;f__Ruminococcaceae;g__Oscillospira	0.000	0.000	0.000	0.000	0.000	0.003	0.000	0.000	0.000	0.001	0.000	0.001	0.002	0.000	0.000	0.000	0.000	0.000	0.000	0.000	0.000	0.000	0.000	0.000	0.000	0.000	0.000	0.000	0.000	0.000	0.000	0.000	0.000	0.000	0.000	0.000	0.000	0.000	0.000	0.000	0.000	0.000	0.000	0.000	0.000	0.000	0.000	0.000	0.000	0.000	0.000	0.000	0.000	0.000	0.000	0.000	0.000	0.000	0.000	0.000
k__Bacteria;p__Firmicutes;c__Clostridia;o__Clostridiales;f__Ruminococcaceae;g__Ruminococcus	0.000	0.001	0.000	0.000	0.000	0.005	0.000	0.000	0.000	0.003	0.000	0.007	0.002	0.002	0.000	0.000	0.000	0.000	0.000	0.000	0.000	0.000	0.000	0.000	0.000	0.000	0.000	0.000	0.000	0.000	0.000	0.000	0.000	0.000	0.000	0.000	0.000	0.000	0.000	0.000	0.000	0.000	0.000	0.000	0.000	0.000	0.000	0.000	0.000	0.000	0.000	0.000	0.000	0.000	0.000	0.000	0.000	0.000	0.000	0.000
k__Bacteria;p__Firmicutes;c__Clostridia;o__Clostridiales;f__Ruminococcaceae;Other	0.000	0.000	0.000	0.000	0.000	0.001	0.000	0.000	0.000	0.000	0.000	0.001	0.000	0.001	0.000	0.000	0.000	0.000	0.000	0.000	0.000	0.000	0.000	0.000	0.000	0.000	0.000	0.000	0.000	0.000	0.000	0.000	0.000	0.000	0.000	0.000	0.000	0.000	0.000	0.000	0.000	0.000	0.000	0.000	0.000	0.000	0.000	0.000	0.000	0.000	0.000	0.000	0.000	0.000	0.000	0.000	0.000	0.000	0.000	0.000
k__Bacteria;p__Firmicutes;c__Clostridia;o__Clostridiales;f__Veillonellaceae;g__	0.000	0.000	0.000	0.000	0.000	0.000	0.013	0.000	0.000	0.000	0.000	0.001	0.000	0.000	0.000	0.000	0.000	0.000	0.000	0.000	0.000	0.000	0.000	0.000	0.000	0.000	0.000	0.000	0.000	0.000	0.000	0.000	0.000	0.000	0.000	0.000	0.048	0.000	0.000	0.000	0.000	0.000	0.000	0.020	0.000	0.000	0.000	0.000	0.000	0.000	0.000	0.000	0.000	0.000	0.000	0.000	0.000	0.000	0.000	0.000
k__Bacteria;p__Firmicutes;c__Clostridia;o__Clostridiales;f__Veillonellaceae;g__Acidaminococcus	0.000	0.000	0.000	0.000	0.000	0.000	0.000	0.000	0.000	0.001	0.000	0.000	0.000	0.001	0.000	0.000	0.000	0.000	0.000	0.000	0.000	0.000	0.000	0.000	0.000	0.000	0.000	0.000	0.000	0.000	0.000	0.000	0.000	0.000	0.000	0.000	0.000	0.005	0.002	0.006	0.000	0.000	0.000	0.007	0.000	0.000	0.000	0.000	0.000	0.000	0.083	0.000	0.000	0.000	0.003	0.000	0.000	0.000	0.000	0.000
k__Bacteria;p__Firmicutes;c__Clostridia;o__Clostridiales;f__Veillonellaceae;g__Dialister	0.000	0.002	0.000	0.000	0.000	0.012	0.001	0.000	0.004	0.001	0.001	0.001	0.000	0.008	0.000	0.000	0.000	0.000	0.000	0.000	0.000	0.000	0.000	0.000	0.000	0.000	0.000	0.000	0.000	0.000	0.000	0.000	0.000	0.000	0.000	0.000	0.045	0.000	0.000	0.000	0.000	0.000	0.000	0.000	0.000	0.000	0.000	0.000	0.000	0.000	0.000	0.000	0.000	0.000	0.000	0.000	0.000	0.000	0.000	0.000
k__Bacteria;p__Firmicutes;c__Clostridia;o__Clostridiales;f__Veillonellaceae;g__Megamonas	0.000	0.000	0.000	0.000	0.000	0.006	0.009	0.000	0.000	0.006	0.000	0.003	0.004	0.004	0.000	0.000	0.000	0.000	0.000	0.000	0.000	0.000	0.000	0.000	0.000	0.000	0.000	0.000	0.000	0.000	0.000	0.000	0.000	0.000	0.000	0.000	0.000	0.000	0.000	0.000	0.000	0.000	0.000	0.000	0.000	0.000	0.000	0.000	0.000	0.000	0.000	0.000	0.000	0.000	0.000	0.000	0.000	0.000	0.000	0.000
k__Bacteria;p__Firmicutes;c__Clostridia;o__Clostridiales;f__Veillonellaceae;g__Megasphaera	0.000	0.000	0.000	0.000	0.000	0.000	0.001	0.079	0.135	0.002	0.000	0.000	0.002	0.001	0.000	0.000	0.000	0.000	0.000	0.000	0.000	0.000	0.000	0.000	0.000	0.000	0.000	0.000	0.000	0.000	0.000	0.000	0.000	0.000	0.000	0.000	0.000	0.000	0.000	0.000	0.000	0.000	0.000	0.000	0.000	0.000	0.000	0.000	0.000	0.000	0.000	0.000	0.000	0.000	0.000	0.000	0.000	0.000	0.000	0.000
k__Bacteria;p__Firmicutes;c__Clostridia;o__Clostridiales;f__Veillonellaceae;g__Mitsuokella	0.000	0.000	0.000	0.000	0.000	0.000	0.001	0.000	0.000	0.000	0.000	0.000	0.000	0.000	0.000	0.000	0.000	0.000	0.000	0.000	0.000	0.000	0.000	0.000	0.000	0.000	0.000	0.000	0.000	0.000	0.000	0.000	0.000	0.000	0.000	0.000	0.000	0.000	0.000	0.000	0.000	0.000	0.000	0.000	0.000	0.000	0.000	0.000	0.000	0.000	0.000	0.000	0.000	0.000	0.000	0.000	0.000	0.000	0.000	0.000
k__Bacteria;p__Firmicutes;c__Clostridia;o__Clostridiales;f__Veillonellaceae;g__Phascolarctobacterium	0.000	0.000	0.000	0.000	0.000	0.000	0.001	0.000	0.000	0.000	0.000	0.000	0.000	0.000	0.000	0.000	0.000	0.000	0.000	0.000	0.000	0.000	0.000	0.000	0.000	0.000	0.000	0.000	0.000	0.000	0.000	0.000	0.000	0.000	0.000	0.000	0.000	0.000	0.000	0.000	0.000	0.000	0.000	0.000	0.000	0.000	0.000	0.000	0.000	0.000	0.000	0.000	0.000	0.000	0.000	0.000	0.000	0.000	0.000	0.000
k__Bacteria;p__Firmicutes;c__Clostridia;o__Clostridiales;f__Veillonellaceae;g__Succiniclasticum	0.000	0.000	0.000	0.000	0.000	0.004	0.000	0.000	0.000	0.000	0.000	0.000	0.000	0.000	0.000	0.000	0.000	0.000	0.000	0.000	0.000	0.000	0.000	0.000	0.000	0.000	0.000	0.000	0.000	0.000	0.000	0.000	0.000	0.000	0.000	0.000	0.000	0.000	0.000	0.047	0.000	0.000	0.001	0.054	0.000	0.000	0.000	0.000	0.005	0.006	0.000	0.003	0.001	0.000	0.000	0.000	0.000	0.002	0.000	0.000
k__Bacteria;p__Firmicutes;c__Clostridia;o__Clostridiales;f__Veillonellaceae;g__Veillonella	0.000	0.000	0.000	0.000	0.000	0.000	0.002	0.000	0.000	0.000	0.000	0.000	0.000	0.000	0.000	0.000	0.000	0.000	0.000	0.000	0.000	0.000	0.000	0.000	0.000	0.000	0.000	0.000	0.000	0.000	0.000	0.000	0.000	0.000	0.000	0.000	0.000	0.000	0.000	0.000	0.000	0.000	0.000	0.000	0.000	0.000	0.000	0.000	0.000	0.000	0.000	0.000	0.000	0.000	0.000	0.000	0.000	0.000	0.000	0.000
k__Bacteria;p__Firmicutes;c__Clostridia;o__Clostridiales;f__Veillonellaceae;Other	0.000	0.000	0.000	0.000	0.000	0.000	0.000	0.000	0.000	0.000	0.000	0.001	0.000	0.001	0.000	0.000	0.000	0.000	0.000	0.000	0.000	0.000	0.000	0.000	0.000	0.000	0.000	0.000	0.000	0.000	0.000	0.000	0.000	0.000	0.000	0.000	0.000	0.000	0.000	0.000	0.000	0.000	0.000	0.000	0.000	0.000	0.000	0.000	0.000	0.000	0.000	0.000	0.000	0.000	0.000	0.000	0.000	0.000	0.000	0.000
k__Bacteria;p__Firmicutes;c__Clostridia;o__Clostridiales;Other;Other	0.000	0.000	0.000	0.000	0.000	0.000	0.007	0.000	0.000	0.000	0.000	0.001	0.001	0.000	0.000	0.000	0.000	0.000	0.000	0.000	0.000	0.000	0.000	0.000	0.000	0.000	0.000	0.000	0.000	0.000	0.000	0.000	0.000	0.000	0.000	0.000	0.000	0.000	0.000	0.000	0.000	0.000	0.000	0.000	0.000	0.000	0.000	0.000	0.000	0.000	0.000	0.000	0.000	0.000	0.000	0.000	0.000	0.000	0.000	0.000
k__Bacteria;p__Firmicutes;c__Erysipelotrichi;o__Erysipelotrichales;f__Erysipelotrichaceae;g__	0.000	0.001	0.001	0.000	0.000	0.000	0.001	0.000	0.000	0.005	0.000	0.001	0.011	0.006	0.000	0.000	0.000	0.000	0.000	0.000	0.000	0.000	0.000	0.000	0.000	0.000	0.000	0.000	0.018	0.000	0.000	0.000	0.000	0.000	0.000	0.000	0.005	0.000	0.000	0.000	0.000	0.000	0.000	0.258	0.000	0.000	0.000	0.000	0.331	0.000	0.000	0.000	0.000	0.000	0.000	0.000	0.000	0.000	0.000	0.000
k__Bacteria;p__Firmicutes;c__Erysipelotrichi;o__Erysipelotrichales;f__Erysipelotrichaceae;g__[Eubacterium]	0.000	0.000	0.000	0.000	0.000	0.000	0.005	0.000	0.000	0.000	0.000	0.000	0.000	0.000	0.000	0.000	0.000	0.000	0.000	0.000	0.000	0.000	0.000	0.000	0.000	0.000	0.000	0.000	0.000	0.000	0.000	0.000	0.000	0.000	0.000	0.000	0.000	0.000	0.000	0.000	0.000	0.000	0.602	0.002	0.000	0.000	0.003	0.000	0.000	0.000	0.000	0.000	0.000	0.000	0.000	0.000	0.000	0.000	0.000	0.000
k__Bacteria;p__Firmicutes;c__Erysipelotrichi;o__Erysipelotrichales;f__Erysipelotrichaceae;g__Allobaculum	0.000	0.000	0.000	0.000	0.000	0.000	0.000	0.000	0.000	0.000	0.000	0.000	0.000	0.000	0.000	0.000	0.000	0.000	0.000	0.000	0.000	0.000	0.000	0.000	0.000	0.000	0.000	0.000	0.000	0.000	0.000	0.000	0.000	0.000	0.000	0.000	0.000	0.000	0.000	0.000	0.000	0.000	0.000	0.000	0.000	0.000	0.000	0.000	0.000	0.000	0.000	0.000	0.000	0.000	0.000	0.000	0.000	0.000	0.000	0.000
k__Bacteria;p__Firmicutes;c__Erysipelotrichi;o__Erysipelotrichales;f__Erysipelotrichaceae;g__Bulleidia	0.000	0.000	0.000	0.000	0.000	0.000	0.000	0.000	0.000	0.000	0.000	0.000	0.000	0.000	0.000	0.000	0.000	0.000	0.000	0.000	0.000	0.000	0.000	0.000	0.000	0.000	0.000	0.000	0.000	0.000	0.000	0.000	0.000	0.000	0.000	0.000	0.000	0.000	0.000	0.001	0.000	0.000	0.000	0.000	0.000	0.000	0.000	0.000	0.000	0.000	0.000	0.000	0.000	0.000	0.000	0.000	0.000	0.000	0.000	0.000
k__Bacteria;p__Firmicutes;c__Erysipelotrichi;o__Erysipelotrichales;f__Erysipelotrichaceae;g__Catenibacterium	0.000	0.000	0.000	0.000	0.000	0.000	0.000	0.000	0.000	0.000	0.000	0.000	0.002	0.000	0.000	0.000	0.000	0.000	0.000	0.000	0.000	0.000	0.000	0.000	0.000	0.000	0.000	0.000	0.000	0.000	0.000	0.000	0.000	0.000	0.000	0.000	0.000	0.000	0.000	0.000	0.000	0.000	0.000	0.000	0.000	0.000	0.000	0.000	0.000	0.000	0.000	0.000	0.000	0.000	0.000	0.000	0.000	0.000	0.000	0.000
k__Bacteria;p__Firmicutes;c__Erysipelotrichi;o__Erysipelotrichales;f__Erysipelotrichaceae;g__cc_115	0.000	0.000	0.000	0.000	0.000	0.000	0.000	0.000	0.000	0.000	0.000	0.000	0.000	0.000	0.000	0.000	0.000	0.000	0.000	0.000	0.000	0.000	0.000	0.000	0.000	0.000	0.000	0.000	0.000	0.000	0.000	0.000	0.000	0.000	0.000	0.000	0.000	0.000	0.000	0.000	0.000	0.000	0.000	0.000	0.000	0.000	0.000	0.000	0.000	0.000	0.000	0.000	0.000	0.000	0.000	0.000	0.000	0.000	0.000	0.000
k__Bacteria;p__Firmicutes;c__Erysipelotrichi;o__Erysipelotrichales;f__Erysipelotrichaceae;g__Clostridium	0.000	0.000	0.000	0.000	0.000	0.000	0.000	0.000	0.000	0.000	0.000	0.000	0.000	0.000	0.000	0.000	0.000	0.000	0.000	0.000	0.000	0.000	0.000	0.000	0.000	0.000	0.000	0.000	0.000	0.000	0.000	0.000	0.000	0.000	0.000	0.000	0.000	0.000	0.000	0.000	0.000	0.000	0.000	0.000	0.000	0.000	0.000	0.000	0.000	0.000	0.000	0.000	0.000	0.000	0.000	0.000	0.000	0.000	0.000	0.000
k__Bacteria;p__Firmicutes;c__Erysipelotrichi;o__Erysipelotrichales;f__Erysipelotrichaceae;g__Coprobacillus	0.000	0.000	0.000	0.000	0.000	0.000	0.000	0.000	0.000	0.000	0.000	0.000	0.000	0.000	0.000	0.000	0.000	0.000	0.000	0.000	0.000	0.000	0.000	0.000	0.000	0.000	0.000	0.000	0.000	0.000	0.000	0.000	0.000	0.000	0.000	0.000	0.000	0.000	0.000	0.000	0.000	0.000	0.000	0.000	0.000	0.000	0.000	0.000	0.000	0.000	0.000	0.000	0.000	0.000	0.000	0.000	0.000	0.000	0.000	0.000
k__Bacteria;p__Firmicutes;c__Erysipelotrichi;o__Erysipelotrichales;f__Erysipelotrichaceae;g__Holdemania	0.000	0.000	0.000	0.000	0.000	0.000	0.000	0.000	0.000	0.000	0.000	0.000	0.000	0.000	0.000	0.000	0.000	0.000	0.000	0.000	0.000	0.000	0.000	0.000	0.000	0.000	0.000	0.000	0.000	0.000	0.000	0.000	0.000	0.000	0.000	0.000	0.000	0.000	0.000	0.000	0.000	0.000	0.000	0.000	0.000	0.000	0.000	0.000	0.000	0.000	0.000	0.000	0.000	0.000	0.000	0.000	0.000	0.000	0.000	0.000
k__Bacteria;p__Fusobacteria;c__Fusobacteriia;o__Fusobacteriales;f__Fusobacteriaceae;g__Fusobacterium	0.000	0.000	0.000	0.000	0.000	0.000	0.000	0.000	0.000	0.000	0.000	0.000	0.000	0.000	0.000	0.000	0.000	0.000	0.000	0.000	0.000	0.000	0.000	0.000	0.000	0.000	0.000	0.000	0.000	0.000	0.000	0.000	0.000	0.000	0.000	0.000	0.000	0.000	0.000	0.000	0.000	0.000	0.000	0.000	0.000	0.000	0.000	0.000	0.000	0.000	0.000	0.000	0.000	0.000	0.000	0.000	0.000	0.000	0.000	0.000
k__Bacteria;p__Fusobacteria;c__Fusobacteriia;o__Fusobacteriales;f__Leptotrichiaceae;g__Leptotrichia	0.000	0.000	0.000	0.000	0.000	0.000	0.000	0.066	0.190	0.000	0.000	0.000	0.000	0.000	0.000	0.000	0.000	0.000	0.000	0.000	0.000	0.000	0.000	0.000	0.000	0.000	0.000	0.000	0.000	0.000	0.000	0.000	0.000	0.000	0.000	0.000	0.000	0.000	0.000	0.000	0.000	0.000	0.000	0.000	0.000	0.000	0.000	0.000	0.000	0.000	0.000	0.000	0.000	0.000	0.000	0.000	0.000	0.000	0.000	0.000
k__Bacteria;p__Fusobacteria;c__Fusobacteriia;o__Fusobacteriales;f__Leptotrichiaceae;g__Sneathia	0.000	0.000	0.000	0.000	0.000	0.000	0.000	0.000	0.000	0.000	0.000	0.000	0.001	0.000	0.000	0.000	0.000	0.000	0.000	0.000	0.000	0.000	0.000	0.000	0.000	0.000	0.000	0.000	0.000	0.000	0.000	0.000	0.000	0.000	0.000	0.000	0.000	0.000	0.000	0.000	0.000	0.000	0.000	0.000	0.000	0.000	0.000	0.000	0.000	0.000	0.000	0.000	0.000	0.000	0.000	0.000	0.000	0.000	0.000	0.000
k__Bacteria;p__Proteobacteria;c__Alphaproteobacteria;o__Caulobacterales;f__Caulobacteraceae;g__	0.000	0.000	0.000	0.000	0.000	0.000	0.000	0.000	0.000	0.000	0.000	0.000	0.000	0.000	0.000	0.000	0.000	0.000	0.000	0.000	0.000	0.000	0.000	0.000	0.000	0.000	0.000	0.000	0.000	0.000	0.000	0.000	0.000	0.000	0.000	0.000	0.000	0.000	0.000	0.000	0.000	0.000	0.000	0.000	0.000	0.000	0.000	0.000	0.000	0.000	0.000	0.000	0.000	0.000	0.000	0.000	0.000	0.000	0.000	0.000
k__Bacteria;p__Proteobacteria;c__Alphaproteobacteria;o__Caulobacterales;f__Caulobacteraceae;g__Asticcacaulis	0.000	0.000	0.000	0.000	0.000	0.000	0.000	0.000	0.000	0.000	0.000	0.000	0.000	0.000	0.000	0.000	0.000	0.000	0.000	0.000	0.000	0.000	0.000	0.000	0.000	0.000	0.000	0.000	0.000	0.000	0.000	0.000	0.000	0.000	0.000	0.000	0.000	0.000	0.000	0.000	0.000	0.000	0.000	0.000	0.000	0.000	0.000	0.000	0.000	0.000	0.000	0.000	0.000	0.000	0.000	0.000	0.000	0.000	0.000	0.000
k__Bacteria;p__Proteobacteria;c__Alphaproteobacteria;o__Caulobacterales;f__Caulobacteraceae;g__Phenylobacterium	0.000	0.000	0.000	0.000	0.000	0.000	0.000	0.000	0.000	0.000	0.000	0.000	0.000	0.000	0.000	0.000	0.000	0.000	0.000	0.000	0.000	0.000	0.000	0.000	0.000	0.000	0.000	0.000	0.000	0.000	0.000	0.000	0.000	0.000	0.000	0.000	0.000	0.000	0.000	0.000	0.000	0.000	0.000	0.000	0.000	0.000	0.000	0.000	0.000	0.000	0.000	0.000	0.000	0.000	0.000	0.000	0.000	0.000	0.000	0.000
k__Bacteria;p__Proteobacteria;c__Alphaproteobacteria;o__Rhizobiales;f__;g__	0.000	0.000	0.000	0.000	0.000	0.000	0.000	0.000	0.000	0.000	0.000	0.000	0.000	0.000	0.000	0.000	0.000	0.000	0.000	0.000	0.000	0.000	0.000	0.000	0.000	0.000	0.000	0.000	0.000	0.000	0.000	0.000	0.000	0.000	0.000	0.000	0.000	0.000	0.000	0.000	0.000	0.000	0.000	0.000	0.000	0.000	0.000	0.000	0.000	0.000	0.000	0.000	0.000	0.000	0.000	0.000	0.000	0.000	0.000	0.000
k__Bacteria;p__Proteobacteria;c__Alphaproteobacteria;o__Rhizobiales;f__Bradyrhizobiaceae;g__	0.000	0.000	0.000	0.000	0.000	0.000	0.000	0.000	0.000	0.000	0.000	0.000	0.000	0.000	0.000	0.000	0.000	0.000	0.000	0.000	0.000	0.000	0.000	0.000	0.000	0.000	0.000	0.000	0.000	0.000	0.000	0.000	0.000	0.000	0.000	0.000	0.000	0.000	0.000	0.000	0.000	0.000	0.000	0.000	0.000	0.000	0.000	0.000	0.000	0.000	0.000	0.000	0.000	0.000	0.000	0.000	0.000	0.000	0.000	0.000
k__Bacteria;p__Proteobacteria;c__Alphaproteobacteria;o__Rhizobiales;f__Bradyrhizobiaceae;g__Bradyrhizobium	0.000	0.000	0.000	0.000	0.000	0.000	0.000	0.000	0.000	0.000	0.000	0.000	0.000	0.000	0.000	0.000	0.000	0.000	0.000	0.000	0.000	0.000	0.000	0.000	0.000	0.000	0.000	0.000	0.000	0.000	0.000	0.000	0.000	0.000	0.000	0.000	0.000	0.000	0.000	0.000	0.000	0.000	0.000	0.000	0.000	0.000	0.000	0.000	0.000	0.000	0.000	0.000	0.000	0.000	0.000	0.000	0.000	0.000	0.000	0.000
k__Bacteria;p__Proteobacteria;c__Alphaproteobacteria;o__Rhizobiales;f__Hyphomicrobiaceae;g__Devosia	0.000	0.000	0.000	0.000	0.000	0.000	0.000	0.000	0.000	0.000	0.000	0.000	0.000	0.000	0.000	0.000	0.000	0.000	0.000	0.000	0.000	0.000	0.000	0.000	0.000	0.000	0.000	0.000	0.000	0.000	0.000	0.000	0.000	0.000	0.000	0.000	0.000	0.000	0.000	0.000	0.000	0.000	0.000	0.000	0.000	0.000	0.000	0.000	0.000	0.000	0.000	0.000	0.000	0.000	0.000	0.000	0.000	0.000	0.000	0.000
k__Bacteria;p__Proteobacteria;c__Alphaproteobacteria;o__Rhizobiales;f__Methylobacteriaceae;g__Methylobacterium	0.000	0.000	0.000	0.000	0.000	0.000	0.000	0.000	0.000	0.000	0.000	0.001	0.000	0.000	0.000	0.000	0.000	0.000	0.000	0.000	0.000	0.000	0.000	0.000	0.000	0.000	0.000	0.000	0.000	0.000	0.000	0.000	0.000	0.000	0.000	0.000	0.000	0.000	0.000	0.000	0.000	0.000	0.000	0.000	0.000	0.000	0.000	0.000	0.000	0.000	0.000	0.000	0.000	0.000	0.000	0.000	0.000	0.000	0.000	0.000
k__Bacteria;p__Proteobacteria;c__Alphaproteobacteria;o__Rhizobiales;f__Rhizobiaceae;g__Agrobacterium	0.000	0.000	0.000	0.000	0.000	0.000	0.000	0.000	0.000	0.000	0.000	0.000	0.006	0.000	0.000	0.000	0.000	0.000	0.000	0.000	0.000	0.000	0.000	0.000	0.000	0.000	0.000	0.000	0.000	0.000	0.000	0.000	0.000	0.000	0.000	0.000	0.000	0.000	0.000	0.000	0.000	0.000	0.000	0.000	0.000	0.000	0.000	0.000	0.000	0.000	0.000	0.000	0.000	0.000	0.000	0.000	0.000	0.000	0.000	0.000
k__Bacteria;p__Proteobacteria;c__Alphaproteobacteria;o__Rhodobacterales;f__Rhodobacteraceae;g__Amaricoccus	0.000	0.000	0.000	0.000	0.000	0.000	0.000	0.000	0.000	0.000	0.000	0.000	0.000	0.000	0.000	0.000	0.000	0.000	0.000	0.000	0.000	0.000	0.000	0.000	0.000	0.000	0.000	0.000	0.000	0.000	0.000	0.000	0.000	0.000	0.000	0.000	0.000	0.000	0.000	0.000	0.000	0.000	0.000	0.000	0.000	0.000	0.000	0.000	0.000	0.000	0.000	0.000	0.000	0.000	0.000	0.000	0.000	0.000	0.000	0.000
k__Bacteria;p__Proteobacteria;c__Alphaproteobacteria;o__Rhodobacterales;f__Rhodobacteraceae;g__Paracoccus	0.000	0.000	0.000	0.000	0.000	0.000	0.000	0.000	0.000	0.000	0.000	0.000	0.000	0.000	0.000	0.000	0.000	0.000	0.000	0.000	0.000	0.000	0.000	0.000	0.000	0.000	0.000	0.000	0.000	0.000	0.000	0.000	0.000	0.000	0.000	0.000	0.000	0.000	0.000	0.000	0.000	0.000	0.000	0.000	0.000	0.000	0.000	0.000	0.000	0.000	0.000	0.000	0.000	0.000	0.000	0.000	0.000	0.000	0.000	0.000
k__Bacteria;p__Proteobacteria;c__Alphaproteobacteria;o__Rhodospirillales;f__Acetobacteraceae;g__Roseomonas	0.000	0.000	0.000	0.000	0.000	0.000	0.000	0.000	0.000	0.000	0.000	0.000	0.000	0.000	0.000	0.000	0.000	0.000	0.000	0.000	0.000	0.000	0.000	0.000	0.000	0.000	0.000	0.000	0.000	0.000	0.000	0.000	0.000	0.000	0.000	0.000	0.000	0.000	0.000	0.000	0.000	0.000	0.000	0.000	0.000	0.000	0.000	0.000	0.000	0.000	0.000	0.000	0.000	0.000	0.000	0.000	0.000	0.000	0.000	0.000
k__Bacteria;p__Proteobacteria;c__Alphaproteobacteria;o__Sphingomonadales;f__Sphingomonadaceae;g__	0.000	0.000	0.000	0.000	0.000	0.000	0.000	0.000	0.000	0.000	0.000	0.000	0.000	0.000	0.000	0.000	0.000	0.000	0.000	0.000	0.000	0.000	0.000	0.000	0.000	0.000	0.000	0.000	0.000	0.000	0.000	0.000	0.000	0.000	0.000	0.000	0.000	0.000	0.000	0.000	0.000	0.000	0.000	0.000	0.000	0.000	0.000	0.000	0.000	0.000	0.000	0.000	0.000	0.000	0.000	0.000	0.000	0.000	0.000	0.000
k__Bacteria;p__Proteobacteria;c__Alphaproteobacteria;o__Sphingomonadales;f__Sphingomonadaceae;g__Novosphingobium	0.000	0.000	0.000	0.000	0.000	0.000	0.000	0.000	0.000	0.000	0.000	0.000	0.000	0.000	0.000	0.000	0.000	0.000	0.000	0.000	0.000	0.000	0.000	0.000	0.000	0.000	0.000	0.000	0.000	0.000	0.000	0.000	0.000	0.000	0.000	0.000	0.000	0.000	0.000	0.000	0.000	0.000	0.000	0.000	0.000	0.000	0.000	0.000	0.000	0.000	0.000	0.000	0.000	0.000	0.000	0.000	0.000	0.000	0.000	0.000
k__Bacteria;p__Proteobacteria;c__Alphaproteobacteria;o__Sphingomonadales;f__Sphingomonadaceae;g__Sphingomonas	0.000	0.000	0.000	0.000	0.000	0.000	0.000	0.000	0.000	0.000	0.000	0.000	0.001	0.001	0.000	0.000	0.000	0.000	0.000	0.000	0.000	0.000	0.000	0.000	0.000	0.000	0.000	0.000	0.000	0.000	0.000	0.000	0.000	0.000	0.000	0.000	0.000	0.000	0.000	0.000	0.000	0.000	0.000	0.000	0.000	0.000	0.000	0.000	0.000	0.000	0.000	0.000	0.000	0.000	0.000	0.000	0.000	0.000	0.000	0.000
k__Bacteria;p__Proteobacteria;c__Betaproteobacteria;o__Burkholderiales;f__Alcaligenaceae;g__Sutterella	0.000	0.000	0.000	0.000	0.000	0.002	0.003	0.000	0.000	0.000	0.000	0.000	0.003	0.001	0.000	0.000	0.000	0.000	0.000	0.000	0.000	0.000	0.000	0.000	0.000	0.000	0.000	0.000	0.000	0.000	0.000	0.000	0.000	0.000	0.000	0.000	0.000	0.000	0.000	0.000	0.000	0.000	0.000	0.000	0.000	0.000	0.000	0.000	0.000	0.000	0.000	0.000	0.000	0.000	0.000	0.000	0.000	0.000	0.000	0.000
k__Bacteria;p__Proteobacteria;c__Betaproteobacteria;o__Burkholderiales;f__Comamonadaceae;g__	0.000	0.000	0.000	0.000	0.000	0.000	0.000	0.000	0.000	0.001	0.000	0.000	0.004	0.001	0.000	0.000	0.000	0.000	0.000	0.000	0.000	0.000	0.000	0.000	0.000	0.000	0.000	0.000	0.000	0.000	0.000	0.000	0.000	0.000	0.000	0.000	0.000	0.000	0.000	0.000	0.000	0.000	0.000	0.000	0.000	0.000	0.000	0.000	0.000	0.000	0.000	0.000	0.000	0.000	0.000	0.000	0.000	0.000	0.000	0.000
k__Bacteria;p__Proteobacteria;c__Betaproteobacteria;o__Neisseriales;f__Neisseriaceae;g__Neisseria	0.000	0.000	0.000	0.000	0.000	0.000	0.000	0.000	0.000	0.000	0.000	0.000	0.000	0.000	0.000	0.000	0.000	0.000	0.000	0.000	0.000	0.000	0.000	0.000	0.000	0.000	0.000	0.000	0.000	0.000	0.000	0.000	0.000	0.000	0.000	0.000	0.000	0.000	0.000	0.000	0.000	0.000	0.000	0.000	0.000	0.000	0.000	0.000	0.000	0.000	0.000	0.000	0.000	0.000	0.000	0.000	0.000	0.000	0.000	0.000
k__Bacteria;p__Proteobacteria;c__Betaproteobacteria;o__Rhodocyclales;f__Rhodocyclaceae;g__Hydrogenophilus	0.000	0.000	0.000	0.000	0.000	0.000	0.000	0.000	0.000	0.000	0.000	0.000	0.000	0.000	0.000	0.000	0.000	0.000	0.000	0.000	0.000	0.000	0.000	0.000	0.000	0.000	0.000	0.000	0.000	0.000	0.000	0.000	0.000	0.000	0.000	0.000	0.000	0.000	0.000	0.000	0.000	0.000	0.000	0.000	0.000	0.000	0.000	0.000	0.000	0.000	0.000	0.000	0.000	0.000	0.000	0.000	0.000	0.000	0.000	0.000
k__Bacteria;p__Proteobacteria;c__Deltaproteobacteria;o__Desulfovibrionales;f__Desulfovibrionaceae;g__	0.000	0.000	0.000	0.000	0.000	0.000	0.000	0.000	0.000	0.000	0.000	0.000	0.000	0.000	0.000	0.000	0.000	0.000	0.000	0.000	0.000	0.000	0.000	0.000	0.000	0.000	0.000	0.000	0.000	0.000	0.000	0.000	0.000	0.000	0.000	0.000	0.000	0.000	0.000	0.000	0.000	0.000	0.000	0.000	0.000	0.000	0.000	0.000	0.000	0.000	0.000	0.000	0.000	0.000	0.000	0.000	0.000	0.000	0.000	0.000
k__Bacteria;p__Proteobacteria;c__Deltaproteobacteria;o__Desulfovibrionales;f__Desulfovibrionaceae;g__Bilophila	0.000	0.000	0.000	0.000	0.000	0.000	0.000	0.000	0.000	0.000	0.000	0.002	0.000	0.000	0.000	0.000	0.000	0.000	0.000	0.000	0.000	0.000	0.000	0.000	0.000	0.000	0.000	0.000	0.000	0.000	0.000	0.000	0.000	0.000	0.000	0.000	0.000	0.000	0.000	0.000	0.000	0.000	0.000	0.000	0.000	0.000	0.000	0.000	0.000	0.000	0.000	0.000	0.000	0.000	0.000	0.000	0.000	0.000	0.000	0.000
k__Bacteria;p__Proteobacteria;c__Deltaproteobacteria;o__Desulfovibrionales;f__Desulfovibrionaceae;g__Desulfovibrio	0.000	0.000	0.000	0.000	0.000	0.000	0.000	0.000	0.000	0.000	0.000	0.000	0.001	0.000	0.000	0.000	0.000	0.000	0.000	0.000	0.000	0.000	0.000	0.000	0.000	0.000	0.000	0.000	0.000	0.000	0.000	0.000	0.000	0.000	0.000	0.000	0.000	0.000	0.000	0.000	0.000	0.000	0.000	0.000	0.000	0.000	0.000	0.000	0.000	0.000	0.000	0.000	0.000	0.000	0.000	0.000	0.000	0.000	0.000	0.000
k__Bacteria;p__Proteobacteria;c__Epsilonproteobacteria;o__Campylobacterales;f__Campylobacteraceae;g__Campylobacter	0.000	0.000	0.000	0.000	0.000	0.012	0.000	0.000	0.000	0.000	0.000	0.000	0.000	0.008	0.000	0.000	0.000	0.000	0.000	0.000	0.000	0.000	0.000	0.000	0.000	0.000	0.000	0.000	0.000	0.000	0.000	0.000	0.000	0.000	0.000	0.000	0.000	0.000	0.000	0.000	0.000	0.000	0.000	0.000	0.000	0.000	0.000	0.000	0.000	0.000	0.000	0.000	0.000	0.000	0.000	0.000	0.000	0.000	0.000	0.000
k__Bacteria;p__Proteobacteria;c__Gammaproteobacteria;o__Aeromonadales;f__Succinivibrionaceae;g__Succinivibrio	0.000	0.000	0.000	0.000	0.000	0.000	0.000	0.000	0.000	0.000	0.000	0.000	0.000	0.000	0.000	0.000	0.000	0.000	0.000	0.000	0.000	0.000	0.000	0.000	0.000	0.000	0.000	0.000	0.000	0.000	0.000	0.000	0.000	0.000	0.000	0.000	0.000	0.000	0.000	0.000	0.000	0.000	0.000	0.000	0.000	0.000	0.000	0.000	0.000	0.000	0.000	0.000	0.000	0.000	0.000	0.000	0.000	0.000	0.000	0.000
k__Bacteria;p__Proteobacteria;c__Gammaproteobacteria;o__Enterobacteriales;f__Enterobacteriaceae;g__	0.000	0.001	0.001	0.000	0.000	0.001	0.004	0.000	0.000	0.011	0.000	0.017	0.011	0.027	0.001	0.000	0.015	0.000	0.000	0.000	0.000	0.000	0.000	0.000	0.000	0.000	0.000	0.000	0.377	0.000	0.000	0.000	0.000	0.000	0.000	0.773	0.000	0.000	0.000	0.073	0.000	0.000	0.003	0.146	0.000	0.012	0.003	0.050	0.095	0.158	0.362	0.001	0.128	0.226	0.018	0.267	0.091	0.003	0.516	0.117
k__Bacteria;p__Proteobacteria;c__Gammaproteobacteria;o__Enterobacteriales;f__Enterobacteriaceae;g__Citrobacter	0.000	0.000	0.000	0.000	0.000	0.000	0.000	0.000	0.000	0.000	0.000	0.000	0.000	0.000	0.000	0.000	0.000	0.000	0.000	0.000	0.000	0.000	0.000	0.000	0.000	0.000	0.000	0.000	0.000	0.000	0.000	0.000	0.000	0.000	0.000	0.000	0.000	0.000	0.000	0.000	0.000	0.000	0.000	0.000	0.000	0.000	0.000	0.000	0.000	0.000	0.000	0.000	0.000	0.000	0.000	0.000	0.001	0.020	0.000	0.000
k__Bacteria;p__Proteobacteria;c__Gammaproteobacteria;o__Enterobacteriales;f__Enterobacteriaceae;g__Enterobacter	0.000	0.000	0.000	0.000	0.000	0.000	0.000	0.000	0.000	0.000	0.000	0.000	0.000	0.000	0.000	0.000	0.000	0.000	0.000	0.000	0.000	0.000	0.000	0.000	0.000	0.000	0.000	0.000	0.000	0.000	0.000	0.000	0.000	0.000	0.000	0.000	0.000	0.000	0.000	0.000	0.000	0.000	0.000	0.000	0.000	0.000	0.000	0.000	0.000	0.000	0.000	0.033	0.000	0.000	0.000	0.000	0.000	0.000	0.000	0.000
k__Bacteria;p__Proteobacteria;c__Gammaproteobacteria;o__Enterobacteriales;f__Enterobacteriaceae;g__Klebsiella	0.000	0.000	0.000	0.000	0.000	0.000	0.000	0.000	0.000	0.000	0.000	0.000	0.000	0.000	0.000	0.000	0.000	0.000	0.000	0.000	0.000	0.000	0.000	0.000	0.000	0.000	0.000	0.000	0.000	0.000	0.000	0.000	0.000	0.000	0.000	0.000	0.000	0.000	0.000	0.000	0.000	0.000	0.000	0.001	0.000	0.000	0.000	0.000	0.000	0.000	0.000	0.000	0.000	0.000	0.000	0.000	0.000	0.000	0.000	0.000
k__Bacteria;p__Proteobacteria;c__Gammaproteobacteria;o__Enterobacteriales;f__Enterobacteriaceae;g__Proteus	0.000	0.000	0.000	0.000	0.000	0.000	0.000	0.000	0.000	0.000	0.000	0.000	0.000	0.000	0.000	0.000	0.000	0.000	0.000	0.000	0.000	0.000	0.000	0.000	0.000	0.000	0.000	0.000	0.000	0.000	0.000	0.000	0.000	0.000	0.000	0.000	0.000	0.000	0.000	0.000	0.000	0.000	0.000	0.000	0.000	0.000	0.000	0.000	0.000	0.000	0.004	0.000	0.000	0.000	0.000	0.000	0.000	0.000	0.000	0.000
k__Bacteria;p__Proteobacteria;c__Gammaproteobacteria;o__Enterobacteriales;f__Enterobacteriaceae;g__Trabulsiella	0.000	0.000	0.000	0.000	0.000	0.000	0.000	0.000	0.000	0.000	0.000	0.000	0.000	0.000	0.000	0.000	0.000	0.000	0.000	0.000	0.000	0.000	0.000	0.000	0.000	0.000	0.000	0.000	0.000	0.000	0.000	0.000	0.000	0.000	0.000	0.000	0.000	0.000	0.000	0.000	0.000	0.000	0.000	0.000	0.000	0.000	0.000	0.000	0.000	0.000	0.000	0.000	0.003	0.000	0.000	0.000	0.000	0.000	0.000	0.000
k__Bacteria;p__Proteobacteria;c__Gammaproteobacteria;o__Enterobacteriales;f__Enterobacteriaceae;Other	0.000	0.000	0.000	0.000	0.000	0.000	0.000	0.000	0.000	0.000	0.000	0.000	0.000	0.000	0.000	0.000	0.000	0.000	0.000	0.000	0.000	0.000	0.000	0.000	0.000	0.000	0.000	0.000	0.000	0.000	0.000	0.000	0.000	0.000	0.000	0.000	0.000	0.000	0.000	0.000	0.000	0.000	0.000	0.000	0.000	0.000	0.000	0.000	0.000	0.000	0.000	0.000	0.000	0.000	0.000	0.000	0.000	0.000	0.000	0.000
k__Bacteria;p__Proteobacteria;c__Gammaproteobacteria;o__Legionellales;f__Coxiellaceae;g__	0.000	0.000	0.000	0.000	0.000	0.000	0.000	0.000	0.000	0.000	0.000	0.000	0.000	0.000	0.000	0.000	0.000	0.000	0.000	0.000	0.000	0.000	0.000	0.000	0.000	0.000	0.000	0.000	0.000	0.000	0.000	0.000	0.000	0.000	0.000	0.000	0.000	0.000	0.000	0.000	0.000	0.000	0.000	0.000	0.000	0.000	0.000	0.000	0.007	0.000	0.000	0.000	0.000	0.000	0.000	0.000	0.000	0.000	0.000	0.000
k__Bacteria;p__Proteobacteria;c__Gammaproteobacteria;o__Pasteurellales;f__Pasteurellaceae;g__Haemophilus	0.000	0.000	0.000	0.000	0.000	0.005	0.001	0.000	0.000	0.000	0.000	0.000	0.001	0.002	0.000	0.000	0.000	0.000	0.000	0.000	0.000	0.000	0.000	0.000	0.000	0.000	0.000	0.000	0.000	0.000	0.000	0.000	0.000	0.000	0.000	0.000	0.000	0.000	0.000	0.000	0.000	0.000	0.000	0.000	0.000	0.000	0.000	0.000	0.000	0.000	0.000	0.000	0.000	0.000	0.000	0.000	0.000	0.000	0.000	0.000
k__Bacteria;p__Proteobacteria;c__Gammaproteobacteria;o__Pseudomonadales;f__Moraxellaceae;g__Acinetobacter	0.000	0.000	0.000	0.000	0.000	0.000	0.000	0.000	0.000	0.000	0.000	0.000	0.006	0.001	0.000	0.000	0.000	0.000	0.000	0.000	0.000	0.000	0.000	0.000	0.000	0.000	0.000	0.000	0.000	0.000	0.000	0.000	0.000	0.000	0.000	0.000	0.000	0.000	0.000	0.000	0.000	0.000	0.000	0.000	0.000	0.000	0.000	0.000	0.000	0.000	0.000	0.000	0.000	0.000	0.000	0.000	0.000	0.000	0.000	0.000
k__Bacteria;p__Proteobacteria;c__Gammaproteobacteria;o__Pseudomonadales;f__Moraxellaceae;g__Enhydrobacter	0.000	0.000	0.000	0.000	0.000	0.000	0.000	0.000	0.000	0.000	0.000	0.000	0.000	0.000	0.000	0.000	0.000	0.000	0.000	0.000	0.000	0.000	0.000	0.000	0.000	0.000	0.000	0.000	0.000	0.000	0.000	0.000	0.000	0.000	0.000	0.000	0.000	0.000	0.000	0.000	0.000	0.000	0.000	0.000	0.000	0.000	0.000	0.000	0.000	0.000	0.000	0.000	0.000	0.000	0.000	0.000	0.000	0.000	0.000	0.000
k__Bacteria;p__Proteobacteria;c__Gammaproteobacteria;o__Pseudomonadales;f__Pseudomonadaceae;g__	0.000	0.000	0.000	0.000	0.000	0.000	0.000	0.000	0.000	0.000	0.000	0.000	0.000	0.000	0.000	0.000	0.000	0.000	0.000	0.000	0.000	0.000	0.000	0.000	0.000	0.000	0.000	0.000	0.000	0.000	0.000	0.000	0.000	0.000	0.000	0.000	0.000	0.000	0.000	0.000	0.000	0.000	0.000	0.000	0.000	0.000	0.000	0.000	0.000	0.000	0.000	0.000	0.000	0.000	0.000	0.000	0.000	0.000	0.000	0.000
k__Bacteria;p__Proteobacteria;c__Gammaproteobacteria;o__Pseudomonadales;f__Pseudomonadaceae;g__Pseudomonas	0.000	0.000	0.000	0.000	0.000	0.000	0.000	0.000	0.000	0.000	0.000	0.000	0.001	0.001	0.000	0.000	0.000	0.000	0.000	0.002	0.002	0.000	0.000	0.000	0.000	0.002	0.000	0.000	0.000	0.000	0.000	0.000	0.000	0.000	0.000	0.000	0.000	0.000	0.000	0.000	0.000	0.000	0.000	0.000	0.000	0.000	0.000	0.000	0.000	0.000	0.000	0.000	0.000	0.000	0.000	0.000	0.000	0.000	0.000	0.000
k__Bacteria;p__Proteobacteria;c__Gammaproteobacteria;o__Xanthomonadales;f__Xanthomonadaceae;g__	0.000	0.000	0.000	0.000	0.000	0.000	0.000	0.000	0.000	0.000	0.000	0.000	0.000	0.000	0.000	0.000	0.000	0.000	0.000	0.000	0.000	0.000	0.000	0.000	0.000	0.000	0.000	0.000	0.000	0.000	0.000	0.000	0.000	0.000	0.000	0.000	0.000	0.000	0.000	0.000	0.000	0.000	0.000	0.000	0.000	0.000	0.000	0.000	0.000	0.000	0.000	0.000	0.000	0.000	0.000	0.000	0.000	0.000	0.000	0.000
k__Bacteria;p__Proteobacteria;c__Gammaproteobacteria;o__Xanthomonadales;f__Xanthomonadaceae;g__Stenotrophomonas	0.000	0.000	0.000	0.000	0.000	0.000	0.000	0.000	0.000	0.000	0.000	0.000	0.000	0.000	0.000	0.000	0.000	0.000	0.000	0.000	0.000	0.000	0.000	0.000	0.000	0.000	0.000	0.000	0.000	0.000	0.000	0.000	0.000	0.000	0.000	0.000	0.000	0.000	0.000	0.000	0.000	0.000	0.000	0.000	0.000	0.000	0.000	0.000	0.000	0.000	0.000	0.000	0.000	0.000	0.000	0.000	0.000	0.000	0.000	0.000
k__Bacteria;p__Synergistetes;c__Synergistia;o__Synergistales;f__Dethiosulfovibrionaceae;g__Jonquetella	0.000	0.000	0.000	0.000	0.000	0.000	0.000	0.000	0.000	0.000	0.000	0.000	0.000	0.000	0.000	0.000	0.000	0.000	0.000	0.000	0.000	0.000	0.000	0.000	0.000	0.000	0.000	0.000	0.000	0.000	0.000	0.000	0.000	0.000	0.000	0.000	0.000	0.000	0.000	0.000	0.000	0.000	0.000	0.000	0.000	0.000	0.000	0.000	0.000	0.000	0.000	0.000	0.000	0.000	0.000	0.000	0.000	0.000	0.000	0.000
k__Bacteria;p__Synergistetes;c__Synergistia;o__Synergistales;f__Dethiosulfovibrionaceae;g__Pyramidobacter	0.000	0.000	0.000	0.000	0.000	0.000	0.000	0.000	0.000	0.000	0.000	0.000	0.000	0.000	0.000	0.000	0.000	0.000	0.000	0.000	0.000	0.000	0.000	0.000	0.000	0.000	0.000	0.000	0.000	0.000	0.000	0.000	0.000	0.000	0.000	0.000	0.000	0.000	0.000	0.000	0.000	0.000	0.000	0.000	0.000	0.000	0.000	0.000	0.000	0.000	0.000	0.000	0.000	0.000	0.000	0.000	0.000	0.000	0.000	0.000
k__Bacteria;p__Tenericutes;c__Mollicutes;o__Mycoplasmatales;f__Mycoplasmataceae;g__Ureaplasma	0.003	0.000	0.000	0.000	0.000	0.002	0.000	0.000	0.006	0.000	0.000	0.003	0.000	0.000	0.000	0.000	0.000	0.000	0.000	0.000	0.000	0.000	0.000	0.000	0.000	0.000	0.000	0.000	0.000	0.000	0.000	0.000	0.000	0.000	0.000	0.000	0.000	0.000	0.000	0.000	0.000	0.000	0.000	0.000	0.000	0.000	0.000	0.000	0.000	0.000	0.000	0.000	0.000	0.000	0.000	0.000	0.000	0.000	0.000	0.000
k__Bacteria;p__Tenericutes;c__Mollicutes;o__RF39;f__;g__	0.000	0.000	0.000	0.000	0.000	0.000	0.000	0.000	0.000	0.000	0.000	0.000	0.000	0.000	0.000	0.000	0.000	0.000	0.000	0.000	0.000	0.000	0.000	0.000	0.000	0.000	0.000	0.000	0.000	0.000	0.000	0.000	0.000	0.000	0.000	0.000	0.000	0.000	0.000	0.000	0.000	0.000	0.000	0.000	0.000	0.000	0.000	0.000	0.000	0.000	0.000	0.000	0.000	0.000	0.000	0.000	0.000	0.000	0.000	0.000
k__Bacteria;p__Tenericutes;c__RF3;o__ML615J-28;f__;g__	0.000	0.000	0.000	0.000	0.000	0.000	0.000	0.000	0.000	0.000	0.000	0.000	0.000	0.000	0.000	0.000	0.000	0.000	0.000	0.000	0.000	0.000	0.000	0.000	0.000	0.000	0.000	0.000	0.000	0.000	0.000	0.000	0.000	0.000	0.000	0.000	0.000	0.000	0.000	0.000	0.000	0.000	0.000	0.000	0.000	0.000	0.000	0.000	0.000	0.000	0.000	0.000	0.000	0.000	0.000	0.000	0.000	0.000	0.000	0.000
k__Bacteria;p__Verrucomicrobia;c__Verrucomicrobiae;o__Verrucomicrobiales;f__Verrucomicrobiaceae;g__Akkermansia	0.000	0.000	0.000	0.000	0.000	0.000	0.000	0.000	0.000	0.000	0.000	0.000	0.000	0.000	0.000	0.000	0.000	0.000	0.000	0.000	0.000	0.000	0.000	0.000	0.000	0.000	0.000	0.000	0.000	0.000	0.000	0.000	0.000	0.000	0.000	0.000	0.000	0.000	0.000	0.000	0.000	0.000	0.000	0.000	0.000	0.000	0.000	0.000	0.000	0.000	0.000	0.000	0.000	0.000	0.000	0.000	0.000	0.000	0.000	0.000
Unassigned;Other;Other;Other;Other;Other	0.000	0.000	0.000	0.000	0.000	0.001	0.000	0.000	0.000	0.000	0.000	0.000	0.000	0.000	0.000	0.000	0.000	0.000	0.000	0.000	0.000	0.000	0.000	0.000	0.000	0.000	0.000	0.000	0.000	0.000	0.000	0.000	0.000	0.000	0.000	0.000	0.001	0.000	0.000	0.000	0.000	0.000	0.000	0.000	0.000	0.000	0.000	0.000	0.000	0.000	0.000	0.000	0.001	0.000	0.002	0.000	0.001	0.000	0.000	0.001

Supplemental Table 8. List of primer sets.

Primer	Target	Sequence (5' to 3')	Reference
Tru357F	V3-V4 region of the 16S rRNA gene	CGCTCTTCCGATCTCTGTACGGRAGGCAGCAG	
Tru806R		CGCTCTTCCGATCTGACGGACTACHVGGGTWTCTA	
g-Bifid-F	*Bifidobacterium*	CTCCTGGAAACGGGTGG	*Matsuki T et al.,* Appl Environ Microbiol. 68, 11, 5445-5451 (2002).
g-Bifid-R		GGTGTTCTTCCCGATATCTACA	
BiADOg-1a	*Bifidobacterium adolescentis* group	CTCCAGTTGGATGCATGTC	*Matsuki T et al.,* Appl Environ Microbiol. 65, 10, 4506-4512 (1999).
BiADOg-1b		TCCAGTTGACCGCATGGT	
BiADOg-2		CGAAGGCTTGCTCCCAGT	
Bflact2	*Bifidobacterium animalis* ssp. *lactis*	GTGGAGACACGGTTTCCC	*Ventura M et al.,* Appl Environ Microbiol. 67, 6, 2760-2765 (2001).
Bflact5		CACACCACACAATCCAATAC	
BiBIF-1	*Bifidobacterium bifidum*	CCACATGATCGCATGTGATTG	*Matsuki T et al.,* FEMS Microbiol Lett. 167, 2, 113-121 (1998).
BiBIF-2		CCGAAGGCTTGCTCCCAAA	
BiBRE-1	*Bifidobacterium breve*	CCGGATGCTCCATCACAC	*Matsuki T et al.,* FEMS Microbiol Lett. 167, 2, 113-121 (1998).
BiBRE-2		ACAAAGTGCCTTGCTCCCT	
BiCATg-1	*Bifidobacterium catenulatum* group	CGGATGCTCCGACTCCT	*Matsuki T et al.,* FEMS Microbiol Lett. 167, 2, 113-121 (1998).
BiCATg-2		CGAAGGCTTGCTCCCGAT	
BiDEN-1	*Bifidobacterium dentium*	ATCCCGGGGGTTCGCCT	*Matsuki T et al.,* Appl Environ Microbiol. 65, 10, 4506-4512 (1999).
BiDEN-2		GAAGGGCTTGCTCCCGA	
BiLON-1	*Bifidobacterium longum* ssp. *longum*	TTCCAGTTGATCGCATGGTC	*Matsuki T et al.,* Appl Environ Microbiol. 65, 10, 4506-4512 (1999).
BiLON-2		GGGAAGCCGTATCTCTACGA	
INF_clpC_F	*Bifidobacterium longum* ssp. *infantis*	ACATCCAGGACCGTAACCTG	In this study
INF_clpC_R		GCTTGTGCAGCTCCGTCT	
OPF-09	RAPD-PCR (primer type 1)	CCAAGCTTCC	
OPF-11	RAPD-PCR (primer type 2)	TTGGTACCCC	
